# Juggling Optoelectronics and Catalysis: The Dual Talents of Bench Stable 1,4‐Azaborinines

**DOI:** 10.1002/chem.202301944

**Published:** 2023-12-22

**Authors:** Chloe M. van Beek, Amelia M. Swarbrook, Charles E. Creissen, Chris S. Hawes, Theodore A. Gazis, Peter D. Matthews

**Affiliations:** ^1^ School of Chemical & Physical Sciences Keele University Newcastle-under-Lyme, Staffs ST5 5BG U.K.

**Keywords:** polycyclic aromatic hydrocarbons, boron heterocycles, optoelectronic properties, catalysis, hydroboration, azaborinine

## Abstract

Boron‐ and nitrogen‐doped polycyclic aromatic hydrocarbons (B‐PAHs) have established a strong foothold in the realm of organic electronics. However, their catalytic potential remains largely untapped. In this study, we synthesise and characterise two bench stable B,N‐doped PAH derivatives based on a 1,4‐azaborinine motif. Most importantly, the anthracene derived structure is an efficient catalyst in the reduction of various carbonyls and imines. These results underscore the potential of B,N‐PAHs in catalytic transformations, setting the stage for deeper exploration in this chemical space.

## Graphical abstract

Bench stable 1,4‐azaborinine PAHs with an anthracene or ladder‐type pentacene scaffold have been prepared, characterised and proven to be efficient catalysts for hydroboration of carbonyls and imines.

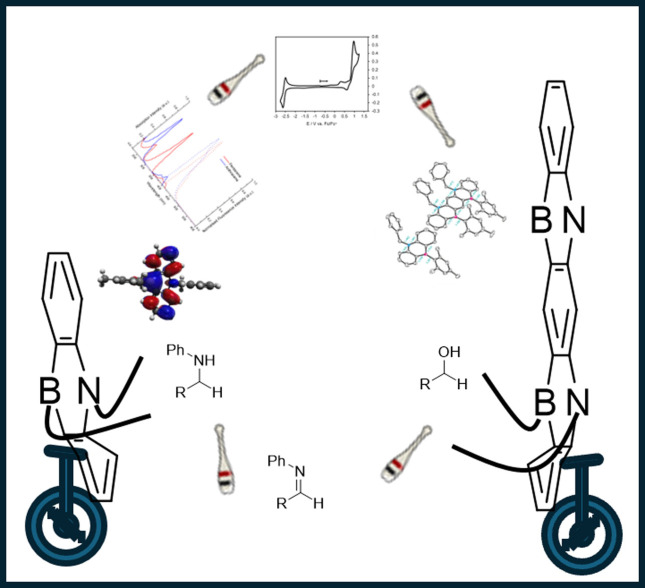

## Introduction

More than half a century has elapsed since the initial characterisation of polycyclic aromatic hydrocarbons (PAHs), yet their conductive and photophysical properties remain a focal point of scientific exploration and interest. This is generally attributed to their low HOMO‐LUMO gap, propensity to self‐assemble and general affordability.^[1]^ However, their greatest strength is also their Achilles heel. Conventional carbon‐based PAHs cannot have their bandgap modulated sufficiently to keep pace with the myriad of potential applications. To address this challenge, two synergistic strategies have been devised: firstly, structural modulation either by bond compression or bulky substituents, facilitates size and shape control, enhances stability and permits alteration of numerous other properties. Secondly heteroatom doping with boron, silicon, phosphorus and nitrogen allows for further tailoring of the reactivity, structure and electronic characteristics.^[2]^

The inclusion of boron (B‐PAHs) or co‐doping with boron and nitrogen (B,N‐PAHs) occupy a prominent position in this chemical space. Their structure‐property relationship alongside the relative simplicity of their synthesis, has cemented their dominance in the field of organic electronics. with applications spanning domains such as display technology, pigments and chemical sensing.[^3^, ^4^, ^5^, ^6^, ^7^, ^8^, ^9^, ^10^, ^11^, ^12^, ^13^]

**Figure 1:**
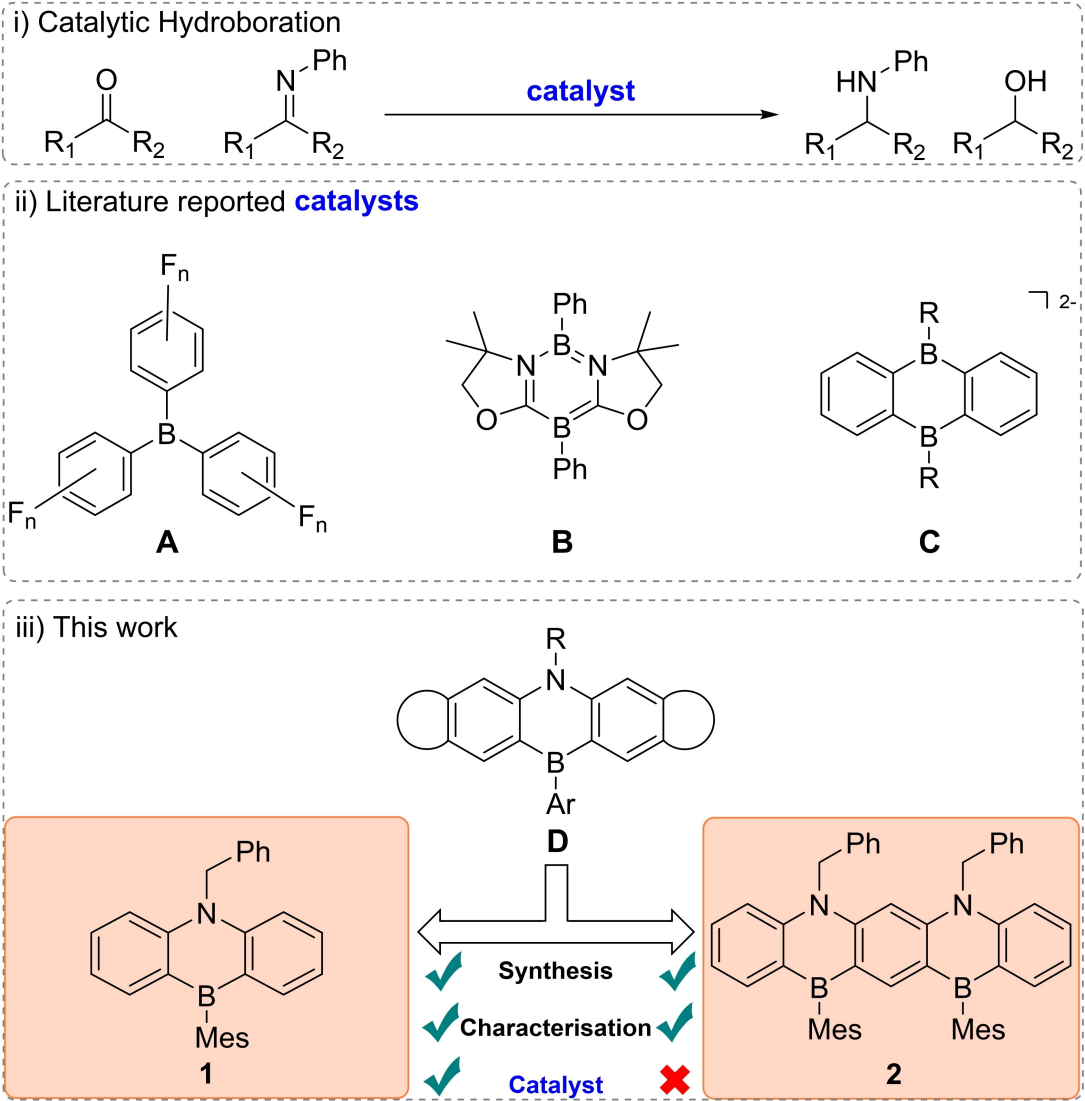
Previous arylborane catalysts employed in hydroboration catalysis (**A**–**C**). Bench stable 1,4‐azaborinines **1** and **2** based on structural motif **D** and their application in catalysis.

Given the substantial commercial appeal of their optoelectronic properties, the synthetic inclination of B/B,N‐PAHs frequently becomes a secondary concern. Indeed, most synthetic reports focus on structural alterations with the goal of fine‐tuning their inherent properties.[^14^, ^15^, ^16^, ^17^, ^18^, ^19^, ^20^] From the limited synthetic reports, noteworthy is the exploration of stoichiometric activation of small molecules, a particularly fruitful research direction.^[21]^ Contrarily, the catalytic potential of B/B,N‐PAHs remains largely untapped. Isolated examples include a 1,4‐azaborinine utilized as a catalyst for triarylphosphine photooxidation,^[22]^ and a 1,2‐azaborinine serving as an electrocatalyst in the oxygen reduction reaction (ORR).^[23]^ The lack of catalytic studies stands in stark contrast with other non‐conjugated arylboranes of type **A** (Figure 1), such as tris(pentafluorophenyl)borane (BCF), whose catalytic prowess in olefin polymerisation, elementoboration *etc*., is well documented.^[24]^ In particular, the use of fluorinated triarylboranes in hydroboration catalysis is near ubiquitous, with this reaction often employed as a benchmark test to evaluate the performance of novel borane reagents.^[25]^

To date, there are two documented reports on catalytic transformations involving conjugated B‐PAHs. The Kinjo group successfully utilized aromatic diazadiborinine **B** (Figure 1) to hydroborate a diverse library of carbonyl compounds as well as CO_2_.^[26]^ Building on this work, the Wagner group utilised an ionic 9,10‐diboroanthracene **C** (Figure 1) to broaden the scope to additional unsaturated substrates.^[27]^ However, it should be noted both B‐PAH catalysts are susceptible to moisture, a trait they share with the majority of triarylborane hydroboration catalysts.^[25]^

In this contribution we describe the synthesis and optoelectronic characterisation of two novel azaborini derived from established structural motif **D**, recognised for its air stability.^[28]^ Mirroring their parent structure, our derivatives exhibited no discernible degradation over a 12‐month period despite storage under ambient conditions. Additionally, we explored their catalytic efficiency in the reduction of ketones, aldehydes and imines.

## Results and Discussion

**Scheme 1:**
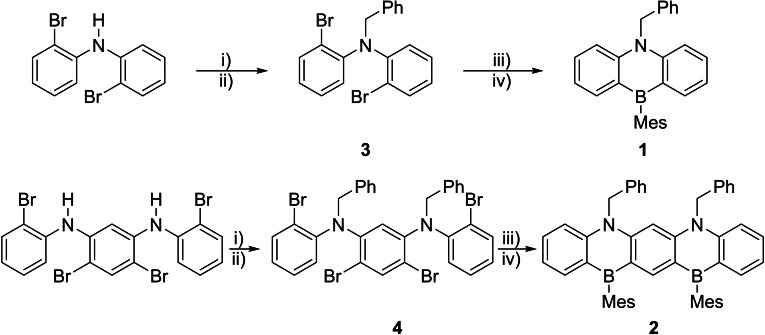
Reaction pathway employed for the formation of **1** and **2**. i) NaH, THF/1,4‐dioxane. ii) BnBr, reflux. iii) ^n^BuLi, Et_2_O, −78 °C. iv) MesB(OMe)_2_, reflux.

Compound 1 was furnished in two steps using bis(2‐bromophenyl)amine as the starting material (Scheme 1). The benzyl protection of the amine functionality generated derivative **3** (ESI Figure S37). A simple borylation involving ^n^BuLi and dimethyl(mesityl)boronate followed with desired compound **1** being sufficiently stable to be purified by column chromatography in excellent yields (86 %). Pentacene derivative **2** was synthesized in an analogous fashion employing compound **4** (ESI Figure S38) as an intermediate (Scheme 1). Column chromatography was once again effective in purifying **2**, yielding a modest 13 % of product.

**Figure 2:**
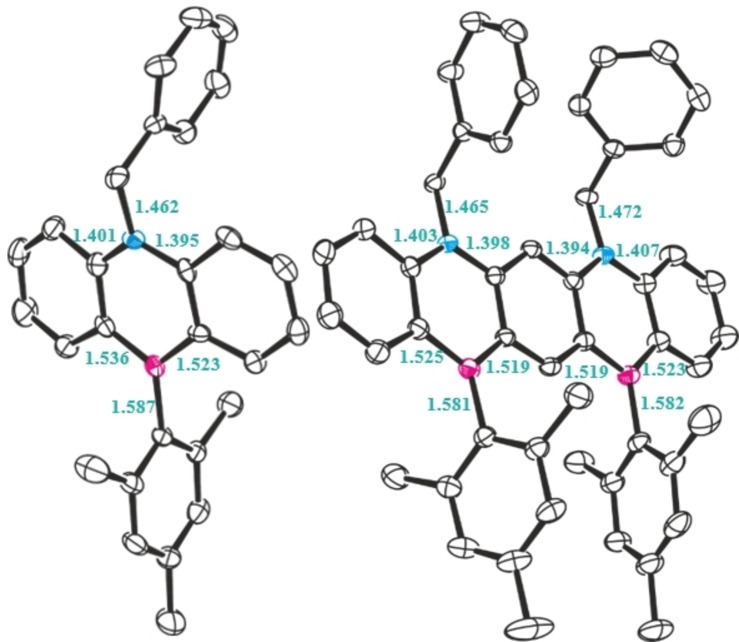
Molecular structures and key bond metrics of **1** (left) and **2** (right). Thermal ellipsoids drawn at 50 %. H atoms omitted for clarity. Carbon: black; Nitrogen: blue; Boron: pink.

The solid‐state structures of both derivatives were definitively ascertained by single crystal X‐ray crystallography. Recrystallization from hexane afforded compound **1** as white crystals, adopting the monoclinic crystal system and the P2_1_/c space group (Figure 2, left). Both the boron and nitrogen heteroatoms display trigonal planar arrangements, featuring bond angle sums of 359.9° – a testament to efficient conjugation across the anthracene core. Indeed, as typical for azaborinines, full conjugation around the central anthracene unit dictates a high degree of planarity with a mean deviation of 0.043(2) Å. This value is marginally higher than for a reported dibenzoazaborine derivative featuring a methyl group on the nitrogen atom.^[29]^ Finally, the benzyl and mesityl functionalities lie outside the plane to minimize steric interactions.

Ladder type molecule **2** was crystallised from a toluene/hexane mixture in the P1^−^ space group (Figure 2, right), The bond metrics were comparable to **1** (ESI Figures S39 and S40) but it possesses a lower deviation from planarity [0.006 and 0.017(17) Å], suggesting full conjugation across the pentacene core. A notable deviation from previous reports is the presence of a CH interaction between the benzyl CH_2_ and a neighbouring anthracene ring. This intermolecular van der Waals interaction enables compound **2** to pack in sheets (ESI Figure S40b).^[30]^

**Figure 3:**
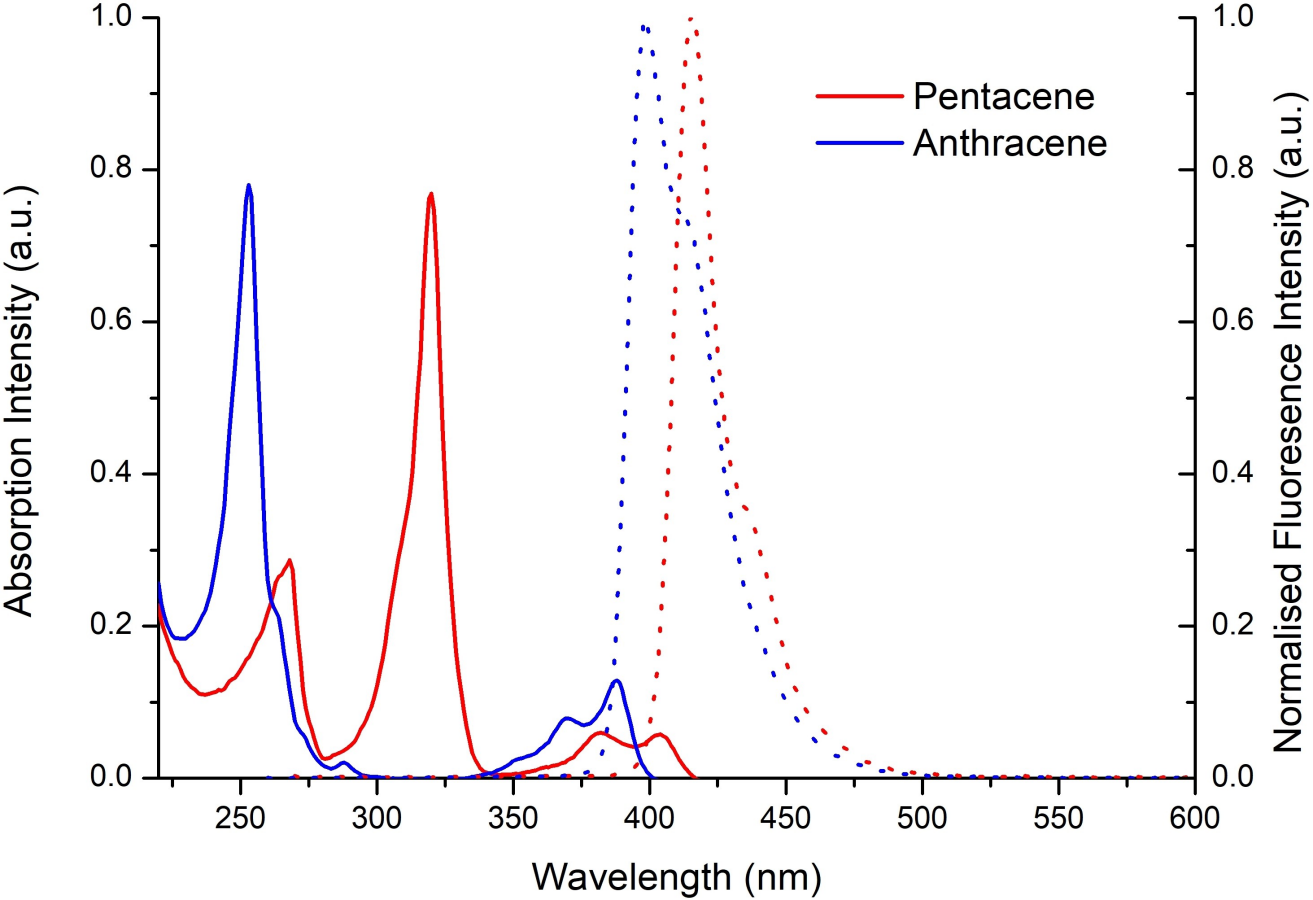
UV‐vis absorbance spectra (solid traces) and fluorescence spectra (dashed traces) of **1** and **2**.

Next, we conducted a comparative analysis of the photophysical properties of compounds **1** and **2**, utilizing hexane as the solvent. The UV‐visible absorption spectra of both derivatives displayed four absorption band peaks (λ_abs_) with maxima at 254 nm (**1**) and 320 nm (**2**) (Figure 3, solid traces). Despite this red shift in absorption maximum for the larger **2**, significant peak overlap between 350–400 nm is evident. This observation indicates limited additional π‐conjugation for **2** over **1**, as previously reported by Agou *et al*. for their analogous anthracene and pentacene derivatives.[^30^, ^31^] Consequently, the spectral similarities denote comparable energy level transitions for both materials, albeit with varying absorption intensities.

Providing further substantiation to this hypothesis, the fluorescence spectra of compounds **1** and **2** are visually similar (Figure 3, dashed traces). The primary distinction is the notably weaker and offset blue emission of compound **2** (λ_em_=416 nm, Φ_F_=0.22) in comparison to **1** (λ_em_=399 nm, Φ_F_=0.7). We ascribe the low quantum yield (Φ_F_) of **2** to energy transfer between the two neighbouring mesityl groups in the excited state, a phenomenon previously documented.[^30^, ^31^]

Cyclic voltammetry (CV) was used to explore the electrochemical behaviour of the two compounds (Table 1, ESI Table S3, Figure S41). Both showed irreversible redox features upon oxidation, with primary oxidation peaks at +0.33 V and −0.03 V vs. Fc/Fc^+^ for **1** and **2** respectively, demonstrating that **2** has a higher energy HOMO and is therefore more easily oxidised than **1**. Although both **1** and **2** displayed additional irreversible features at more positive potentials, these resulted in loss of reduction peaks and were attributed to decomposition. The irreversible behaviour suggests that the radical cation is unstable and likely dimerizes as has been observed with similar species.^[28]^ Upon reduction, **1** displayed a reversible redox feature (E_1/2_=−2.57 V vs. Fc/Fc^+^), while **2** gave rise to two quasi‐reversible features (E_p_=−2.61 and −2.85 V vs. Fc/Fc^+^), which can be attributed to consecutive electron transfer steps (EE) as observed with similar compounds.^[32]^

**Table 1:** Optical and electrochemical properties of **1** and **2**.

Compound	λ_max_ [nm]	λ_em_ [nm]	Φ_F_^a^	HOMO [eV]	LUMO [eV]	Eg [eV]
1	254	399	0.7b	−5.13	−2.23	2.9
2	320	416	0.22c	−4.77	−2.19	2.7

^[a]^ Quantum yields were measured in toluene with quinine in 0.5 M H_2_SO_4_ as the standard. ^[b]^ Excitation at 374 nm. ^[c]^ Excitation at 324 nm.

The vast majority of triarylboranes utilized in hydroboration catalysis necessitate cautious handling to avoid moisture exposure. The notable air stability of **1** and **2**, prompted a thorough investigation of their potential in hydroboration catalysis. Catalyst optimization was conducted with 4‐(trifluoromethyl)benzaldehyde and HBpin in CDCl_3_ as model substrates. In the absence of catalyst (but presence of solvent) at room temperature, negligible conversion was observed, in agreement with previous results.^[34]^ Promisingly, introduction of **1** at 5 % cat. loading resulted in 30 % conversion over 24 h, albeit in a kinetically slow manner. Increasing the catalyst loading to 10 or 20 % did not yield significant improvements (34 % and 38 % respectively). A similar reaction profile emerged when swapping the reaction solvent to C_6_D_6_ (39 %), toluene (21 %) and tetrahydrofuran (44 %). However, quantitative conversion was achieved within 3 hours upon raising the temperature to 70 °C in CDCl_3_. We confirmed that the reactivity was due to catalytic hydroboration by HBpin and not *via* a nucleophile‐promoted BH_3_ formation by including TMEDA in the reaction mixture and attaining similar conversion, whilst not observing the formation of BH_3_.^[33]^ Testing azaborinine **2** as an alternative catalyst resulted in a conversion drop to <5 %, which is somewhat surprising. Looking at the frontier molecular orbitals suggests that there is a substantial difference in the HOMOs of **1** and **2**. For **1** the HOMO has components on both the nitrogen and boron, whilst **2** is exclusively on the boron. On the other hand, the LUMOs of both the **1** and **2** are quite similar, with the major component centred on the boron as expected, although for **1** the nitrogen makes a minor contribution (ESI Figure S42). Furthermore, it is well known that aromatics substituted at the 9 position of anthracene have restricted rotation owing to clash with 1,4,5,8 hydrogens,[^35^, ^36^, ^37^] and this effect is magnified by the clashing groups of **2**. In order to access the reactive sites on the B/N atoms, the mesityl and benzyl groups respectively must rotate out the way, however this is conformationally more challenging in **2**. This interesting outcome is worthy of further study, and future work will look to probe this reactivity difference further through more detailed DFT kinetic isotope effect studies.

**Figure 4:**
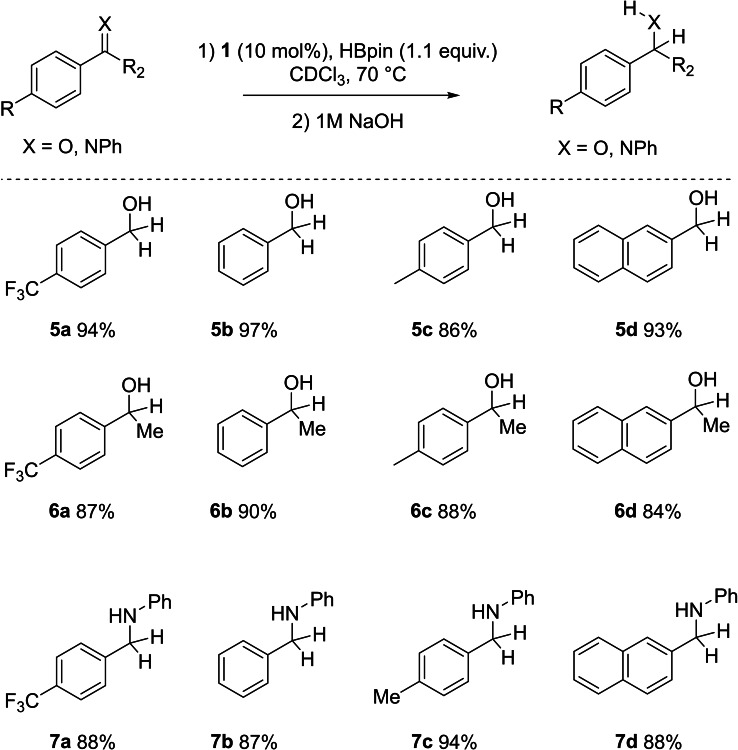
Hydroboration of aldehydes, ketones and imines utilizing 10 mol % **1** as a catalyst. The yields reported are isolated yields.

Pursuing the optimal conditions outlined above (Table 2, entry 8), we endeavoured to broaden the substrate scope to gauge the aptitude of **1** as a hydroboration catalyst (Figure 4).

**Table 2:** Optimisation of catalytic hydroboration.

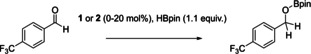					
entry	Catalyst loading (mol %)	Solvent	T (°C)	Yield^[a]^	Time (h)
**1**	0	CDCl_3_	25	<5 %	24
**2**	5	CDCl_3_	25	30 %	24
**3**	10	CDCl_3_	25	34 %	24
**4**	20	CDCl_3_	25	38 %	24
**5**	10	C_6_D_6_	25	39 %	24
**6**	10	d^8^‐THF	25	44 %	24
**7**	10	d^8^‐Tol	25	21 %	24
**8**	10	CDCl_3_	70	>95 %	3
**9** ^[b]^	10	CDCl_3_	70	<5 %	3
**10** ^[c]^	10	CDCl_3_	70	42 %	3

^[a]^ Conversion monitored by ^1^H NMR spectroscopy with mesitylene as an internal standard (0.1 mmol). ^[b]^ **2** as catalyst, ^[c]^ 0.1 mmol of TMEDA included.^[33]^

Initially, aldehydes underwent facile reduction within 1–3 hours, yielding alcohols (**5 a–d**) in excellent isolated yields of up to 97 % upon hydrolysis workup and purification. The catalyst displayed little preferential discrimination between substrates possessing electron‐withdrawing, electron‐donating, or sterically demanding groups. In a similar vein, ketones and aldimines underwent efficient reduction, culminating in high isolated yields (**6 a–6 d**, **7 a–7 d**).

## Conclusions

In conclusion, we have successfully synthesized two bench stable 1,4‐azaborinine derivatives based on anthracene/pentacene moieties. Structural analysis revealed that the incorporation of a N‐benzyl group fostered weak intermolecular CH‐π interactions for compound **2**, consequently enabling the molecules to stack in sheets. In the case of the pentacene derived, ladder‐type molecule **2**, red‐shifted absorption maxima and fluorescence were observed, accompanied by a detrimental decrease in quantum yields. Moreover, the Lewis acidity of compound **2** was found to be lower than that of **1**. Importantly, azaborinine **1** proved to be an effective catalyst for hydroboration reduction, exhibiting broad tolerance towards carbonyls and imines. These findings underscore the potential applicability of B,N π‐conjugated molecules in catalytic processes and open up avenues for further exploration in this field.

## Experimental Section

**General Experimental**: All reactions were carried out under a N_2_ using standard glovebox and Schlenk techniques.^1^H, ^13^C, ^11^B and ^19^F NMR spectra were recorded on a Bruker Ascend 400 MHz NMR spectrometer. ^1^H and ^13^C signals appear downfield and are referenced to tetramethylsilane (TMS) (0/0 ppm) as an internal standard. ^11^B are referenced to BF_3_⋅Et_2_O/CDCl_3_. Yields are given as isolated yields. HRMS samples were analysed on a LTQ Orbitrap XL 2. Crystal data were collected on a Bruker D8 Quest ECO diffractometer using graphite‐monochromated Mo Kα radiation and a Photon II−C14 CPAD detector. Fluorescence and UV‐Vis spectra were collected using a Varian Cary Eclipse Fluorescence Spectrophotometer and a single beam Varian Cary 50 Bio UV‐Visible spectrophotometer respectively. Relative quantum yields were calculated using quinine in 0.5 M H_2_SO_4_.^[38]^ All electrochemical experiments were conducted on a Biologic SP‐150e potentiostat using ferrocene as an internal standard.

**Materials**: All reaction solvents were dried, distilled and degassed using standard techniques. Deuterated solvents were distilled and/or dried over molecular sieves before use. Chemicals were purchased from commercial suppliers and used as received except a) bis(2‐bromophenyl)amine,^[39]^ b) 1,5‐dibromo‐2,4‐diiodobenzene^[40]^ and c) the imine starting materials^[41]^ which were synthesised according to literature protocols.

**Synthesis of 3**: Adapted from a reported procedure.^[42]^ Sodium hydride (0.103 g, 2.69 mmol) was suspended in a 1 : 1 mixture of THF and 1,4‐dioxane (20 mL) before bis(2‐bromophenyl)amine (0.732 g, 2.24 mmol) and benzylbromide (0.40 mL, 3.34 mmol) were added. The reaction was heated to reflux for 65 hours. Upon cooling all volatiles were removed in vacuo. The residue was dissolved in chloroform (50 mL) and water (50 mL). The organic layer was separated, washed with aqueous sodium carbonate (50 mL), dried over sodium sulphate and the volatiles removed in vacuo. The product was purified by column chromatography (eluent: petroleum ether) to give **3** as a white crystalline solid. White crystals suitable for X‐ray diffraction were obtained by recrystallisation from petroleum ether. Yield: 2.17 g, 5.20 mmol, 67 %. ^**1**^ **H NMR** (400 MHz; CDCl_3_, Me_4_Si, 295 K) *δ*/ppm: 7.57 (dd, 2H, J=7.8, 1.5 Hz), 7.50 (d, 2H, J=7.5 Hz), 7.25–7.23 (m, 2H), 7.18–7.12 (m,3H), 6.94–6.88 (m, 4H), 4.83 (s, 2H). ^**13**^ **C{^1^H} NMR** (101 MHz; CDCl_3_, Me_4_Si, 295 K) *δ*/ppm: 147.0, 137.7, 134.3, 128.3, 127.8, 127.5, 126.9, 125.4, 125.0, 121.3, 56.7. **HRMS** (APCI) calculated C_19_H_14_Br_2_N ([M+H]^+^) 413.9488. Found 413.9481.

**Synthesis of azaborinine 1**: Adapted from reported procedure.^[30]^ ^n^BuLi (5.0 mL, 8.05 mmol, 1.6 M in hexane) was added to **3** (1.53 g, 3.66 mmol) in Et_2_O (40 mL) at −78 °C, and the mixture was stirred for 30 minutes at 0 °C. Dimethyl(mesityl)boronate (2.35 mL, 4.39 mmol) was added, and the mixture was heated to reflux for 4 days. The resulting crude product was filtered through celite, the volatiles removed in vacuo and purified by column chromatography (Al_2_O_3_, 99 : 1 hexane/EtOAc) and recrystallised from hexane to afford **1** as white coloured crystals. Yield: 1.22 g, 3.15 mmol 86 %. ^**1**^ **H NMR** (400 MHz; CDCl_3_, Me_4_Si, 295 K) *δ*/ppm: 7.91 (dd, 2H, J=7.6, 1.7 Hz), 7.67 (ddd, 2H, J=8.7, 7.0, 1.7 Hz), 7.5–7.3 (m, 7H), 7.17 (ddd, 2H J=7.6, 7.0, 0.66 Hz), 7.00 (s, 2H), 5.81 (s, 2H), 2.44 (s, 3H), 2.04 (s, 6H). ^**13**^ **C{^1^H} NMR** (101 MHz; CDCl_3_, Me_4_Si, 295 K) *δ*/ppm: 146.3, 139.3, 137.5, 136.7, 136.4, 133.6, 129.1, 127.4, 126.8, 125.9, 119.9, 115.3, 52.7 23.3, 21.3. ^**11**^ **B{^1^H} NMR** (128.34 MHz; CDCl_3_, BF_3_.OEt_2_, 295 K) *δ*/ppm: 58.46. **HRMS** (APCI) calculated C_19_H_14_Br_2_N ([M]^+^) 386.2189. Found 386.2192.

**Synthesis of 4**: Adapted from reported procedure.^[42]^ Sodium hydride (0.138 g, 3.60 mmol) was suspended in a 1 : 1 mixture of THF and 1,4‐dioxane (40 mL) before 2,4‐dibromo‐1,5‐bis(2‐bromophenylamine)‐benzene (0.866 g, 1.50 mmol) and benzylbromide (0.93 mL, 3.75 mmol) were added. The reaction was heated to reflux for 48 hours and then cooled. The solvent was removed in vacuo, and the residue was dissolved in chloroform (50 mL) and water (50 mL). The organic layer was separated and washed with aqueous sodium carbonate (50 mL), then dried over sodium sulphate and the solvent was removed in vacuo. The product was purified by column chromatography (eluent: cyclohexane) to give **4** as off‐white crystals. White coloured crystals suitable for X‐ray diffraction were obtained by recrystallisation in cyclohexane. Yield: 0.894 g, 1.18 mmol, 79 %. ^**1**^ **H NMR** (400 MHz; CDCl_3_, Me_4_Si, 295 K) *δ*/ppm: 7.63 (s, 1H), 7.50 (dd, 2H, J=7.9, 1.5 Hz), 7.33 (dd, 4H, J=7.4, 1.2 Hz), 7.25–7.19 (m, 6H), 7.08 (ddd, 2H, J=7.9, 7.4, 1.5 Hz), 6.92 (ddd, 2H, J=7.9, 7.4, 1.5 Hz), 6.63 (s, 1H), 6.60 (dd, 2H, J=8.0, 1.5 Hz), 4.60 (s, 4H). ^**13**^ **C{^1^H} NMR** (101 MHz; CDCl_3_, Me_4_Si, 295 K) *δ*/ppm: 146.6, 145.8, 138.2, 137.3, 134.4, 128.4, 127.9, 127.6, 127.0, 125.7, 125.4, 121.9, 121.5, 115.1, 56.9. **HRMS** (APCI) calculated C_32_H_25_Br_4_N_2_ ([M+H]^+^) 752.8746. Found 752.8753.

**Synthesis of azaborinine 2**: ^n^BuLi (3.7 mL, 6.68 mmol, 1.6 M in hexane) was added to **4** (1.10 g, 1.34 mmol) in Et_2_O (30 mL) at −75 °C, the mixture was stirred for 30 minutes at 0 °C. Dimethyl(mesityl)boronate (1.0 mL, 3.20 mmol) was added, and the mixture was heated to reflux for 4 days. The resulting mixture was filtered through Celite, washed with DCM and the volatiles removed *in vacuo*. The crude product was purified by column chromatography (Al_2_O_3_, 99 : 1 hexane/EtOAc) and recrystallised (layering, toluene: hexane, −20 °C) to afford **2** as yellow crystals. Yield: 120 mg, 0.172 mmol 13 %). ^**1**^ **H NMR** (400 MHz; CDCl_3_, Me_4_Si, 295 K) *δ*/ppm: 8.19 (s, 1H), 7.90 (dd, *J*=7.5, 1.8 Hz, 2H), 7.61 (ddd, *J*=8.8, 7.0, 1.8 Hz, 2H), 7.40 (d, *J*=8.8 Hz, 2H), 7.29 (m, 6H), 7.17–7.06 (m, 8H), 6.80 (s, 4H), 5.51 (br, 4H), 2.35 (s, 6H), 1.92 (s, 12H). ^**13**^ **C{^1^H} NMR** (101 MHz; CDCl_3_, Me_4_Si, 295 K) *δ*/ppm: 150.4, 149.0, 146.2, 138.0, 137.8, 136.7, 135.2, 135.0, 132.5, 127.9, 126.3, 126.1, 125.40, 124.9, 120.4, 119.2, 114.1, 97.9, 52.3, 22.2, 20.2 **HRMS** (APCI) calculated C_52_H_47_N2^10^B^11^B ([M+H]^+^) 696.3956. Found 696.3953.

**General Procedure for catalytic hydroborations**: In an NMR tube, pinacol borane (32 μL, 220 μmol, 1.1 equiv.) and the substrate (200 μmol, 1.0 equiv.) were combined in deuterated chloroform (0.7 mL). To this, azaborinine **1** (10 mg, 10 mol %, 20 μmol, 0.1 equiv.) was added, and the NMR tube sealed. The combined mixture was then heated to 70 °C. After the reaction was complete, the product was hydrolysed, using 1 M NaOH (3×10 mL). The crude mixture was extracted with ethyl acetate (3×10 mL). and dried (MgSO_4_).

**Purification**: Primary and secondary alcohols (**5–6**): The crude products were purified using preparatory TLC (hexane/ethyl acetate 5 : 1). Secondary amines (**7**): The crude product was dissolved in diethyl ether (5 mL). Ethereal HCl was added dropwise until solid precipitation ceased. The solid was filtered, washed with diethyl ether (3×5 mL) and suspended in 1 : 1 water/diethyl ether. 1 M NaOH was added dropwise until no solids remained. The organic phase was separated, washed (2×5 mL aq. NaCl) and dried (MgSO_4_).

Deposition Numbers 2267646 (for 1), 2267647 (for 2), 2267648 (for 3), 2267649 (for 4), contain the supplementary crystallographic data for this paper. These data are provided free of charge by the joint Cambridge Crystallographic Data Centre and Fachinformationszentrum Karlsruhe Access Structures service.

## Supporting Information[^43^, ^44^, ^45^, ^46^, ^47^, ^48^, ^49^, ^50^, ^51^, ^52^, ^53^, ^54^, ^55^, ^56^, ^57^, ^58^]

Supporting Information is available from the Wiley Online Library or from the author.

## Conflict of interest

The authors declare no conflict of interest.

1

## Supporting information

As a service to our authors and readers, this journal provides supporting information supplied by the authors. Such materials are peer reviewed and may be re‐organized for online delivery, but are not copy‐edited or typeset. Technical support issues arising from supporting information (other than missing files) should be addressed to the authors.

Supporting Information

## Data Availability

The data that support the findings of this study are available from the corresponding author upon reasonable request.
