# Supplementary material for: Juggling Optoelectronics and Catalysis: The Dual Talents of Bench Stable 1,4‐Azaborinines
Source: Chemistry. 2023 Dec 22;30(8):e202301944. doi: 10.1002/chem.202301944 (PMC11497314; doi:10.1002/chem.202301944)
Supplement: Supplementary file 1 — Supporting Information [file CHEM-30-e202301944-s001.pdf]

Chemistry–A European Journal

Supporting Information

Juggling Optoelectronics and Catalysis: The Dual Talents of Bench Stable 1,4-Azaborinines

Chloe M. van Beek, Amelia M. Swarbrook, Charles E. Creissen, Chris S. Hawes, Theodore A. Gazis,* and Peter D. Matthews*

Table of Contents

1 Experimental 2

1.1 General experimental 2

1.2 Synthesis of azaborinines 3

2. NMR Spectra 10

2.1 NMR of azaborinines 10

2.2 NMR of reduction products 22

3 Crystallographic Data 46

3.1 X-ray refinement data 47

4 Cyclic Voltammetry 53

5 Computational Analysis 54

6 References 59

1 Experimental

1.1 General experimental

All reactions and manipulations were carried out under an atmosphere of dry, O_2-free nitrogen using standard double-manifold techniques with a rotary oil pump. A nitrogen-filled glove box (MBraun) was used to manipulate solids including the storage of starting materials, product recovery and sample preparation for analysis. All solvents were dried and distilled using standard techniques. They were degassed prior to use. Deuterated solvents were distilled and/or dried over molecular sieves before use. Chemicals were purchased from commercial suppliers and used as received. Bis(2-bromophenyl)amine[1] and 1,5-dibromo-2,4-diiodobenzene[2] were synthesised according to the literature procedure.

The imine starting materials were synthesised according to a literature known procedure.[3]

^{1}H, ^{13}C, ^{11}B and ^{19}F NMR spectra were recorded on a Bruker Ascend 400 MHz NMR spectrometer. Chemical shifts are expressed as parts per million (ppm, δ). ^{1}H and ^{13}C signals appear downfield and are referenced to tetramethylsilane (TMS) (0/0 ppm) as an internal standard. ^{11}B are referenced to $BF_3{\cdot}Et_2O/CDCl_3$. The description of signals includes s = singlet, d = doublet, t = triplet, q = quartet, m = multiplet and br. = broad. All coupling constants are absolute values and are expressed in Hertz (Hz). Yields are given as isolated yields.

Fluorescence and UV-Vis spectra were collected using a Varian Cary Eclipse Fluorescence Spectrophotometer and a single beam Varian Cary 50 Bio UV-Visible spectrophotometer respectively. Relative quantum yields were calculated using quinine in 0.5M H_2SO_4.[4]

HRMS samples were analysed on a LTQ OrbitrapxL 2

1.2 Synthesis of azaborinines

Scheme S1

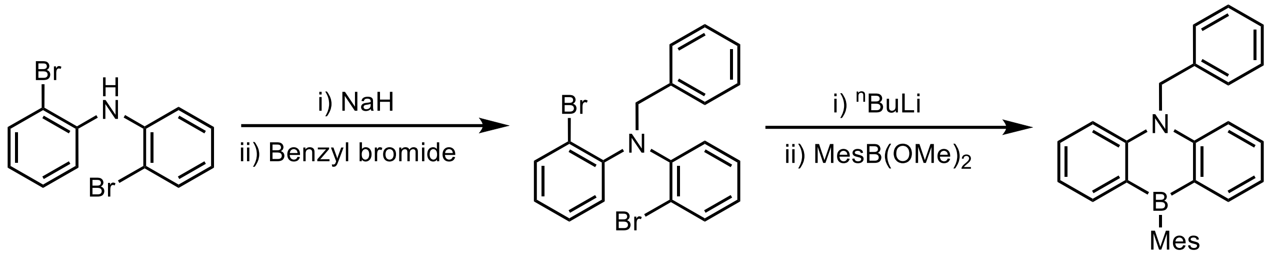

3 **1**

Synthesis of *N-benzyl-bis(2-bromophenyl)amine* (**3**)

Adapted from a reported procedure.[5] Sodium hydride (0.103 g, 2.69 mmol) was suspended in a 1:1 mixture of THF and 1,4-dioxane (20 mL) before bis(2-bromophenyl)amine (0.732 g, 2.24 mmol) and benzylbromide (0.40 mL, 3.34 mmol) were added. The reaction was heated to refluxfor 65 hours. Upon cooling all volatiles were removed in vacuo. The residue was dissolved in chloroform (50 mL) and water (50 mL). The organic layer was separated, washed with aqueous sodium carbonate (50 mL), dried over sodium sulfate and the volatiles removed in vacuo. The product was purified by column chromatography (eluent: petroleum ether) to give **3** as a white crystalline solid. White crystals suitable forx-ray diffraction were obtained by recrystallisation from petroleum ether. Yield: 2.17 g, 5.20 mmol, 67%. **^{1}H NMR** (400 MHz; $CDCl_3$, Me_4Si, 295K) *δ*/ppm: 7.57 (dd, 2H, J = 7.8, 1.5 Hz), 7.50 (d, 2H, J = 7.5 Hz), 7.25–7.23 (m, 2H), 7.18–7.12 (m,3H), 6.94–6.88 (m, 4H), 4.83 (s, 2H). **^{13}C{^{1}H} NMR** (101 MHz; $CDCl_3$, Me_4Si, 295K) *δ*/ppm: 147.0, 137.7, 134.3, 128.3, 127.8, 127.5, 126.9, 125.4, 125.0, 121.3, 56.7. **HRMS** (APCI) calculated $C_{19}H_{14}Br_2N$ ($[M+H]^+$) 413.9488. Found 413.9481.

Synthesis of *B-(2,4,6-Trimethylphenyl)-N-benzyl-5,10-dihydro-dibenzo-1,4-azaborine* (**1**)

Adapted from reported procedure.[6] ^{n}BuLi (5.0 mL, 8.05 mmol, 1.6 M in hexane) was added to **3** (1.53 g, 3.66 mmol) in Et_2O (40 mL) at–78 °C, and the mixture was stirred for 30 minutes at 0 °C. Dimethyl(mesityl)boronate (2.35 mL, 4.39 mmol) was added, and the mixture was heated to refluxfor 4 days. The resulting crude product was filtered through celite, the volatiles removed in vacuo.and purified by column chromatography (Al_2O_3, 99:1 hexane/EtOAc) and recrystallised from hexane to afford **1** as white coloured crystals. Yield: 1.22 g, 3.15 mmol 86%. **^{1}H NMR** (400 MHz; $CDCl_3$, Me_4Si, 295K) *δ*/ppm: 7.91 (dd, 2H, J = 7.6, 1.7 Hz), 7.67 (ddd, 2H, J = 8.7, 7.0, 1.7 Hz), 7.5–7.3 (m, 7H), 7.17 (ddd, 2H J = 7.6, 7.0, 0.66 Hz), 7.00 (s, 2H), 5.81 (s, 2H), 2.44 (s, 3H), 2.04 (s, 6H). **^{13}C{^{1}H} NMR** (101 MHz; $CDCl_3$, Me_4Si, 295K) *δ*/ppm: 146.3, 139.3, 137.5, 136.7, 136.4, 133.6, 129.1, 127.4, 126.8, 125.9, 119.9, 115.3, 52.7 23.3, 21.3. **^{11}B{^{1}H} NMR** (128.34 MHz; $CDCl_3$, $BF_3.OEt_2$, 295K) *δ*/ppm: 58.46. **HRMS** (APCI) calculated $C_{19}H_{14}Br_2N$ ($[M]^+$) 386.2189. Found 386.2192.

Scheme S2

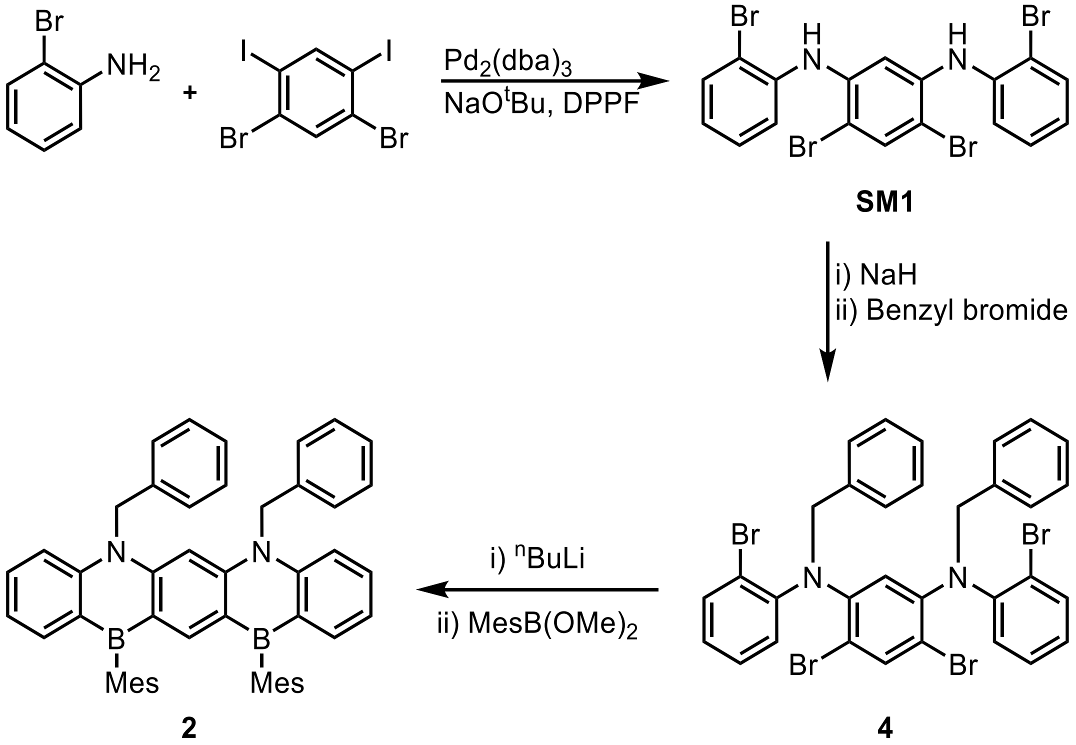

Synthesis of *2,4-dibromo-1,5-bis(2-bromophenylamine)-benzene* (**SM1**)

2-bromoaniline (3.78 g, 22.0 mmol), NaO^{t}Bu (2.31 g, 24.0 mmol), $Pd_2(dba)_3$ (458 mg, 0.50 mmol), DPPF (555 mg, 1.00 mmol), 1,5-dibromo-2,4-diiodobenzene (4.88 g, 10.0 mmol) and toluene (20 mL) were stirred at 120 °C for 4 days. The volatiles were removed *in vacuo*, sat. NH_4Cl (50 mL) was added, and the solution was extracted with EtOAc (50 mL) then dried over Na_2SO_4. The crude product was dry loaded onto silica. The crude product was purified using column chromatography (eluent: petroleum ether, RF = 0.48) to give **SM1** as a cream coloured solid (1.21 g, 2.09 mmol, 21 % yield). **^{1}H NMR** (400 MHz; $CDCl_3$; Me_4Si, 295K) δ/ppm: 7.73 (1H, s), 7.55 (2H, dd, J = 8.0, 1.3 Hz), 7.23 (2H, dd, J =8.1, 1.3 Hz), 7.19 (2H, td, J = 8.1, 1.3 Hz), 7.08 (1H, s), 6.84 (2H, ddd, J = 8.0, 7.1, 1.8 Hz), 6.35 (2H, s). **^{13}C{^{1}H} NMR** (101 MHz; $CDCl_3$; Me_4Si, 295K) δ/ppm: 140.2, 139.4, 135.9, 133.4, 128.1, 123.3, 118.9, 115.0, 105.2, 104.5. **HRMS** (APCI) calculated $C_{18}H_{13}Br_4N_2$ ($[M+H]^+$) 572.7807. Found 572.7813.

Synthesis of *2,4-dibromo-1,5-N-dibenzyl-N,N-bis(2-bromophenylamine)-benzene* (**4**)

Adapted from reported procedure.[2] Sodium hydride (0.138 g, 3.60 mmol) was suspended in a 1:1 mixture of THF and 1,4-dioxane (40 mL) before 2,4-dibromo-1,5-bis(2-bromophenylamine)-benzene (0.866 g, 1.50 mmol) and benzylbromide (0.93 mL, 3.75 mmol) were added. The reaction was heated to refluxfor 48 hours and then cooled. The solvent was removed in vacuo, and the residue was dissolved in chloroform (50 mL) and water (50 mL). The organic layer was separated and washed

with aqueous sodium carbonate (50 mL), then dried over sodium sulfate and the solvent was removed in vacuo. The product was purified by column chromatography (eluent: cyclohexane) to give **4** as off-white crystals. White coloured crystals suitable for X-ray diffraction were obtained by recrystallisation in cyclohexane. Yield: 0.894 g, 1.18 mmol, 79%. **^{1}H NMR** (400 MHz; $CDCl_3$, Me_4Si, 295K) *δ*/ppm: 7.63 (s, 1H), 7.50 (dd, 2H, J = 7.9, 1.5 Hz), 7.33 (dd, 4H, J = 7.4, 1.2 Hz), 7.25–7.19 (m, 6H), 7.08 (ddd, 2H, J = 7.9, 7.4, 1.5 Hz), 6.92 (ddd, 2H, J = 7.9, 7.4, 1.5 Hz), 6.63 (s, 1H), 6.60 (dd, 2H, J = 8.0, 1.5 Hz), 4.60 (s, 4H). **^{13}C{^{1}H} NMR** (101 MHz; $CDCl_3$, Me_4Si, 295K) *δ*/ppm: 146.6, 145.8, 138.2, 137.3, 134.4, 128.4, 127.9, 127.6, 127.0, 125.7, 125.4, 121.9, 121.5, 115.1, 56.9. **HRMS** (APCI) calculated $C_{32}H_{25}Br_4N_2$ ($[M+H]^+$) 752.8746. Found 752.8753.

Synthesis of *5,7-dimesityl-12,14-dibenzyl-5,7,12,14-tetrahydro-5,7-dibora-12,14-diaza-pentacene* (**2**)
^{n}BuLi (3.7 mL, 6.68 mmol, 1.6 M in hexane) was added to **4** (1.10 g, 1.34 mmol) in Et_2O (30 mL) at–75 °C, the mixture was stirred for 30 minutes at 0 °C. Dimethyl(mesityl)boronate (1.0 mL, 3.20 mmol) was added, and the mixture was heated to refluxfor 4 days. The resulting mixture was filtered through Celite, washed with DCM and the volatiles removed in vacuo. The crude product was purified by column chromatography (Al_2O_3, 98:2 hexane/EtOAc) and recrystallised (layering, toluene: hexane, -20 °C) to afford **2** as yellow crystals. Yield: 120 mg, 0.172 mmol 13%). **^{1}H NMR** (400 MHz; $CDCl_3$, Me_4Si, 295K) *δ*/ppm: 8.19 (s, 1H), 7.90 (dd, *J* = 7.5, 1.8 Hz, 2H), 7.61 (ddd, *J* = 8.8, 7.0, 1.8 Hz, 2H), 7.40 (d, *J* = 8.8 Hz, 2H), 7.29 (m, 6H), 7.17 – 7.06 (m, 8H), 6.80 (s, 4H), 5.51 (br, 4H), 2.35 (s, 6H), 1.92 (s, 12H). **^{13}C{^{1}H} NMR** (101 MHz; $CDCl_3$, Me_4Si, 295K) *δ*/ppm: 150.4, 149.0, 146.2, 138.0, 137.8, 136.7, 135.2, 135.0, 132.5, 127.9, 126.3, 126.1, 125.40, 124.9, 120.4, 119.2, 114.1, 97.9, 52.3, 22.2, 20.2 **HRMS** (APCI) calculated $C_{52}H_{47}N2^{10}B^{11}B$ ($[M+H]^+$) 696.3956. Found 696.3953.

1.3 Synthesis of reduction products

General Procedure:

Synthesis

In an NMR tube, pinacol borane (32 µL, 220 µmol, 1.1 equiv.) and the substrate (200 µmol, 1.0 equiv.) were combined in deuterated chloroform (0.7 mL). To this, azaborinine **1** (10 mg, 10 mol%, 20 µmol, 0.1 equiv.) was added, and the NMR tube sealed. The combined mixture was then heated to 70 °C, with conversion tracked by *in situ* ^{1}H NMR spectroscopy until the targeted boronate ester was produced in >95% yield. After the reaction was complete, the boronate ester was hydrolysed, using 1M NaOH (3x10 mL). The crude mixture was extracted with ethyl acetate (3x10 mL). and dried ($MgSO_4$).

Purification

Primary and secondary alcohols (**5–6**): The crude products were purified using preparatory TLC (hexane/ethyl acetate 5:1).

Secondary amines (**7**): The crude product was dissolved in diethyl ether (5 mL). Ethereal HCl was added dropwise until solid precipitation ceased. The solid was filtered, washed with diethyl ether (3x5 mL) and suspended in 1:1 water/diethyl ether. 1M NaOH was added dropwise until no solids remained. The organic phase was separated, washed (2x5 mL aq. NaCl) and dried ($MgSO_4$).

Synthesis of *(4-(trifluoromethyl)phenyl)methanol* (**5a**)

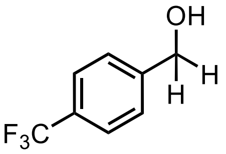

Synthesized in accordance with general procedure using 4-(trifluoromethyl)benzaldehyde (27 µL, 200 µmol) to afford the title compound as an off-white oil. Yield: 33 mg, 187 µmol, 94%. Spectroscopic analyses agree with literature values.[7] **^{1}H NMR** (400 MHz, $CDCl_3$, Me_4Si, 295K) *δ*/ppm: 7.62 (d, 2H), 7.48 (d, 2H), 4.78 (s, 2H), 1.82 (br, 1H). **^{13}C{^{1}H} NMR** (101 MHz; $CDCl_3$, Me_4Si, 295K) *δ*/ppm: 144.7, 129.8 (q), 128.2, 126.8, 125.5 (q), 122.8, 120.1, 64.5.

Synthesis of *phenylmethanol* (**5b**)

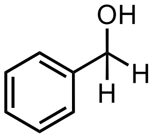

Synthesized in accordance with general procedure using benzaldehyde (21 µL, 200 µmol) to afford the title compound as a colourless oil. Yield: 21 mg, 193 µmol, 97%. Spectroscopic analyses agree with literature values.[3] **^{1}H NMR** (400 MHz, $CDCl_3$, Me_4Si, 295K) *δ*/ppm: 7.35 (d, 2H), 7.33–7.27 (m, 1H), 4.7 (d, 2H), 1.66 (br, 1H). **^{13}C{^{1}H} NMR** (101 MHz; $CDCl_3$, Me_4Si, 295K) *δ*/ppm: 140.8, 128.6, 127.7, 127.0, 65.4.

Synthesis of *p-tolylmethanol* (**5c**)

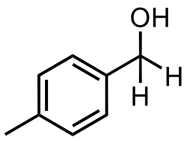

Synthesized in accordance with general procedure using 4-methylbenzaldehyde (19 μL, 200 μmol) to afford the title compound as a colourless oil. Yield: 21 mg, 172 μmol, 86%. Spectroscopic analyses agree with literature values.[7] **^{1}H NMR** (400 MHz, $CDCl_3$, Me_4Si, 295K) *δ*/ppm: 7.25 (d, 2H), 7.16 (d, 2H), 4.63 (s, 2H), 2.35 (s, 3H), 1.72 (s, 1H). **^{13}C{^{1}H} NMR** (101 MHz; $CDCl_3$, Me_4Si, 295K) *δ*/ppm: 137.9, 137.4, 129.2, 127.1, 65.2, 21.1.

Synthesis of *napthalen-2-ylmethanol* (**5d**)

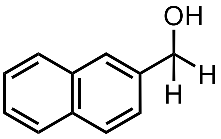

Synthesized in accordance with general procedure using 2-napthaldehyde (31 mg, 200 μmol) to afford the title compound as a white solid. Yield: 32 mg, 186 μmol, 93%. Spectroscopic analyses agree with literature values.[3] **^{1}H NMR** (400 MHz, $CDCl_3$, Me_4Si, 295K) *δ*/ppm: 7.84–7.79 (m, 4H), 7.48–7.44 (m, 3H), 4.84 (s, 2H), 1.85 (s, 1H). **^{13}C{^{1}H} NMR** (101 MHz; $CDCl_3$, Me_4Si, 295K) *δ*/ppm: 138.3, 133.4, 132.9, 128.3, 127.9, 127.7, 126.2, 125.9, 125.4, 125.1, 65.5.

Synthesis of *1-(4-(trifluoromethyl)phenyl)ethanol* (**6a**)

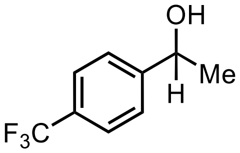

Synthesized in accordance with general procedure using 4-(trifluoromethyl)acetophenone (40 μL, 200 μmol) to afford the title compound as a colourless oil. Yield: 33 mg, 173 μmol, 87%. Spectroscopic analyses agree with literature values.[8] **^{1}H NMR** (400 MHz, $CDCl_3$, Me_4Si, 295K) *δ*/ppm: 7.61 (d, 2H), 7.49 (d, 2H), 4.97 (q, 1H), 1.51 (d, 3H). **^{13}C{^{1}H} NMR** (101 MHz; $CDCl_3$, Me_4Si, 295K) *δ*/ppm: 149.7, 129.8, 129.3 (d) 125.6,125.5(q), 122.8, 120.1, 69.8, 25.4.

Synthesis of *1-phenylethanol* (**6b**)

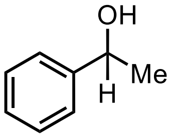

Synthesized in accordance with general procedure using acetophenone (17μL, 200 μmol) to afford the title compound as a colorless oil. Yield: 22 mg, 180 μmol, 90%. Spectroscopic analyses agree with literature values.[3] **^{1}H NMR** (400 MHz, $CDCl_3$, Me_4Si, 295K) *δ*/ppm: 7.40–7.33 (m, 4H), 7.30–7.27 (m, 1H), 4.91 (q, 1H), 1.79 (s, 1H), 1.50 (d, 3H). **^{13}C{^{1}H} NMR** (101 MHz; $CDCl_3$, Me_4Si, 295K) *δ*/ppm: 145.8, 128.5, 127.5, 125.4, 70.4, 25.2.

Synthesis of *1-(4-tolyl)ethanol* (**6c**)

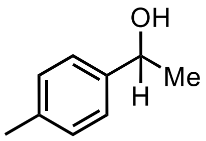

Synthesized in accordance with general procedure using 4-methylacetophenone (21 µL, 200 µmol) to afford the title compound as a colourless oil. Yield: 21 mg, 176 µmol, 88%. Spectroscopic analyses agree with literature values.[8] **^{1}H NMR** (400 MHz, $CDCl_3$, Me_4Si, 295K) *δ*/ppm: 7.28 (d, 2H), 7.16 (d, 2H), 4.87 (q, 1H), 2.34 (s, 3H), 1.75 (s, 1H), 1.48 (s,3H). **^{13}C{^{1}H} NMR** (101 MHz; $CDCl_3$, Me_4Si, 295K) *δ*/ppm: 142.9, 137.2, 129.2, 125.3, 70.3, 25.1, 21.1 .

Synthesis of napthalen-2-ylmethanol (**6d**)

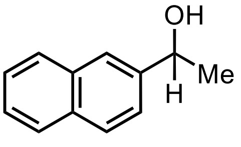

Synthesized in accordance with general procedure using 2-acetylnaphtalene (36 mg, 200 µmol) to afford the title compound as a colourless oil. Yield: 29 mg, 168 µmol, 84%. Spectroscopic analyses agree with literature values.[3] **^{1}H NMR** (400 MHz, $CDCl_3$, Me_4Si, 295K) *δ*/ppm: 7.83–7.78 (m, 4H), 7.50–7.43 (m, 3H), 5.04 (q, 1H), 2.03 (br, 1H), 1.56 (d, 3H). **^{13}C{^{1}H} NMR** (101 MHz; $CDCl_3$, Me_4Si, 295K) *δ*/ppm: 143.2, 133.3, 132.9, 128.3, 127.9, 127.7, 126.1, 125.8, 124.1 123.8, 123.8, 70.5, 25.1.

Synthesis of *N-(4-(trifluoromethyl)benzyl)aniline* (**7a**)

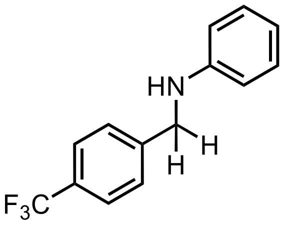

Synthesized in accordance with general procedure using 1-(4-trifluorophenyl)-N-phenylmethanimine (50 mg, 200 µmol) to afford the title compound as a yellow oil.[9] Yield: 44 mg, 175 µmol, 88%. Spectroscopic analyses agree with literature values.1 **^{1}H NMR** (400 MHz, $CDCl_3$, Me_4Si, 295K) *δ*/ppm: 7.51 (dd, 4H), 7.20–7.13 (m, 2H), 6.72 (tt, 1H), 6.59 (dd, 2H), 4.38 (s, 2H), 4.10 (br, 1H). **^{13}C{^{1}H} NMR** (101 MHz; $CDCl_3$, Me_4Si, 295K) *δ*/ppm: 147.7, 143.8, 129.3, 127.4, 125.6, 122.9, 120.9, 118.0, 112.9, 47.8.

Synthesis of *N-benzylaniline* (**7b**)

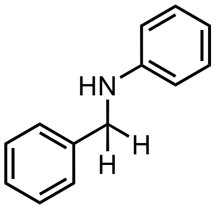

Synthesized in accordance with general procedure using N,1-diphenylmethanimine (37 mg, 200 µmol) to afford the title compound as a colourless oil. Yield: 32 mg, 175 µmol, 87%. Spectroscopic analyses agree with literature values.[3] **^{1}H NMR** (400 MHz, $CDCl_3$, Me_4Si, 295K) *δ*/ppm: 7.39–7.32 (m, 4H), 7.29–7.25 (m, 1H), 7.19–7.15 (m, 2H), 6.71–6.69 (t, 1H), 6.64 (dd, 2H), 4.33 (s, 2H), 4.02 (br, 1H). **^{13}C{^{1}H} NMR** (101 MHz; $CDCl_3$, Me_4Si, 295K) *δ*/ppm: 148.1, 139.4, 129.3, 128.6, 127.5, 127.2, 117.6, 112.8, 48.3.

Synthesis of *N-phenyl-1-(p-tolyl)methanimine* (**7c**)

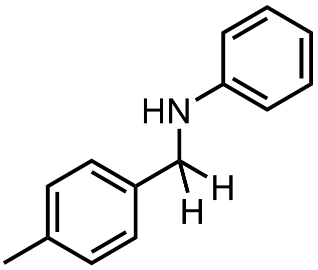

Synthesized in accordance with general procedure using 1-(4-methyl)-N-phenylmethanimine (39 mg, 200 µmol) to afford the title compound as an off-white solid. Yield: 37 mg, 187 µmol, 94%. Spectroscopic analyses agree with literature values.[9] **^{1}H NMR** (400 MHz, $CDCl_3$, Me_4Si, 295K) *δ*/ppm: 7.27–7.24 (m, 2H), 7.19–7.14 (m, 4H), 6.73–6.68 (m, 1H), 6.64–6.61 (m, 2H), 4.28 (s, 1H), 3.96 (s, br, 1H), 2.34 (s, 3H). **^{13}C{^{1}H} NMR** (101 MHz; $CDCl_3$, Me_4Si, 295K) *δ*/ppm: 148.2, 136.9, 136.3, 129.3, 129.2, 127.5, 117.5, 112.8, 48.1, 21.1.

Synthesis of *N-(naphthalen-2-ylmethyl)aniline* (**7d**)

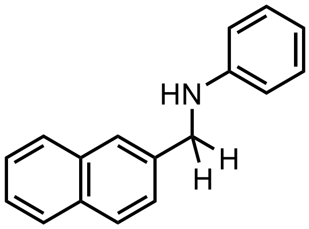

Synthesized in accordance with general procedure using 1-(naphthalen-2-yl)-N-phenylmethanimine (46 mg, 200 µmol) to afford the title compound as a colourless oil. Yield: 41 mg, 176 µmol, 88%. Spectroscopic analyses agree with literature values.[3] **^{1}H NMR** (400 MHz, $CDCl_3$, Me_4Si, 295K) *δ*/ppm: 7.83–7.79 (m, 4H) 7.50–7.43 (m, 3H) 7.19–7.15 (m, 2H), 6.74–6.66 (m, 3H) 4.49 (s, 2H), 4.13 (s, 1H). **^{13}C{^{1}H} NMR** (101 MHz; $CDCl_3$, Me_4Si, 295K) *δ*/ppm: 148.2, 136.9, 133.5, 132.8, 129.3, 128.4. 127.7, 127.6, 126.1, 125.9, 125.7, 117.6, 112.9, 48.5.

2. NMR Spectra

2.1 NMR of azaborinines

Figure S1 ^{1}H NMR (400 MHz, $CDCl_3$, Me_4Si, 295K) spectrum of **3**.

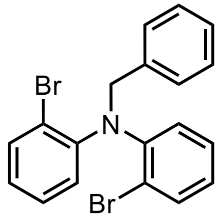

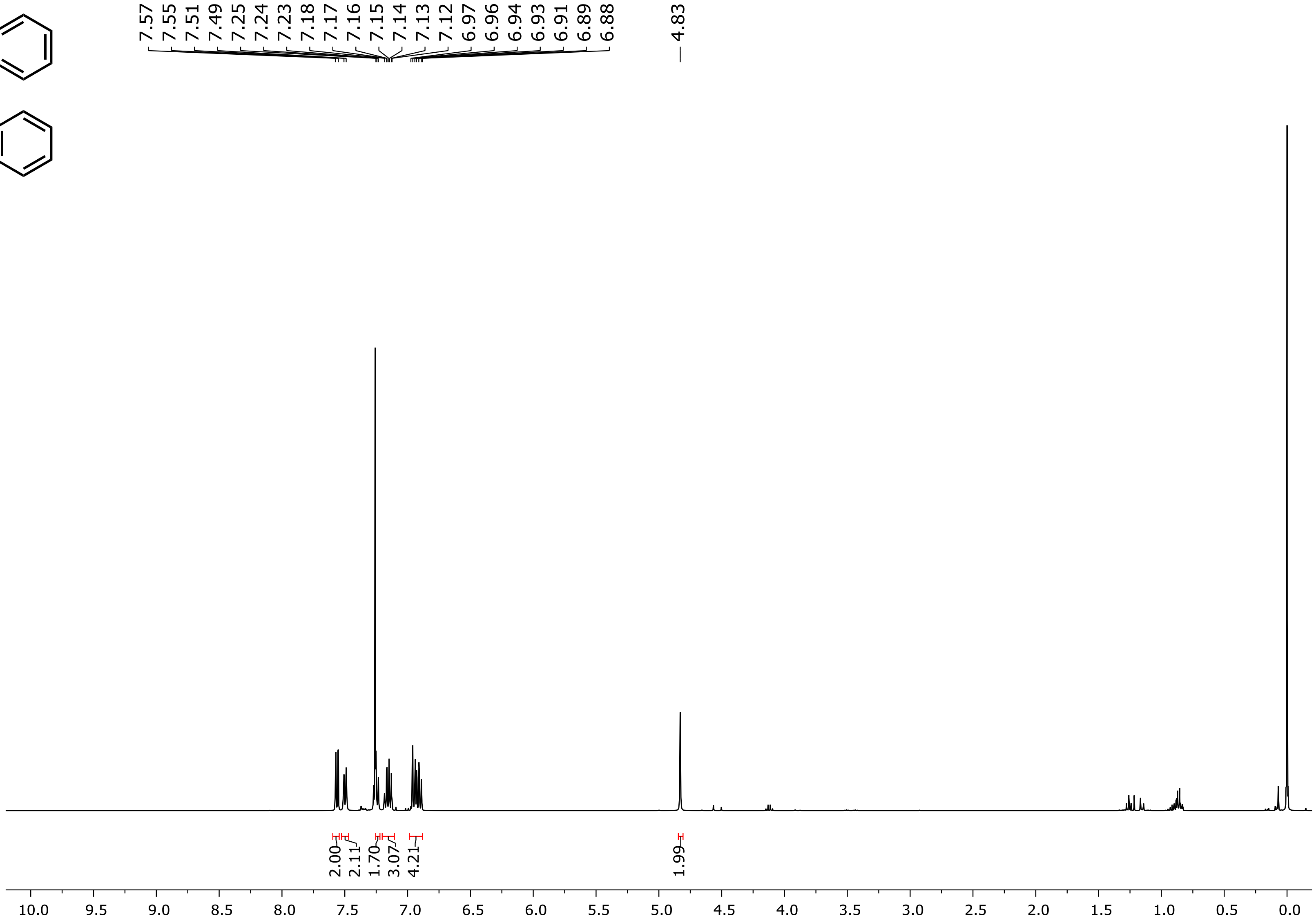

Figure S2 ^{13}C{^{1}H} NMR (101 MHz, $CDCl_3$, Me_4Si, 295K) spectrum of **3**.

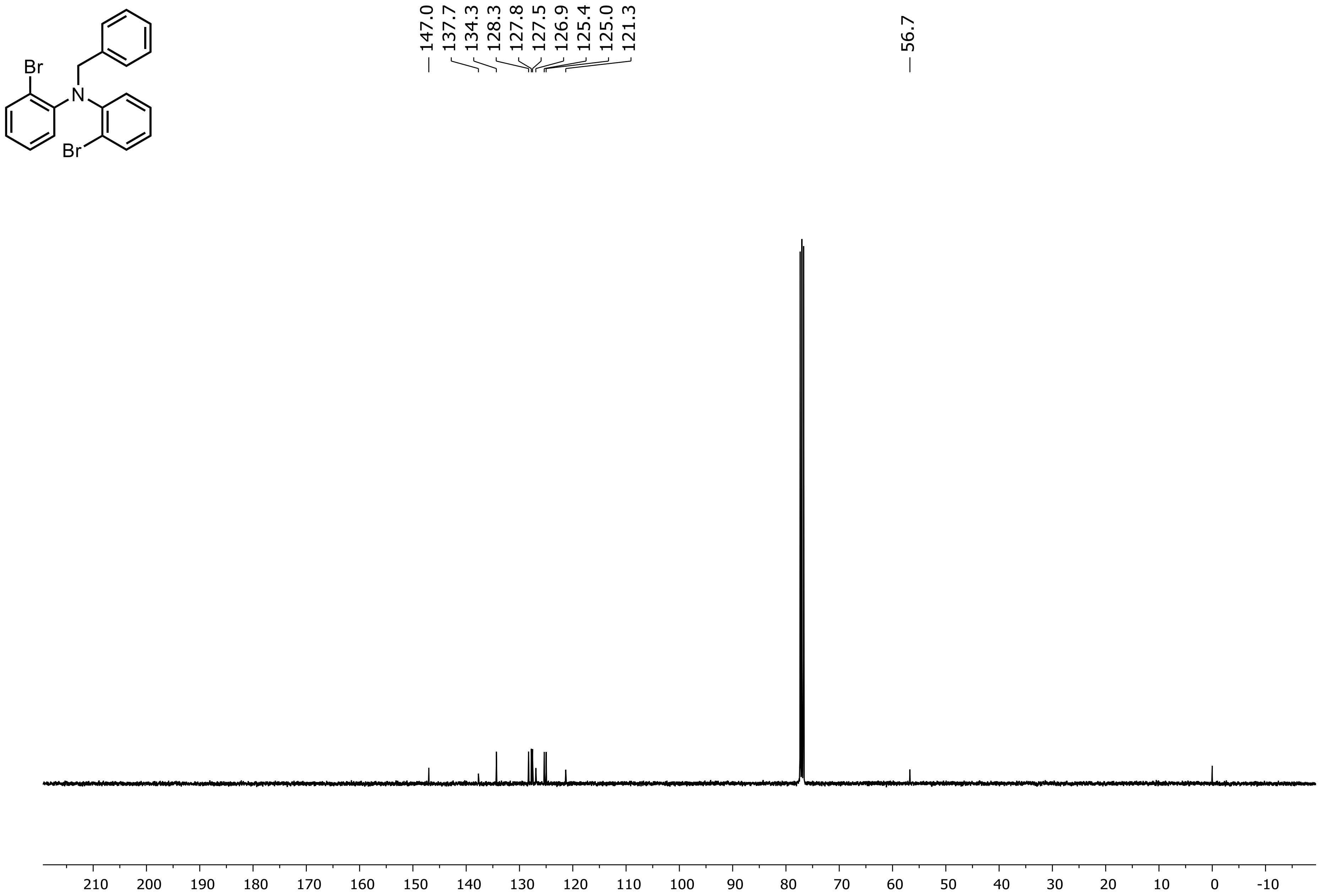

Figure S3 **^{1}H NMR** (400 MHz, $CDCl_3$, Me_4Si, 295K) spectrum of **1**.

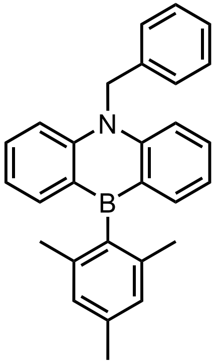

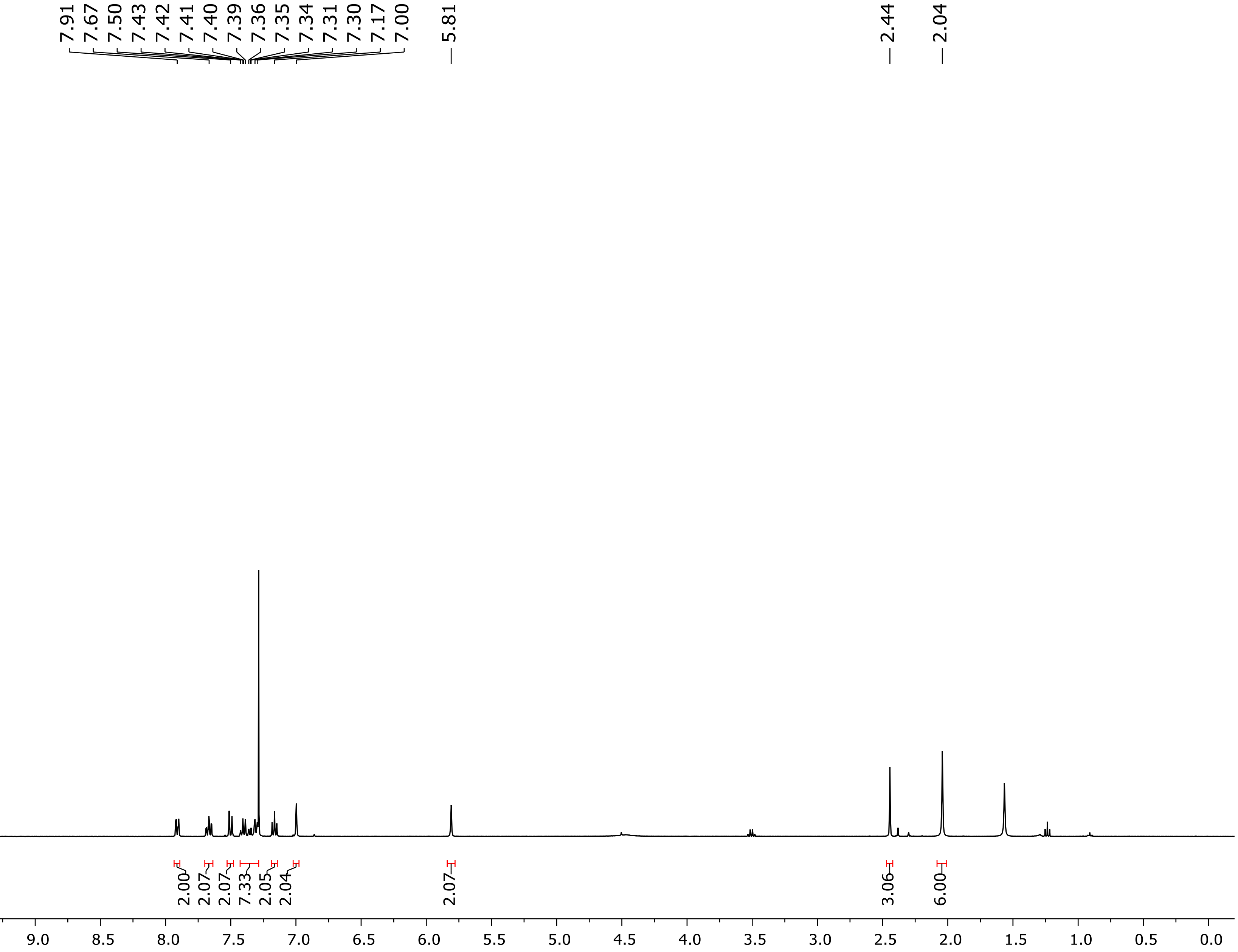

Figure S4 $^{13}C\{H\}$ NMR (101 MHz, $CDCl_3$, Me_4Si, 295K) spectrum of **1**.

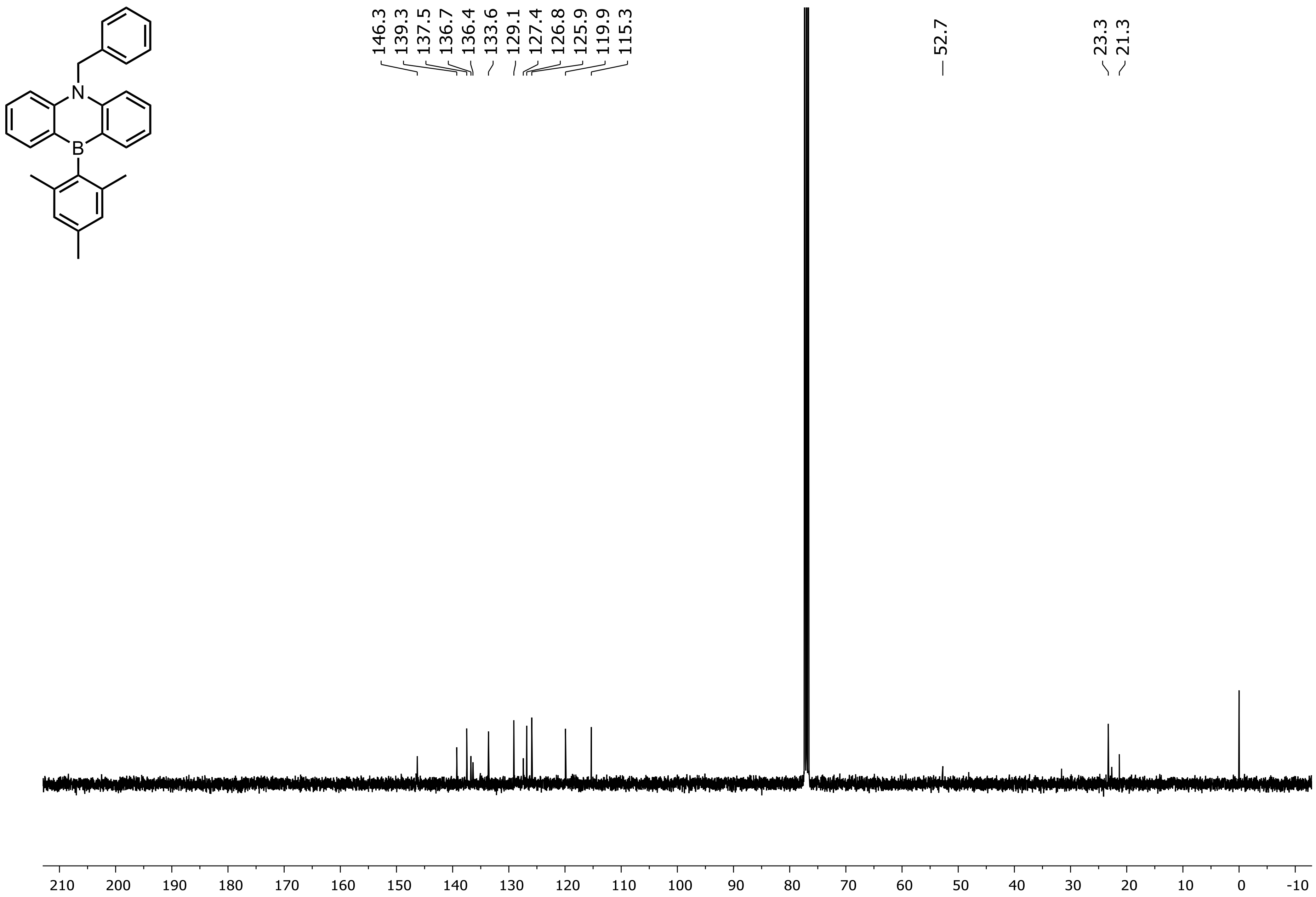

Figure S5 ^{11}B NMR (376 MHz, $CDCl_3$, BF_3Et_2O, 295K) spectrum of **1**.

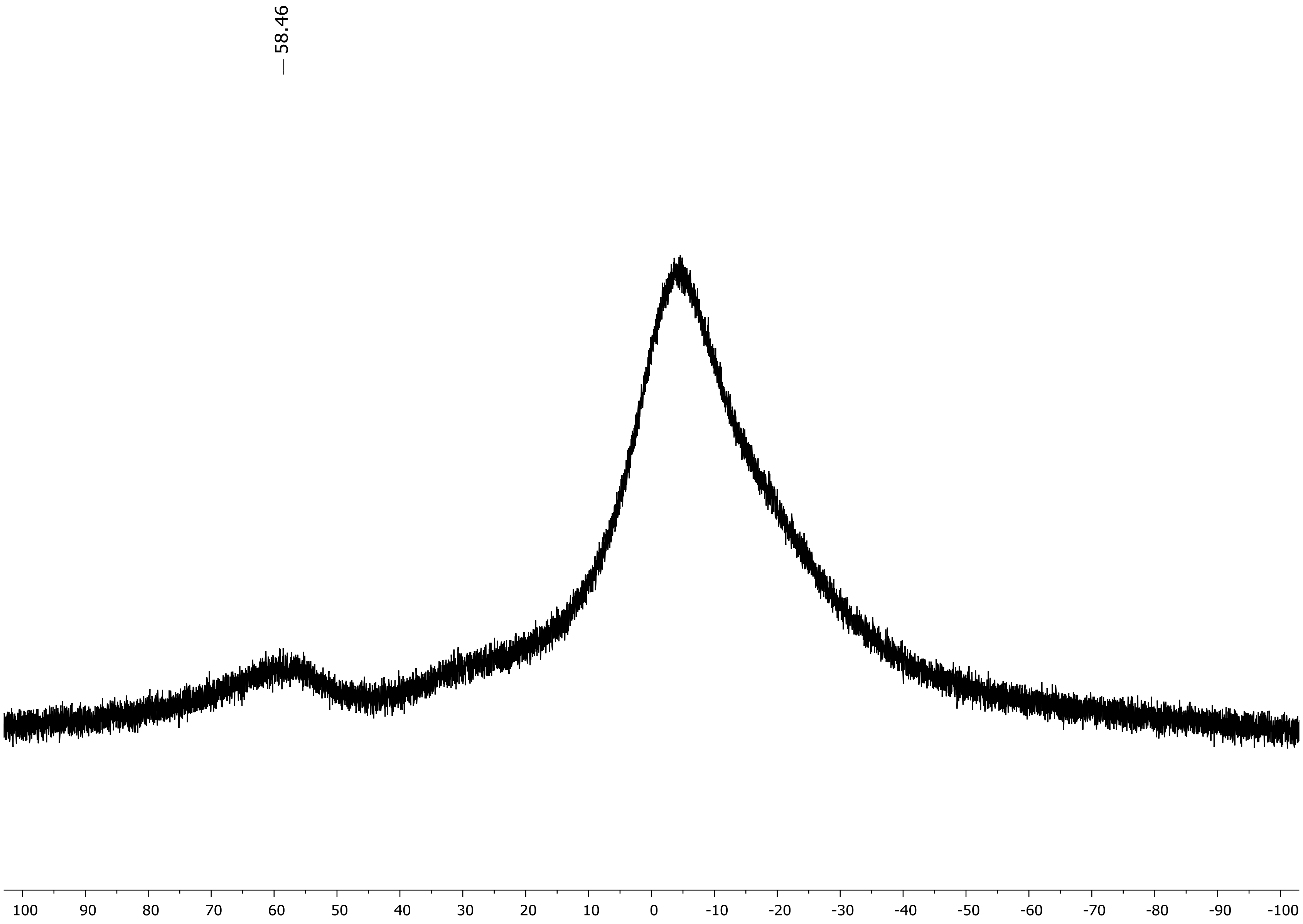

Figure S6 ^{1}H NMR (400 MHz, $CDCl_3$, Me_4Si, 295K) spectrum of **S1**.

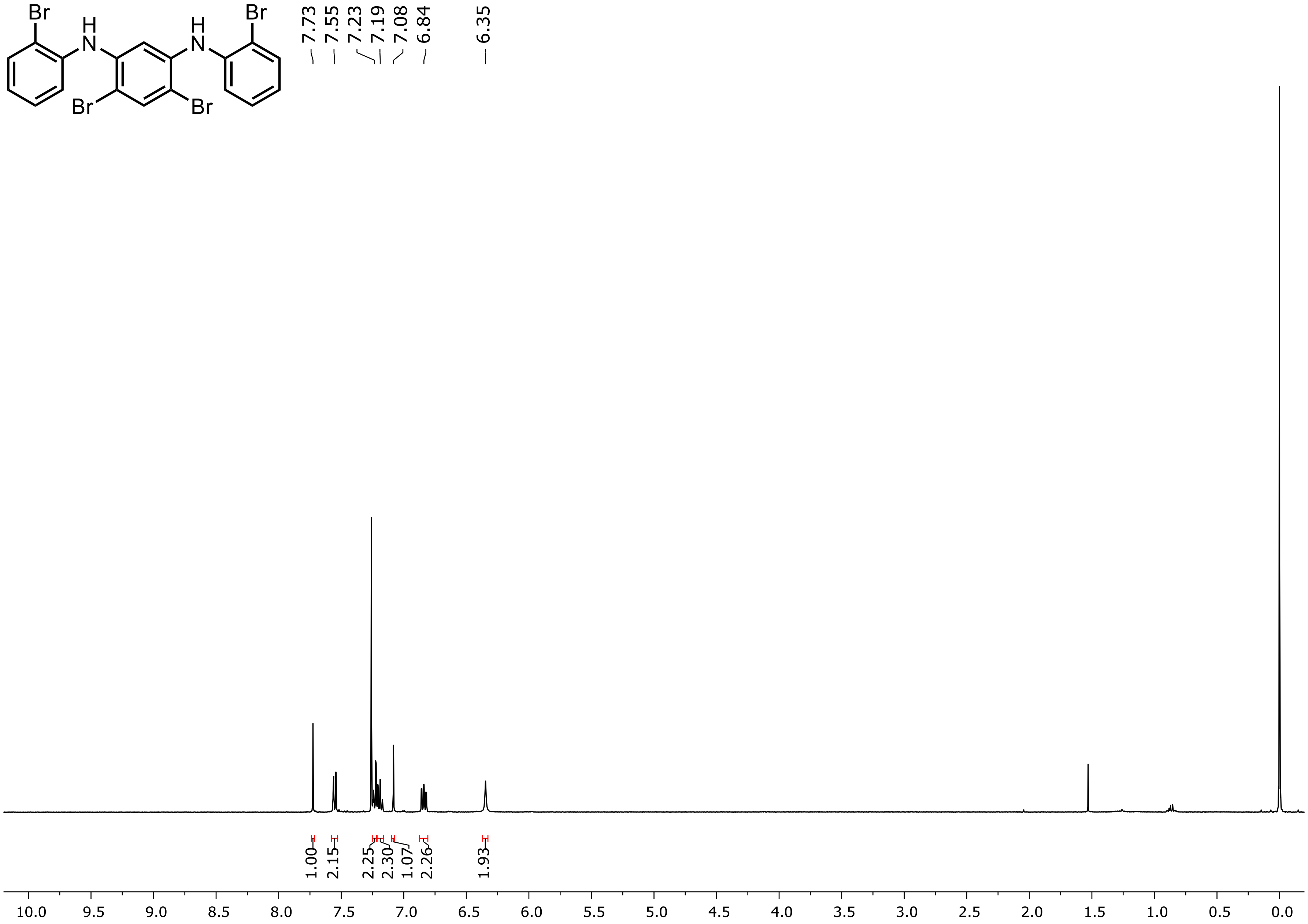

Figure S7 ^{13}C{H} NMR (101 MHz, $CDCl_3$, Me_4Si, 295K) spectrum of **S1**

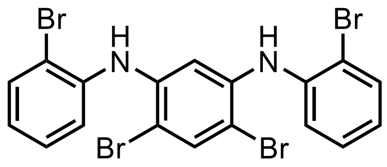

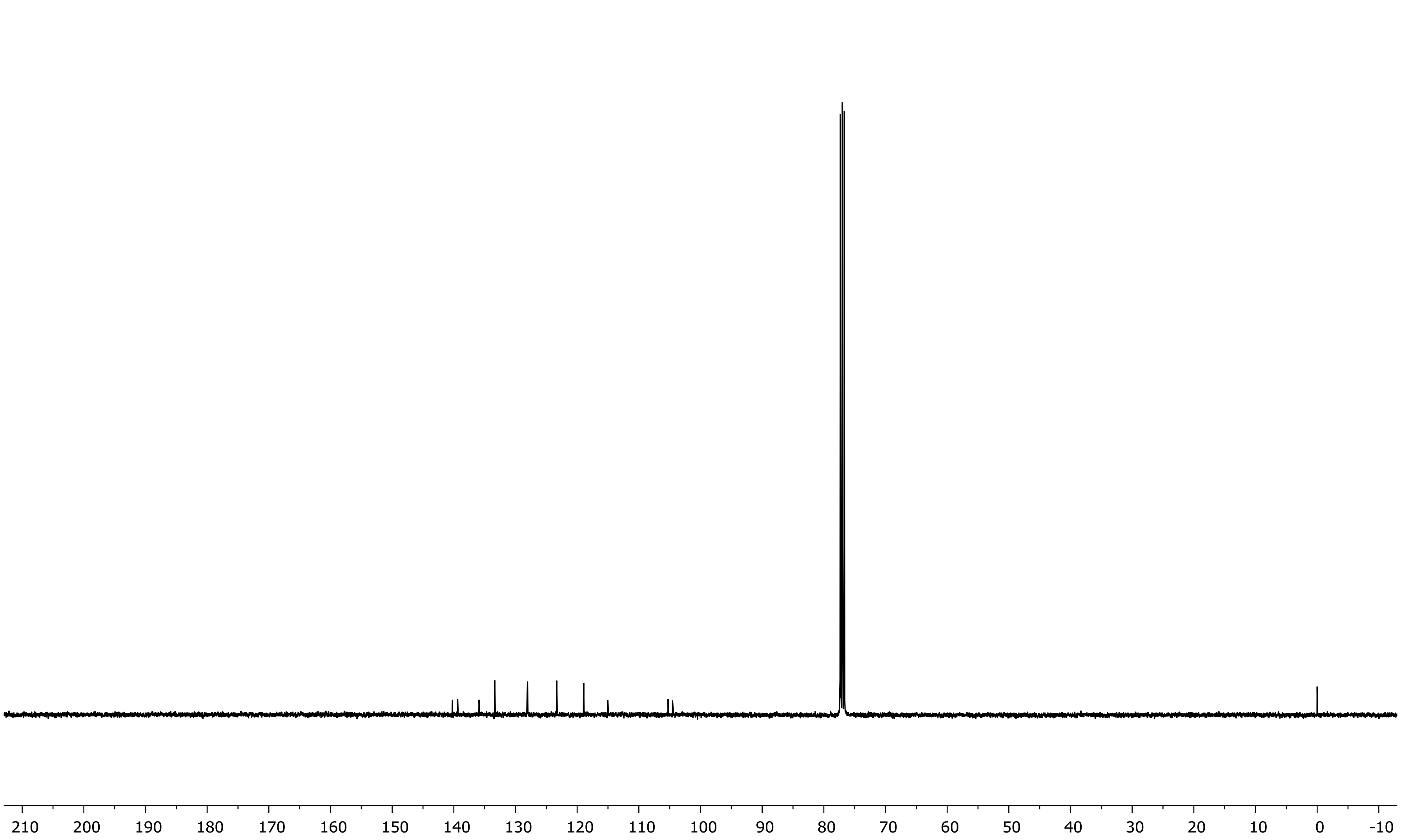

Figure S8 ^{1}**H NMR** (400 MHz, $CDCl_3$, Me_4Si, 295K) spectrum of **4**.

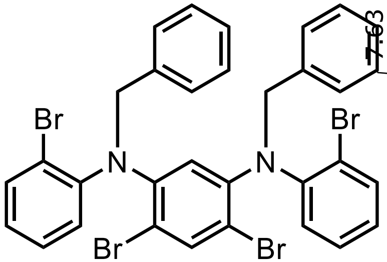

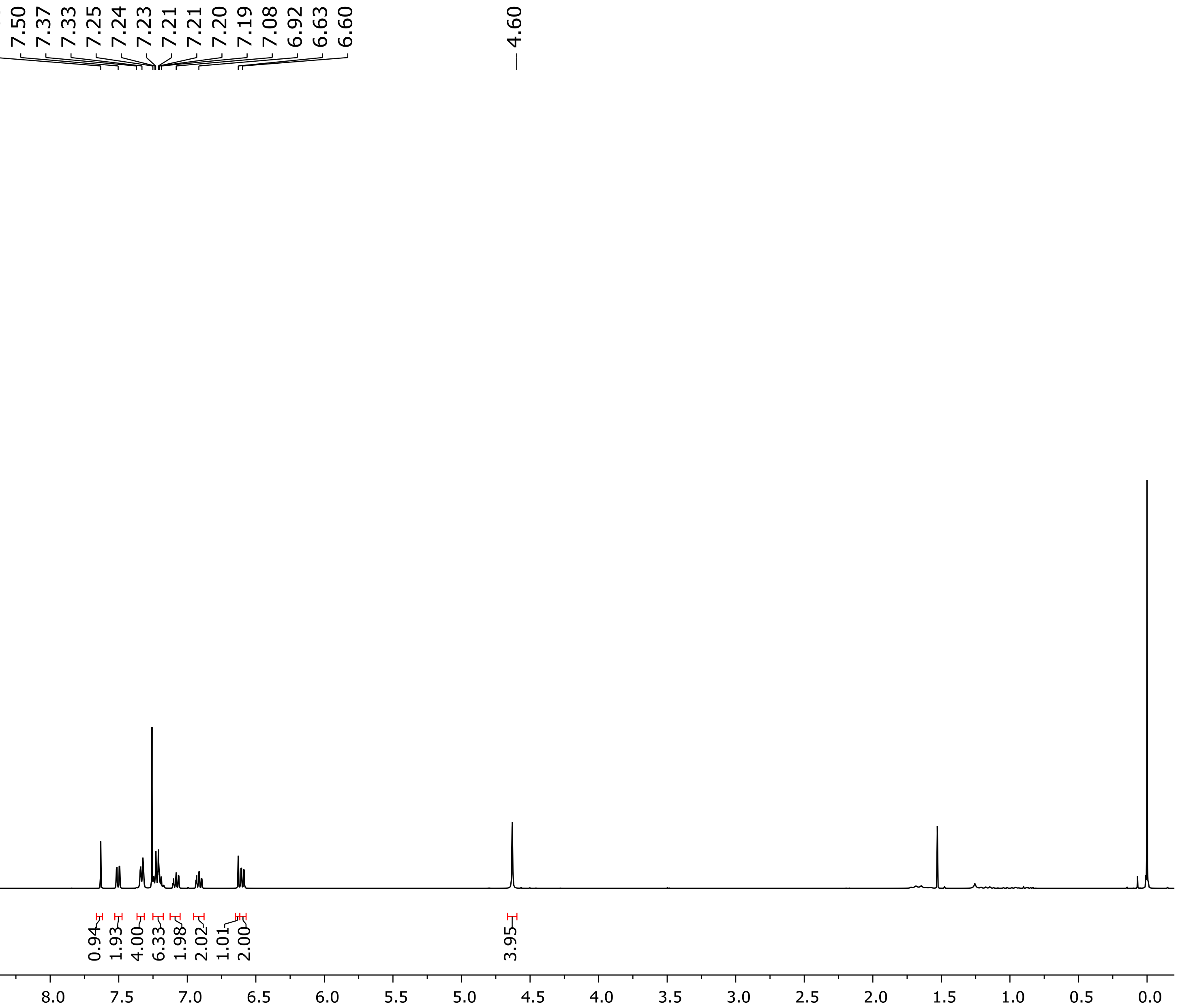

Figure S9 ^{13}C{H} NMR (101 MHz, $CDCl_3$, Me_4Si, 295K) spectrum of **4**.

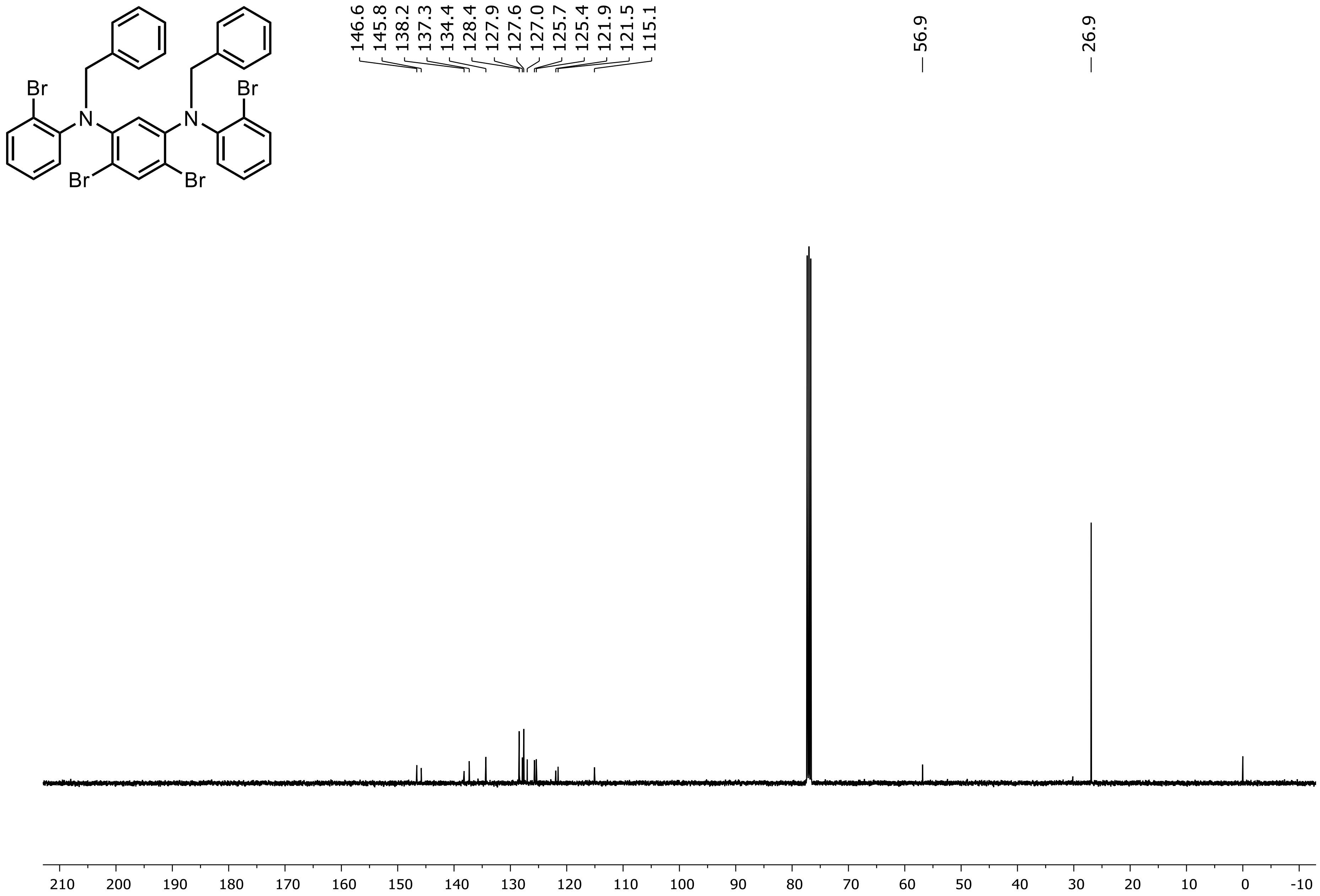

Figure S10 ^{1}H NMR (400 MHz, $CDCl_3$, Me_4Si, 295K) spectrum of **2**.

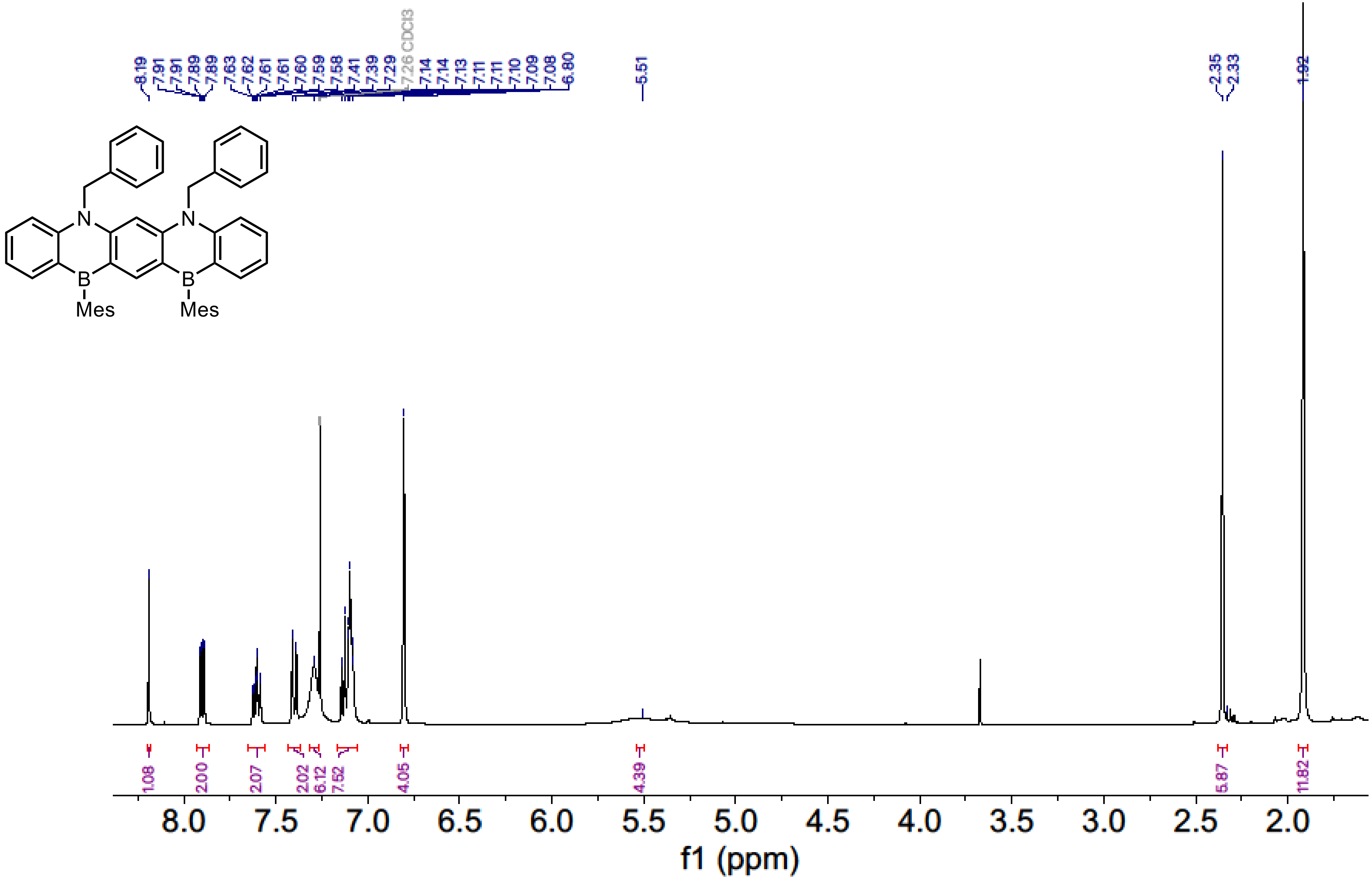

Figure S11 ^{13}C{H} NMR (101 MHz, $CDCl_3$, Me_4Si, 295K) spectrum of **2**.

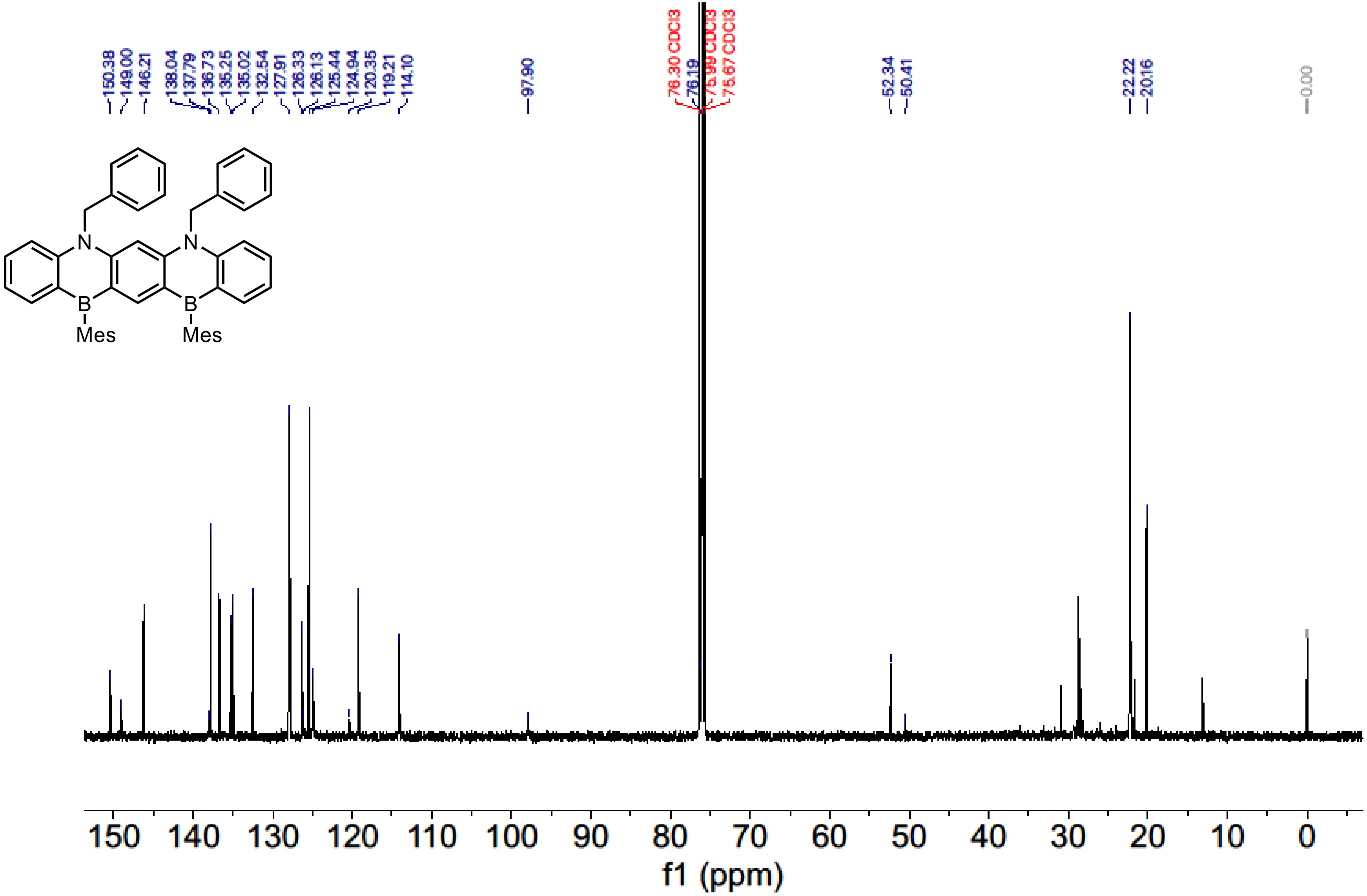

Figure S12 ^{11}B NMR (376 MHz, $CDCl_3$, BF_3Et_2O, 295K) spectrum of **2**.

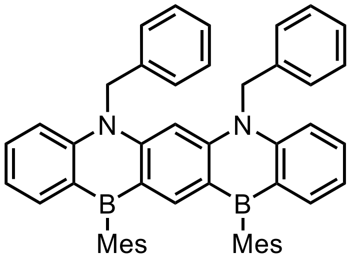

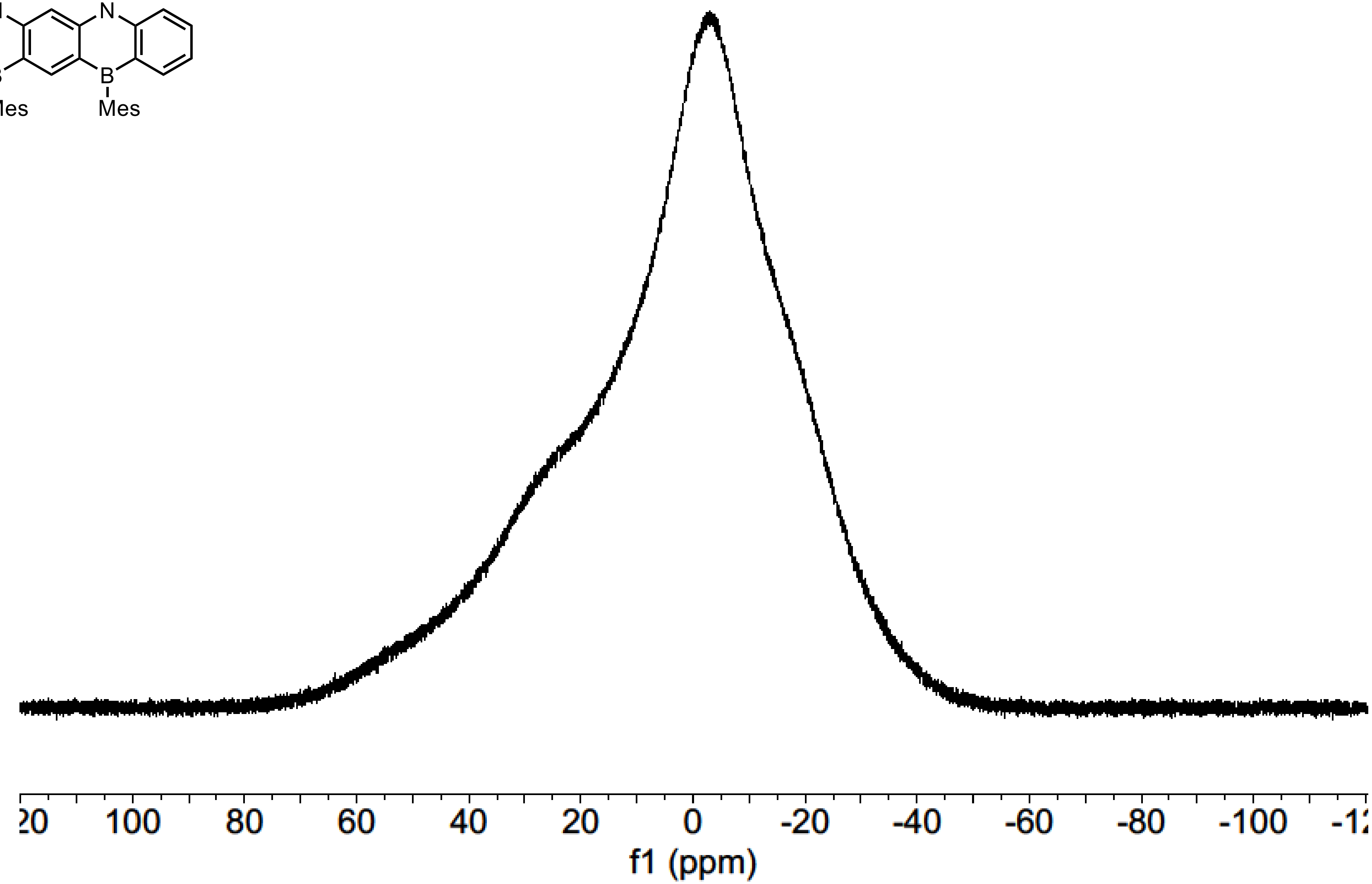

2.2 NMR of reduction products

Figure S13 ¹H NMR (400 MHz, $CDCl_3$,Me_4Si, 295K) spectrum of **5a**

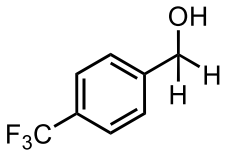

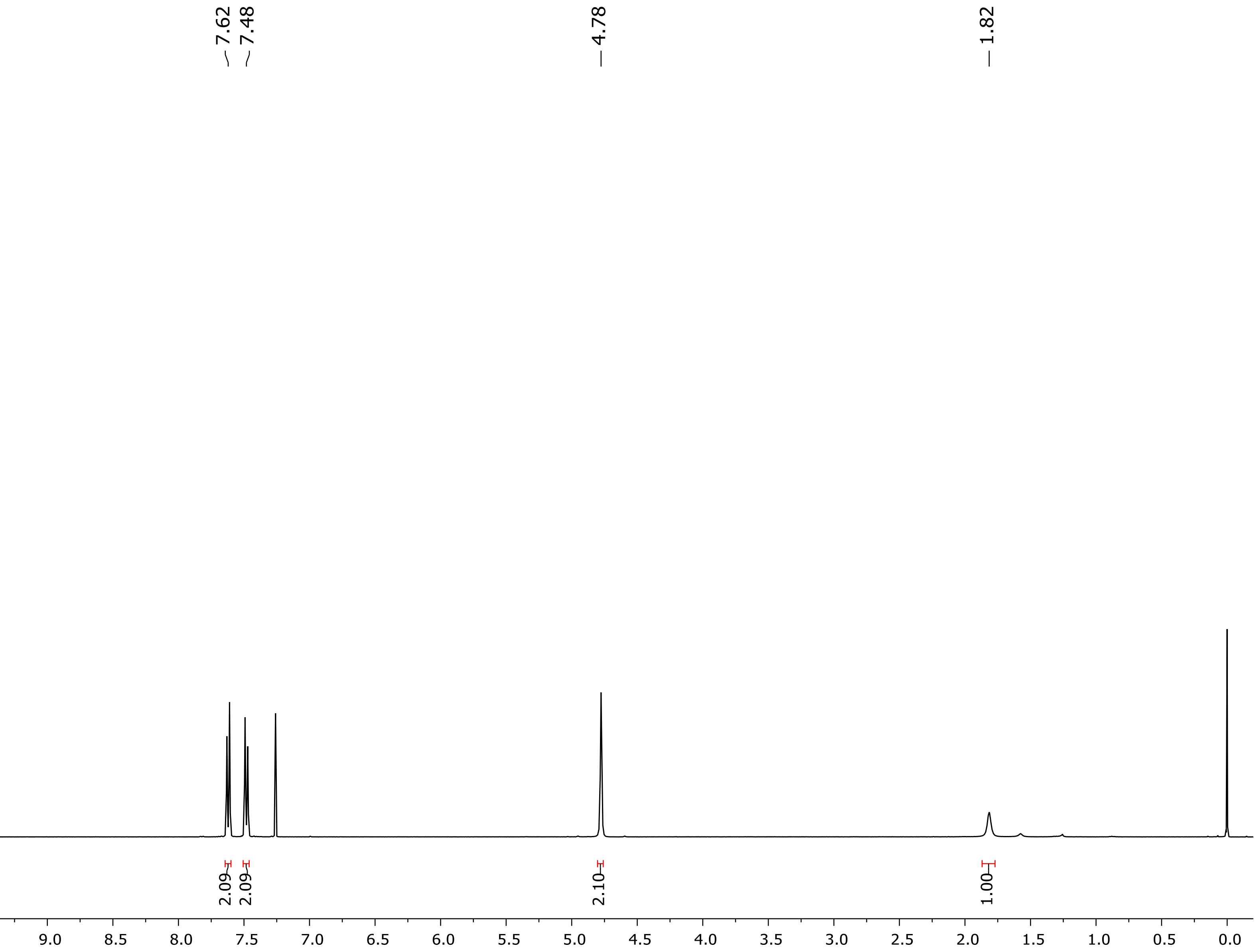

Figure S14 $^{13}C\{^1H\}$ NMR (101 MHz, $CDCl_3$, Me_4Si, 295K) spectrum of **5a**.

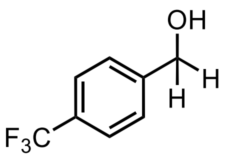

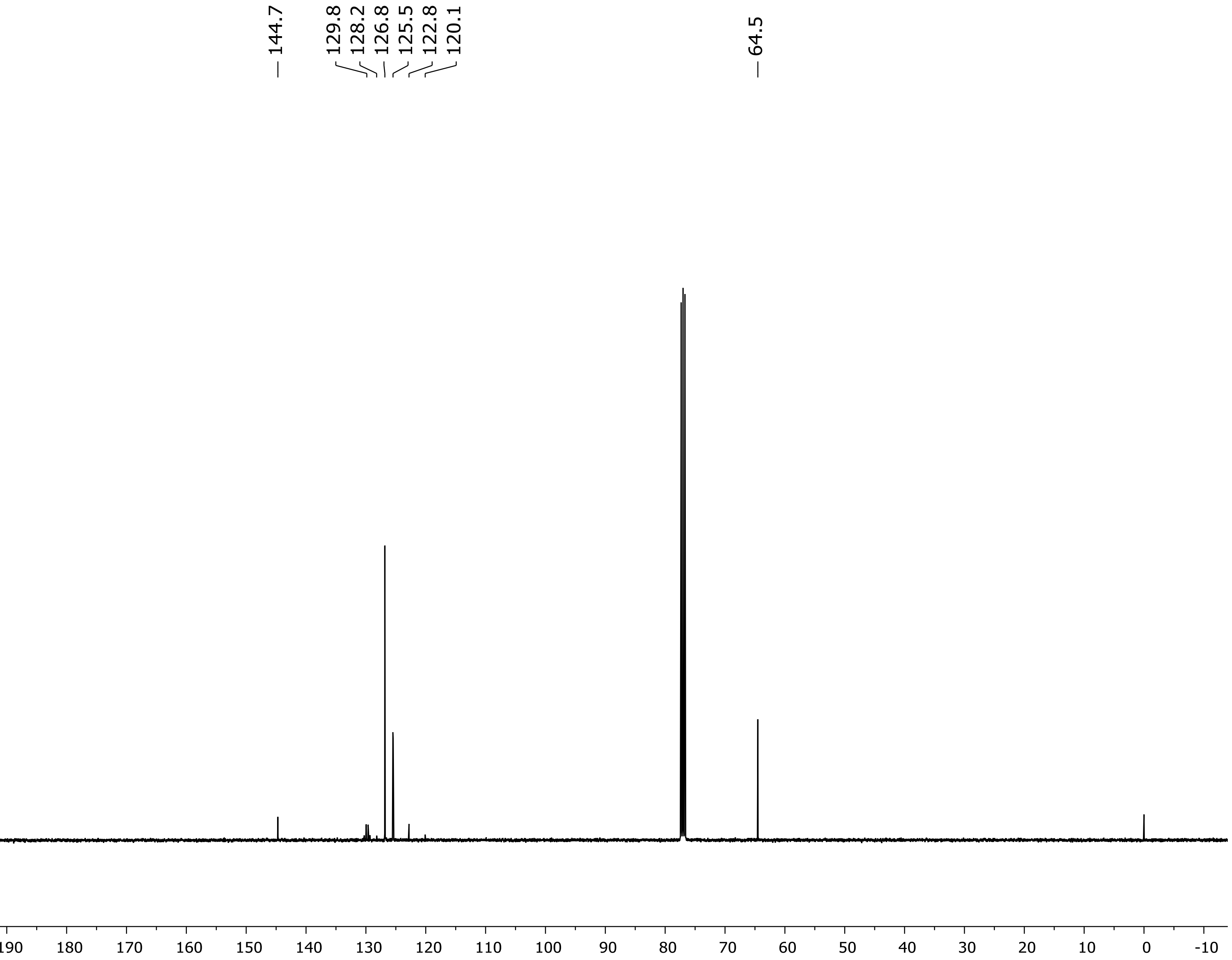

Figure S15 ^{1}H NMR (400 MHz, $CDCl_3$,Me_4Si, 295K) spectrum of **5b**.

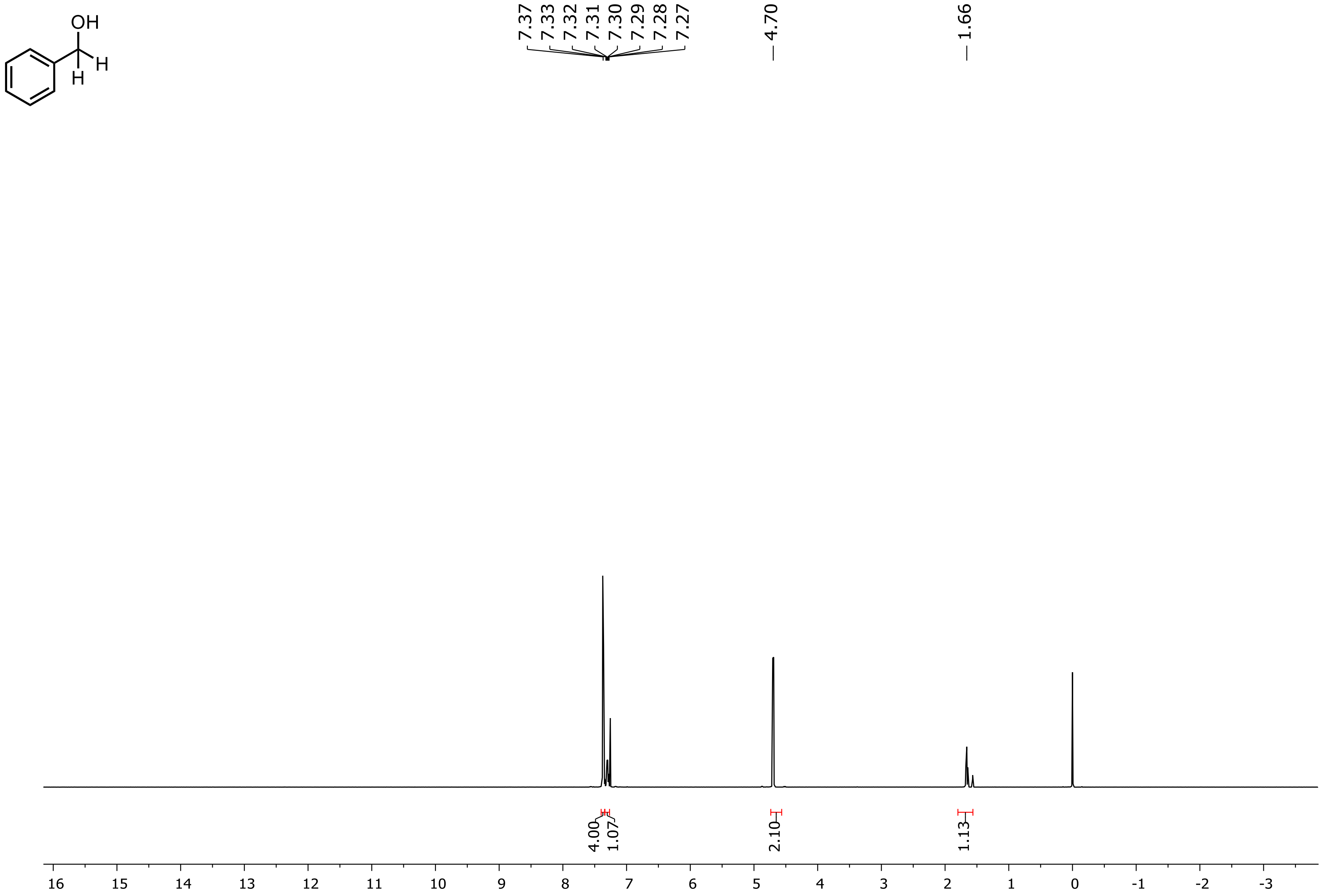

Figure S16 ^{13}**C{**^{1}**H} NMR** (101 MHz, $CDCl_3$, Me_4Si, 295K) spectrum of **5b**.

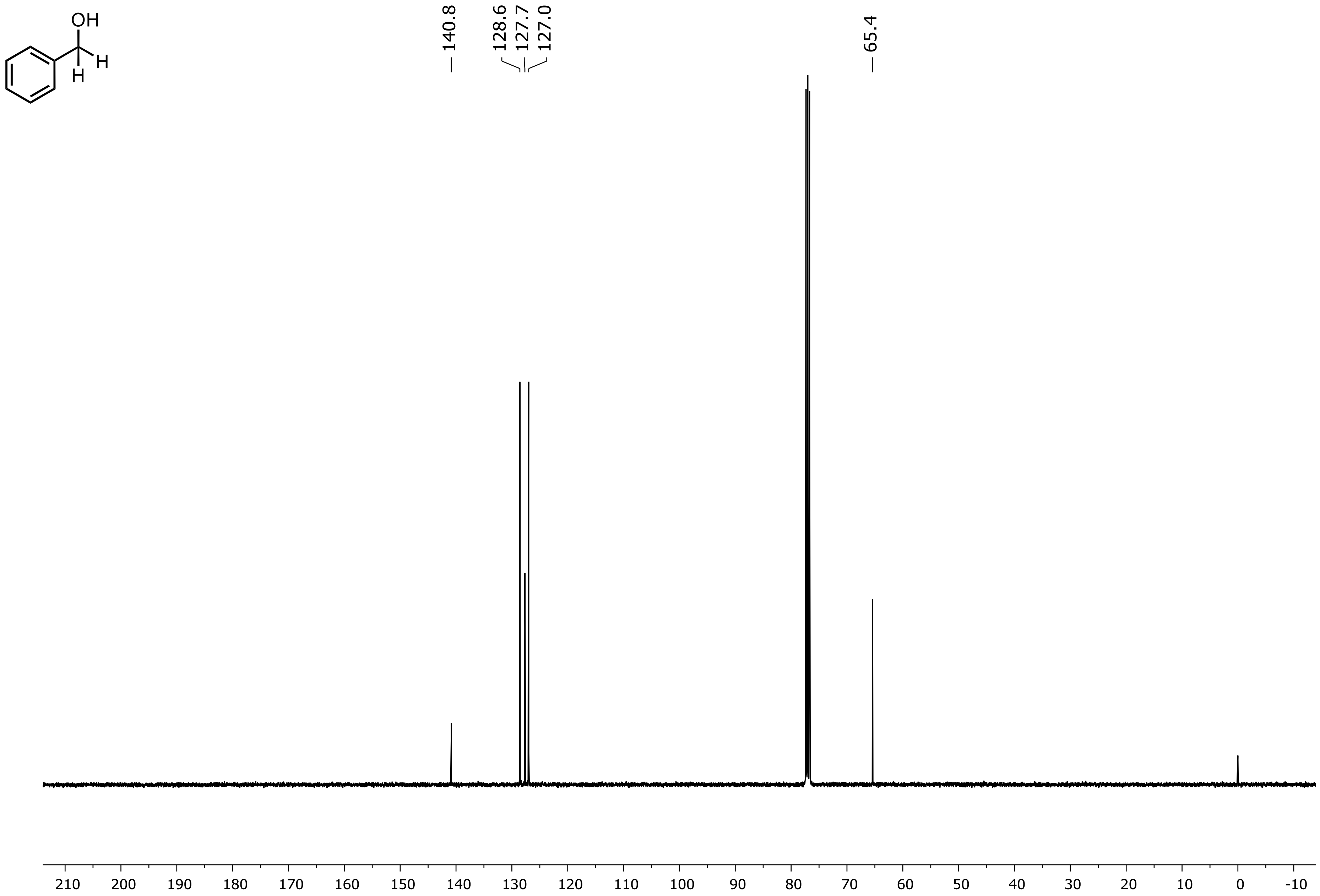

Figure S17 ^{1}H NMR (400 MHz, $CDCl_3$,Me_4Si, 295K) spectrum of **5c**.

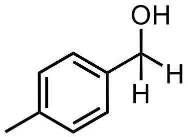

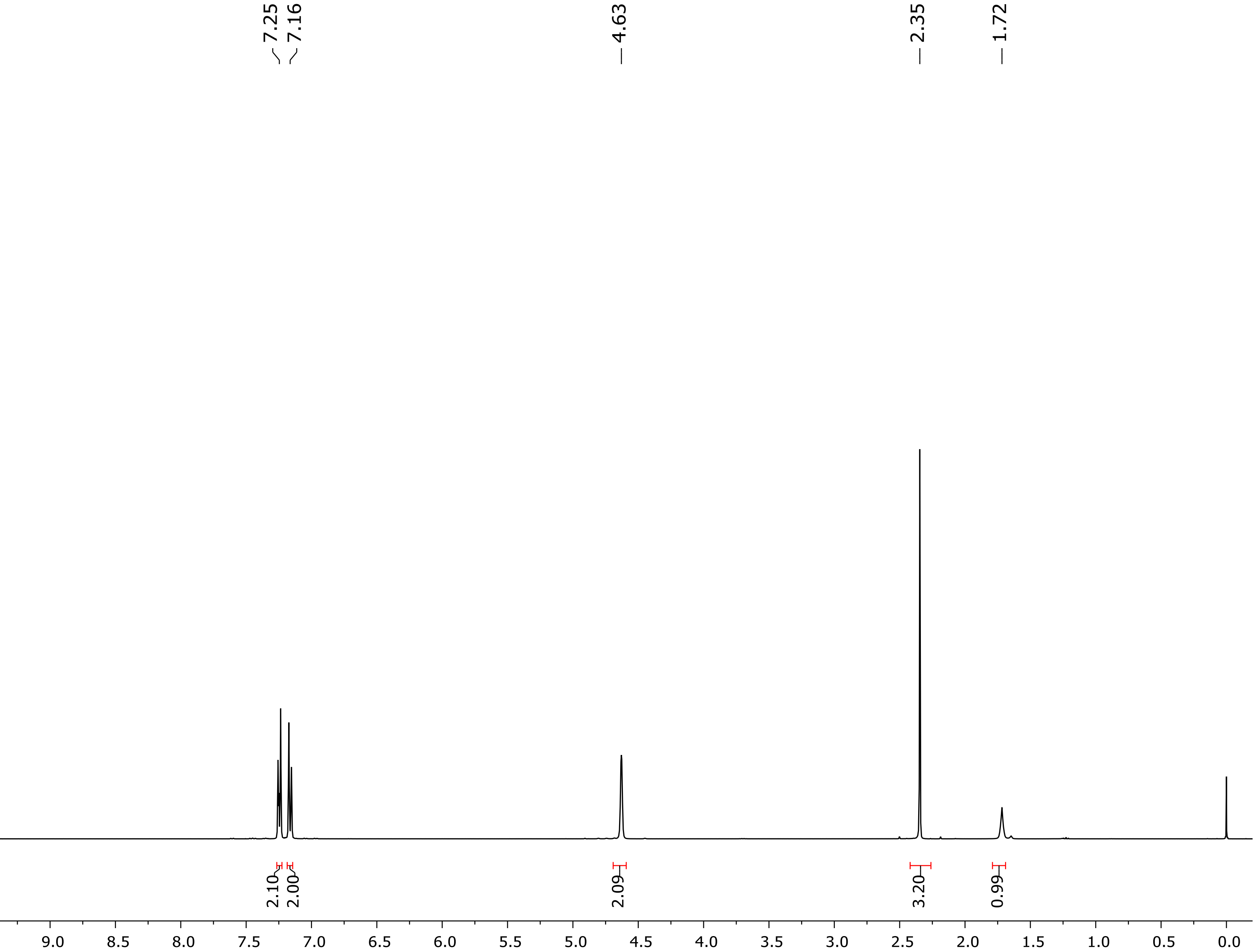

Figure S18 $^{13}C\{^{1}H\}$ NMR (101 MHz, $CDCl_3$, Me_4Si, 295K) spectrum of **5c**.

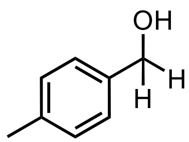

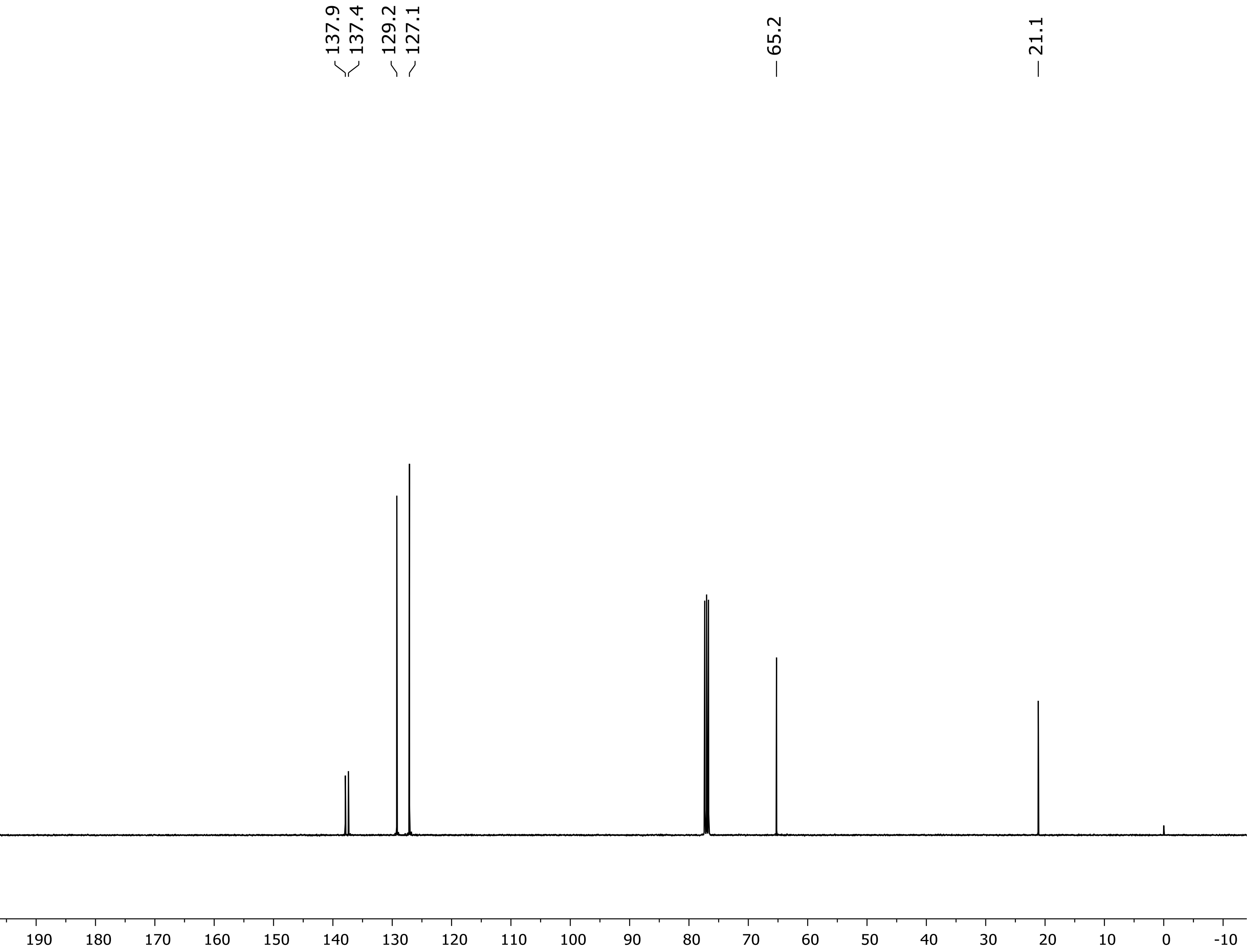

Figure S19 ^{1}H NMR (400 MHz, $CDCl_3$,Me_4Si, 295K) spectrum of **5d**.

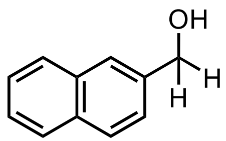

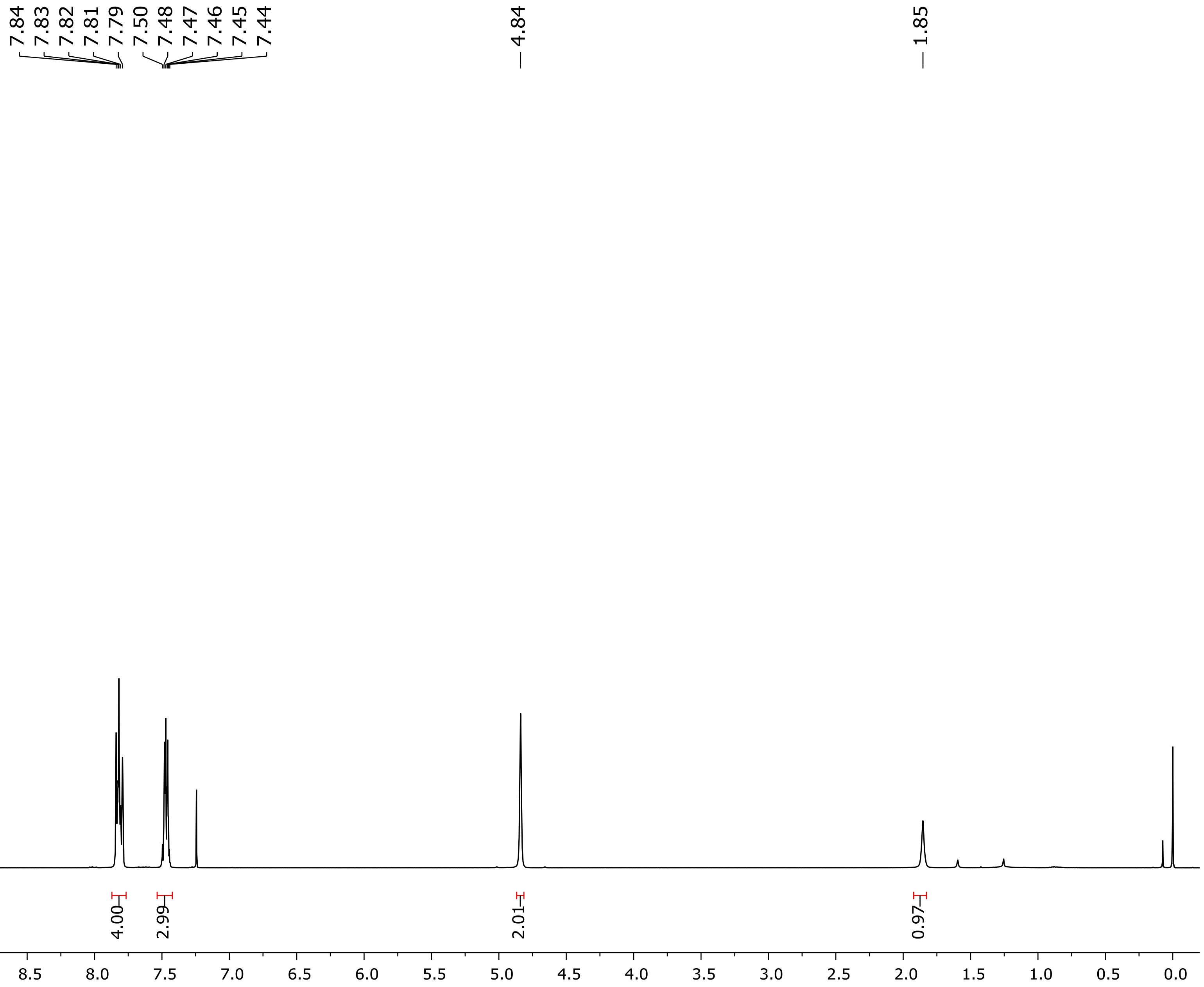

Figure S20 $^{13}C\{^1H\}$ **NMR** (101 MHz, $CDCl_3$, Me_4Si, 295K) spectrum of **5d**.

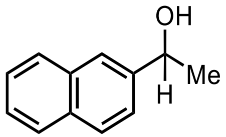

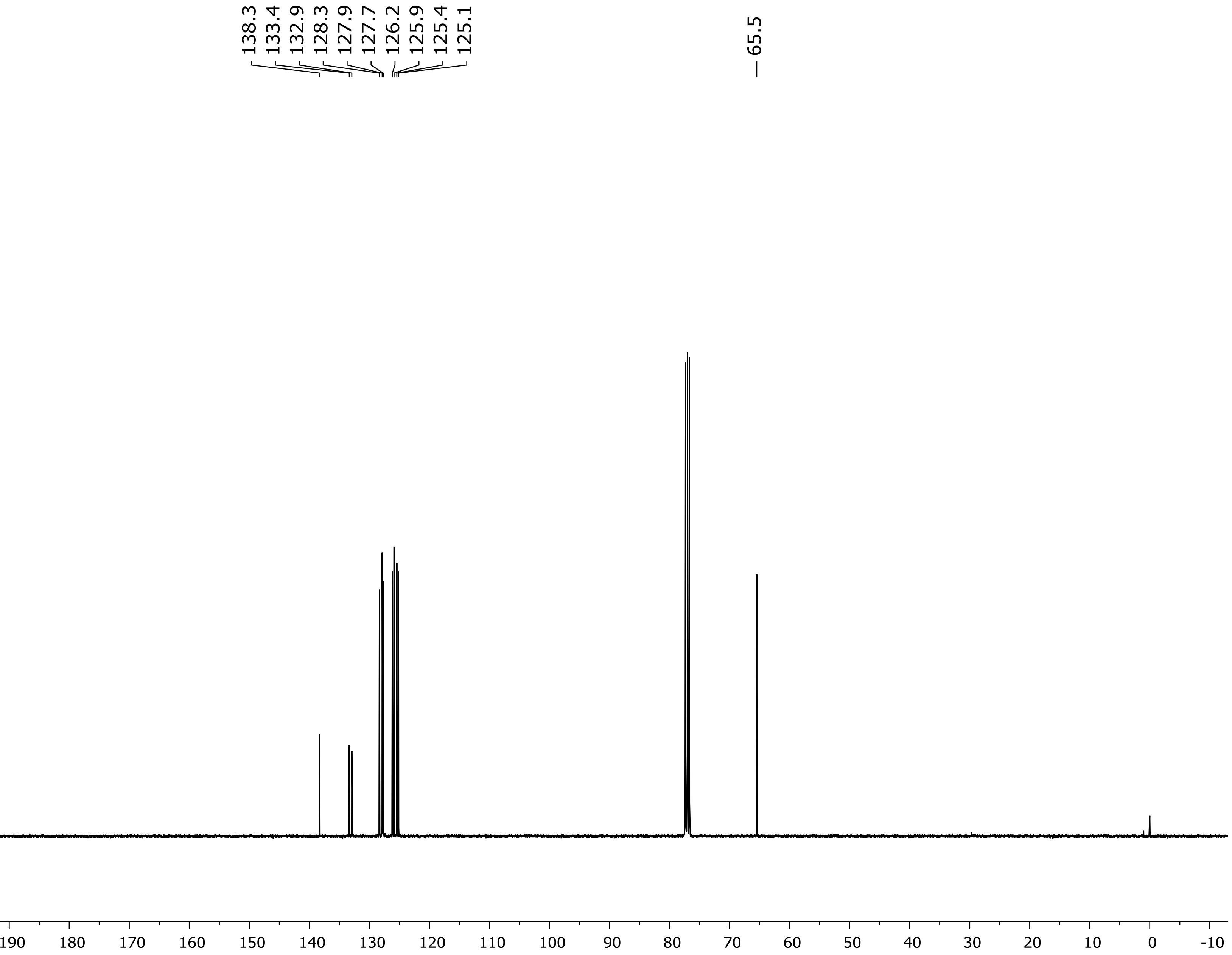

Figure S21 **^{1}H NMR** (400 MHz, $CDCl_3$,Me_4Si, 295K) spectrum of **6a**.

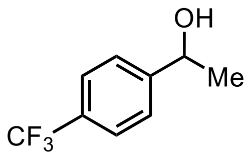

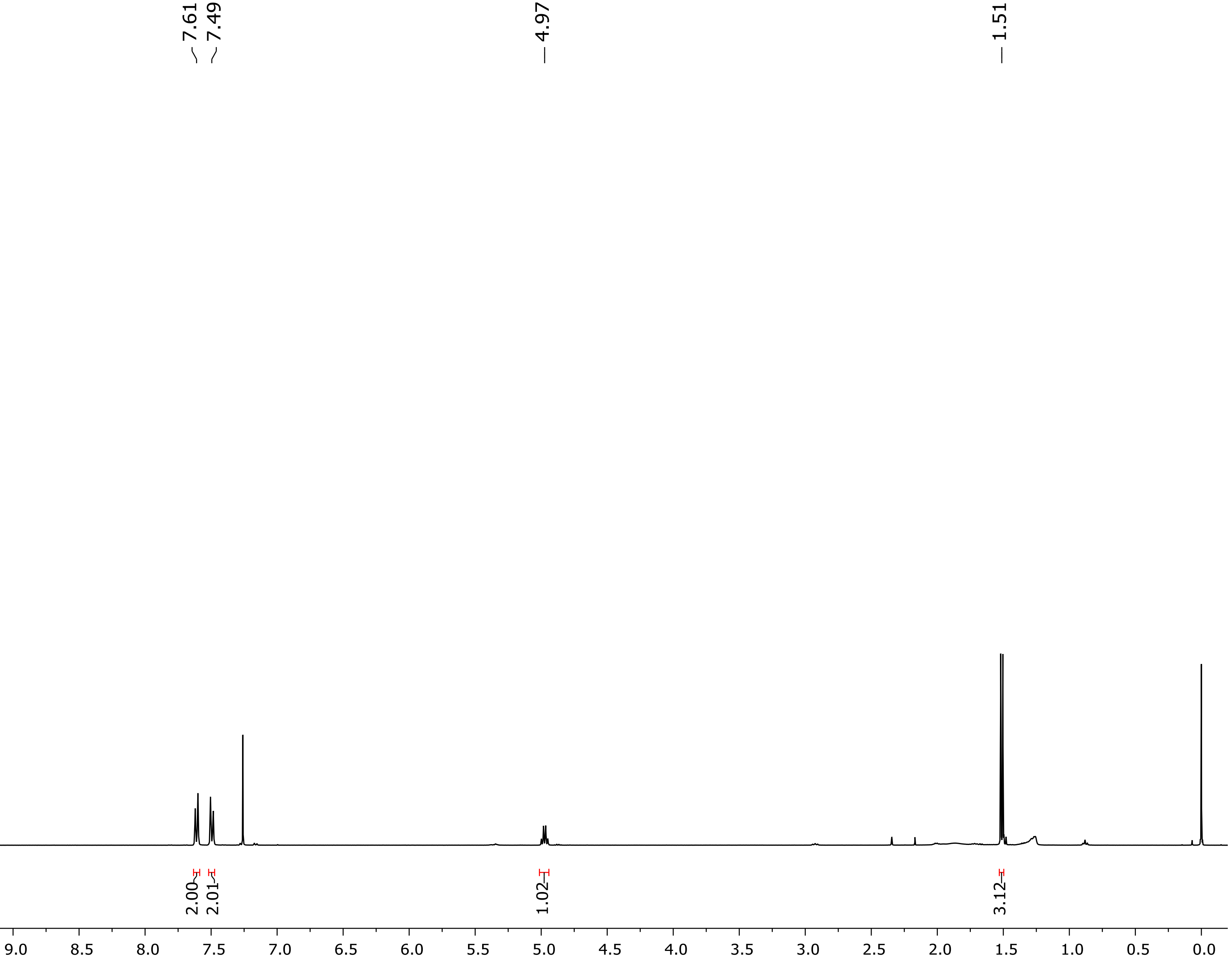

Figure S22 ^{13}**C{^{1}H} NMR** (101 MHz, $CDCl_3$, Me_4Si, 295K) spectrum of **6a**.

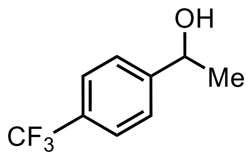

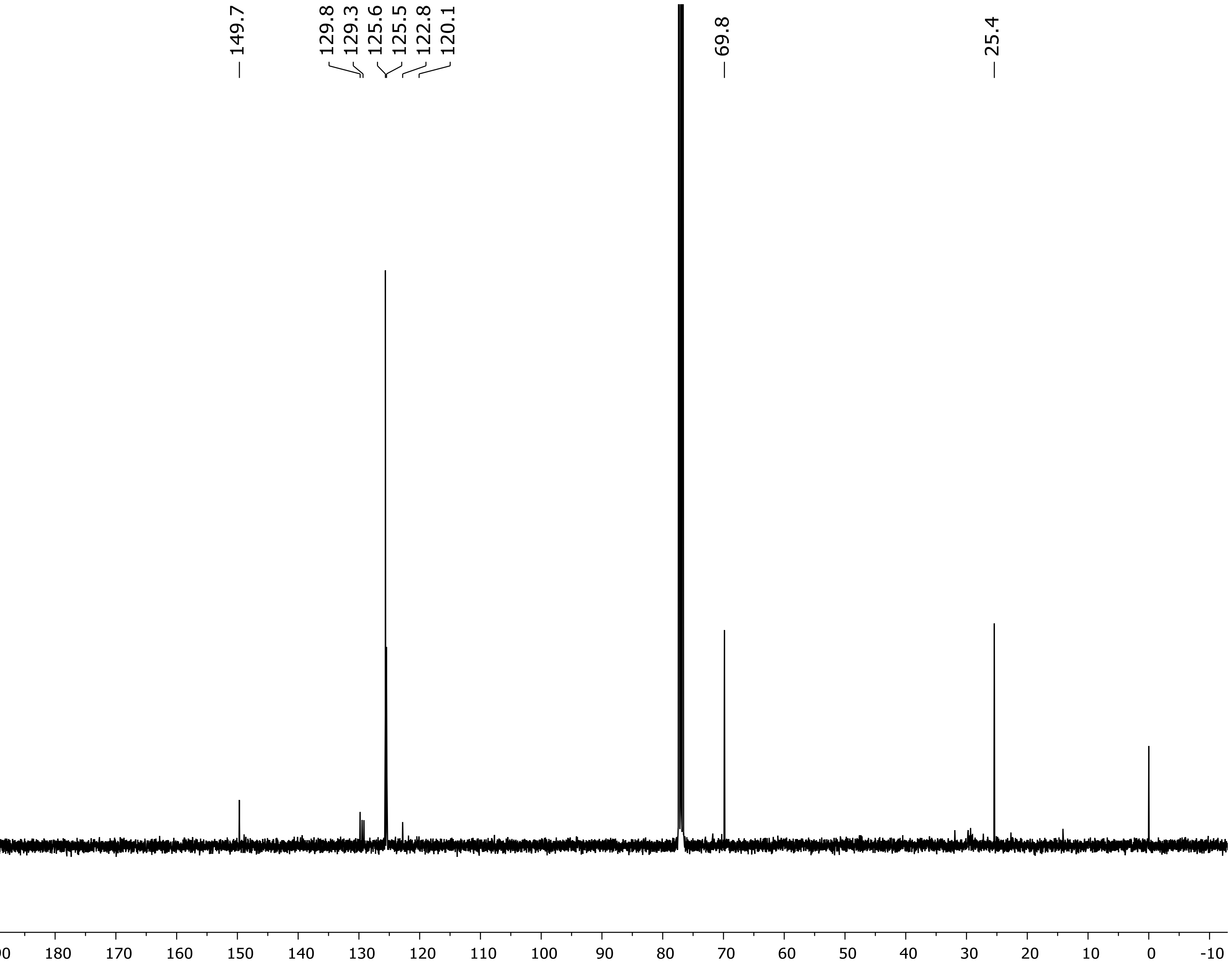

Figure S23 **^{1}H NMR** (400 MHz, $CDCl_3$,Me_4Si, 295K) spectrum of **6b**.

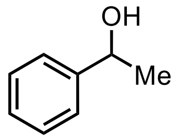

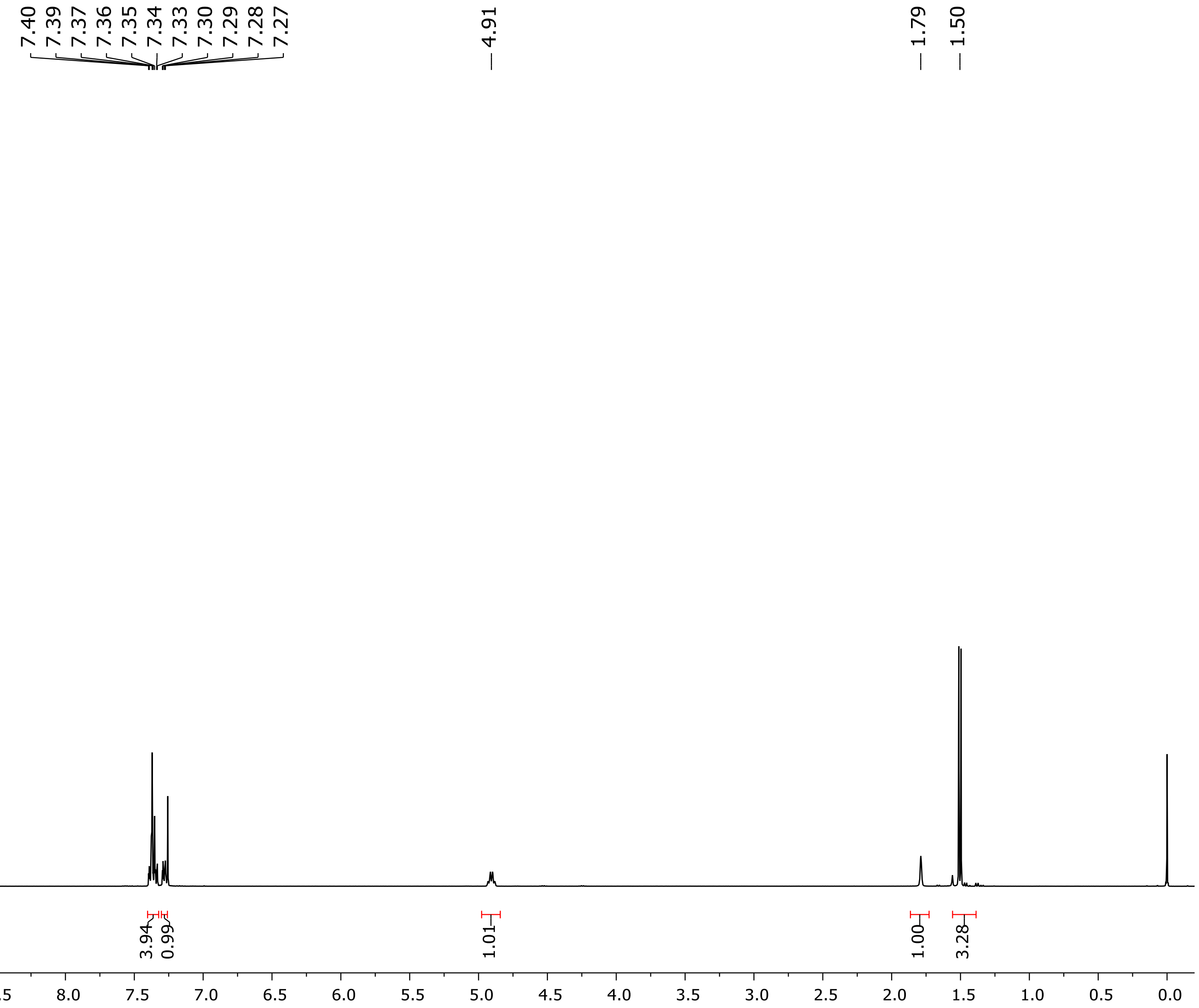

Figure S24 $^{13}C\{^{1}H\}$ NMR (101 MHz, $CDCl_3$, Me_4Si, 295K) spectrum of **6b**.

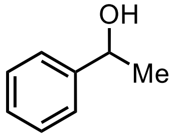

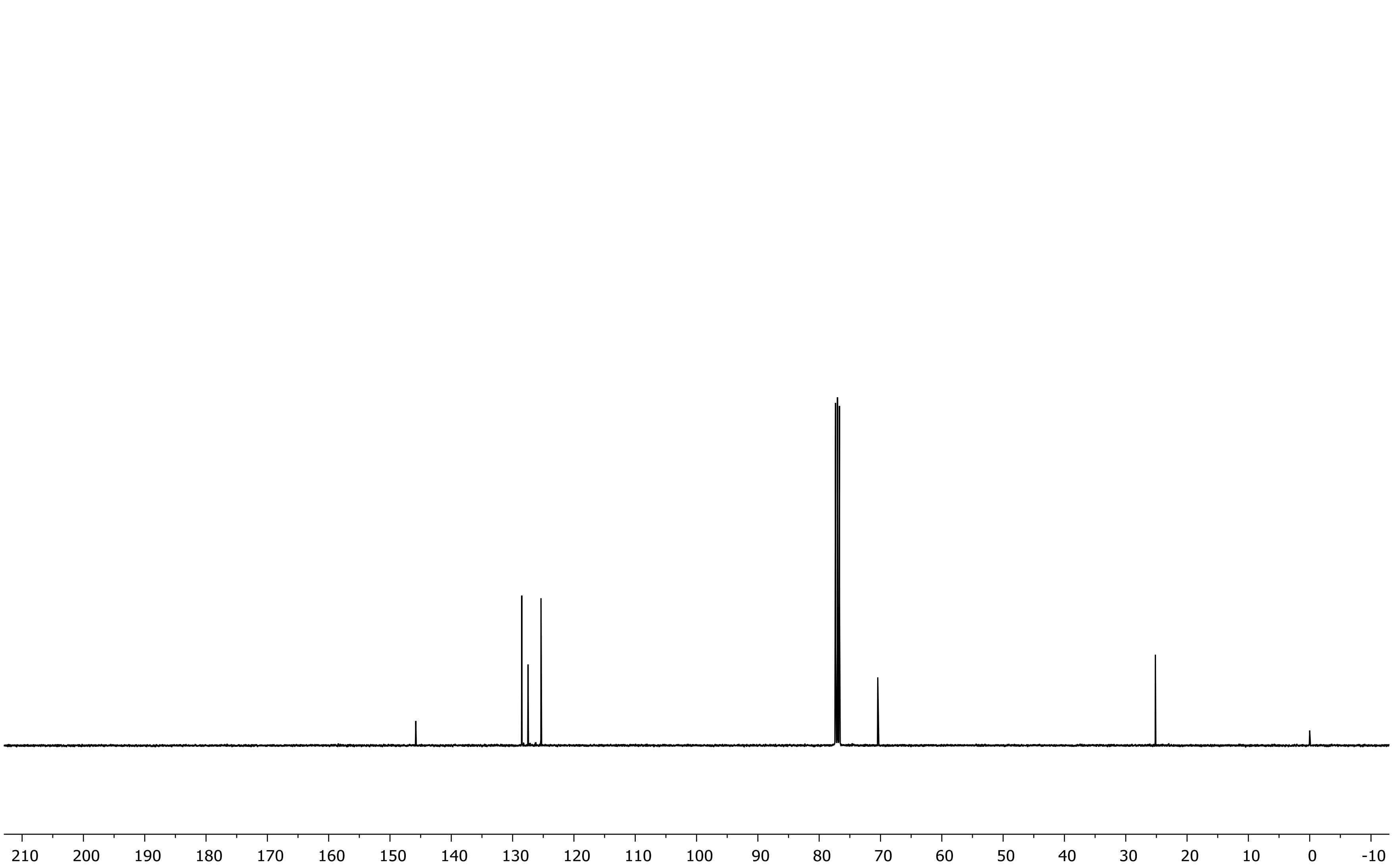

Figure S25 ^{1}**H NMR** (400 MHz, $CDCl_3$,Me_4Si, 295K) spectrum of **6c**.

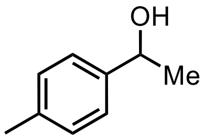

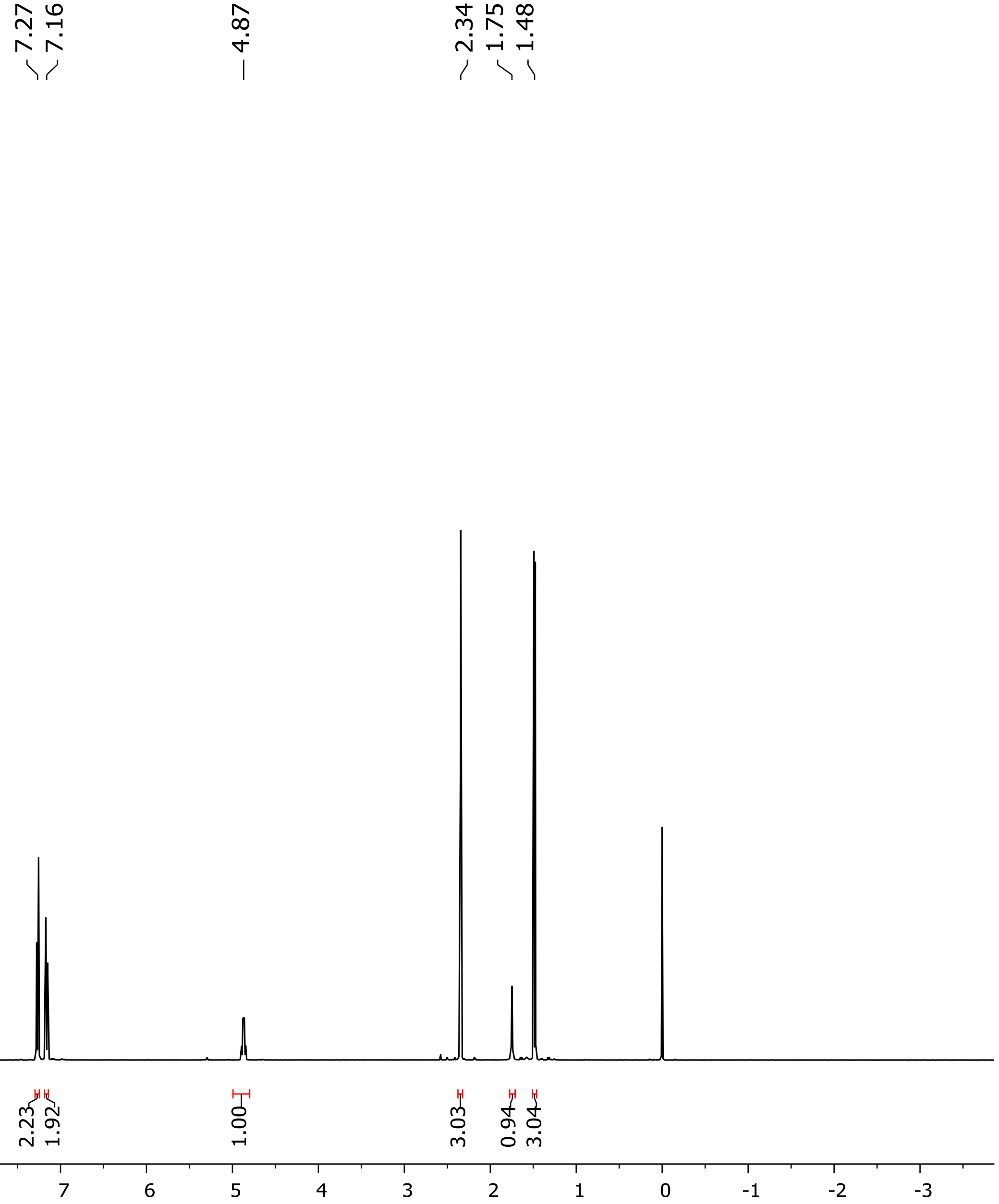

Figure S26 $^{13}C\{^{1}H\}$ NMR (101 MHz, $CDCl_3$, Me_4Si, 295K) spectrum of **6c**.

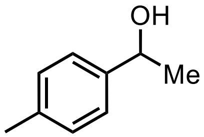

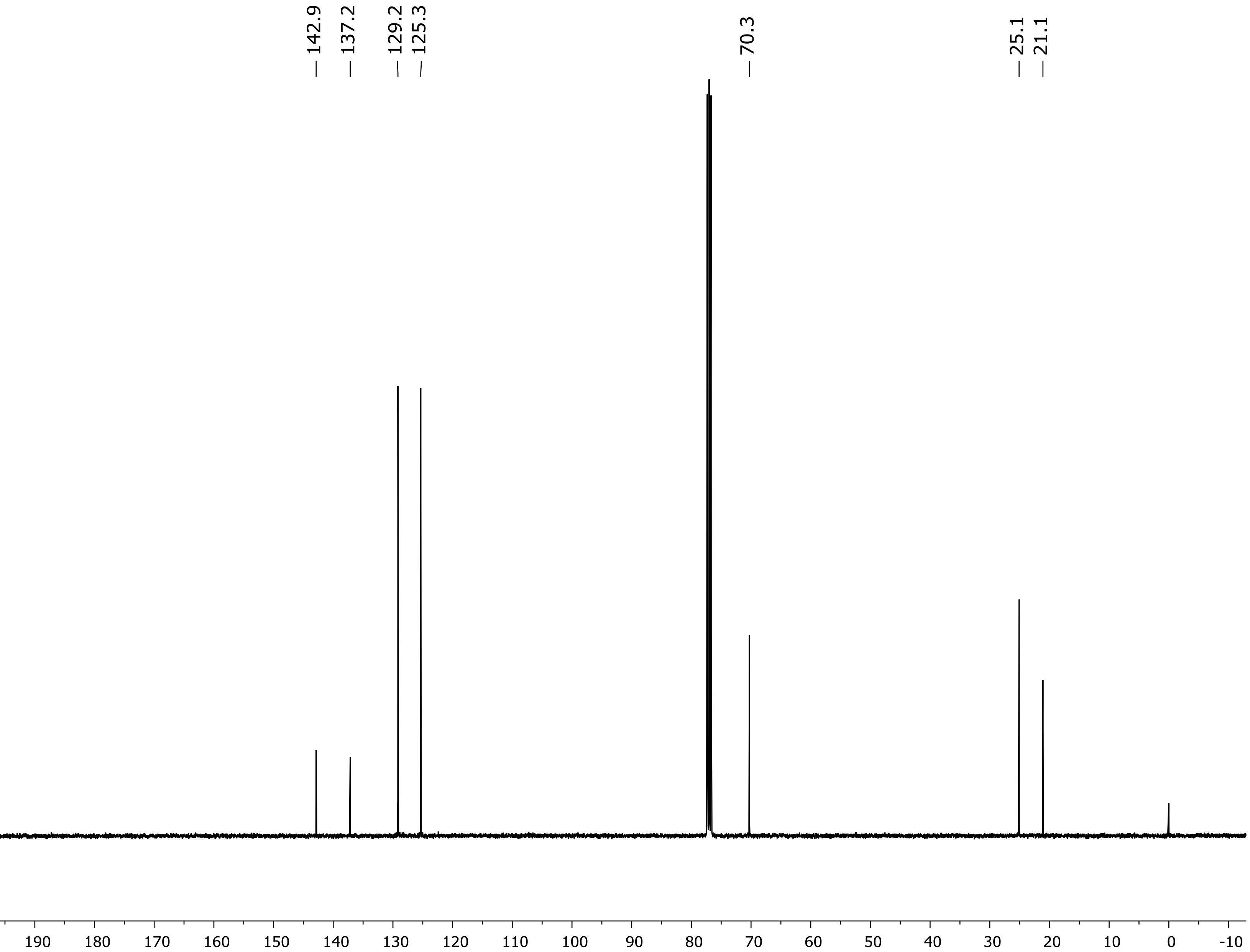

Figure S27 ^{1}H NMR (400 MHz, $CDCl_3$,Me_4Si, 295K) spectrum of **6d**.

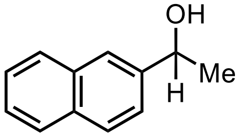

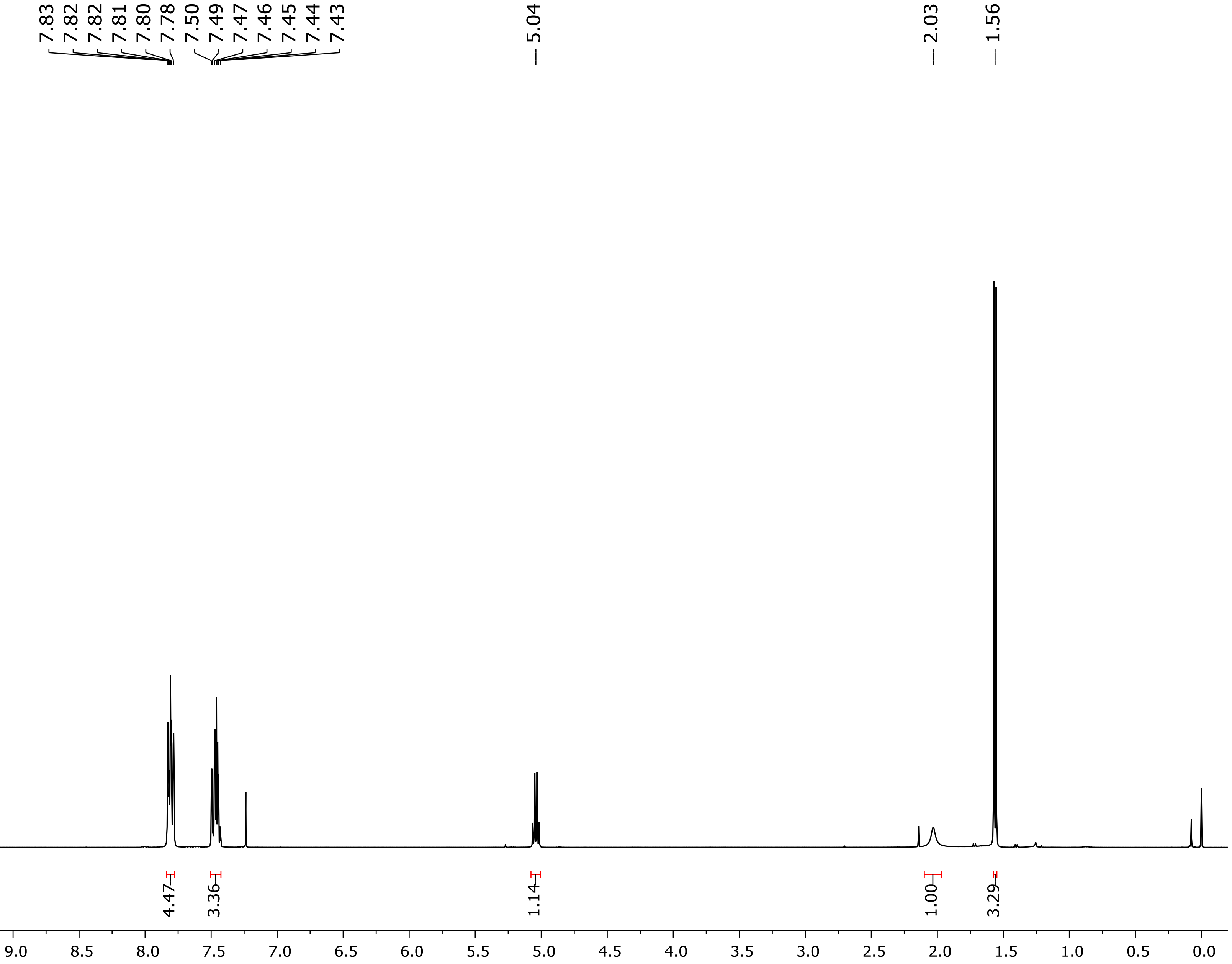

Figure S28 $^{13}C\{^{1}H\}$ NMR (101 MHz, $CDCl_3$, Me_4Si, 295K) spectrum of **6d**.

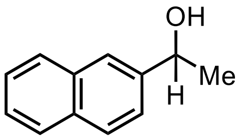

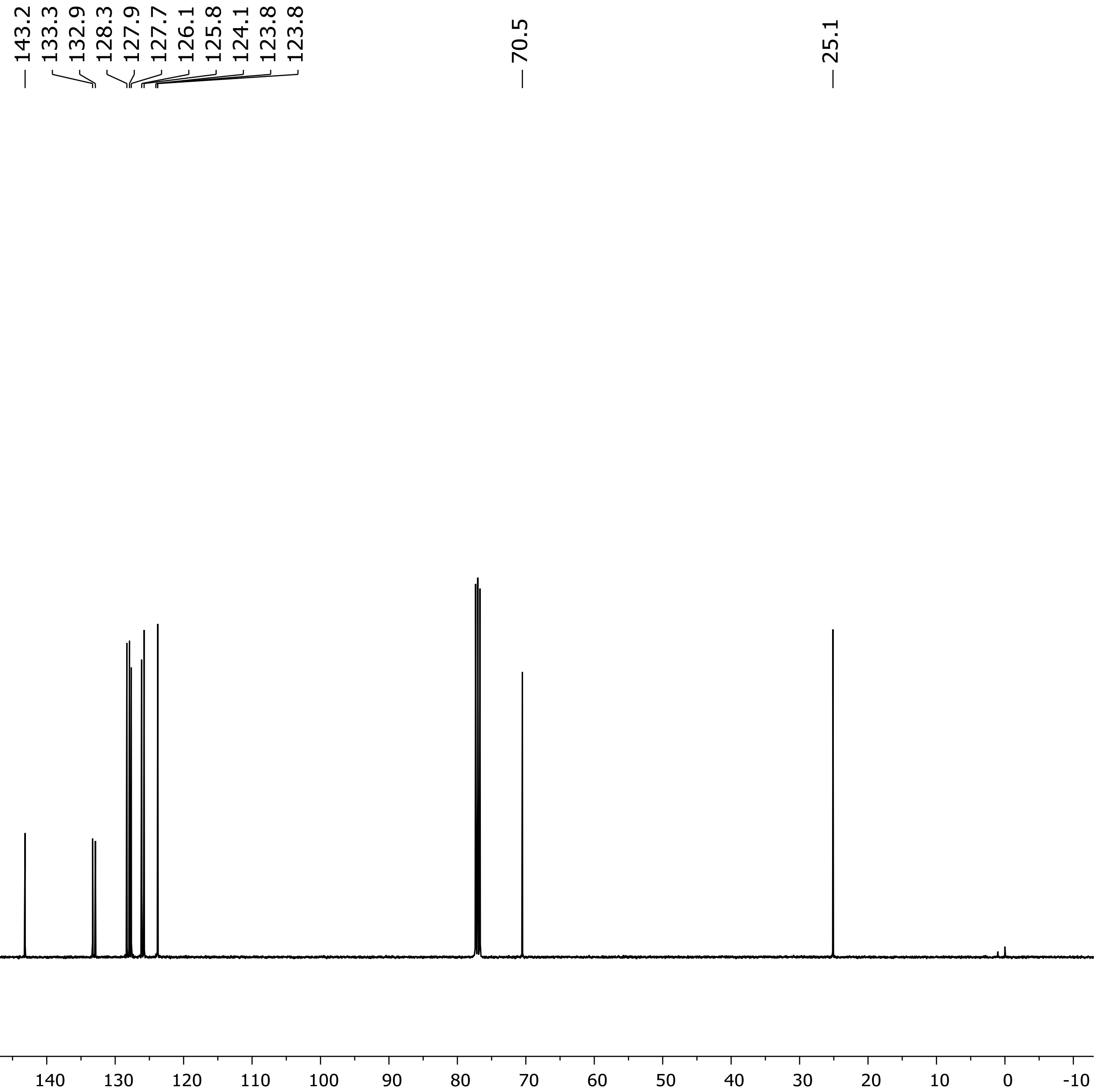

Figure S29 **^{1}H NMR** (400 MHz, $CDCl_3$,Me_4Si, 295K) spectrum of **7a**.

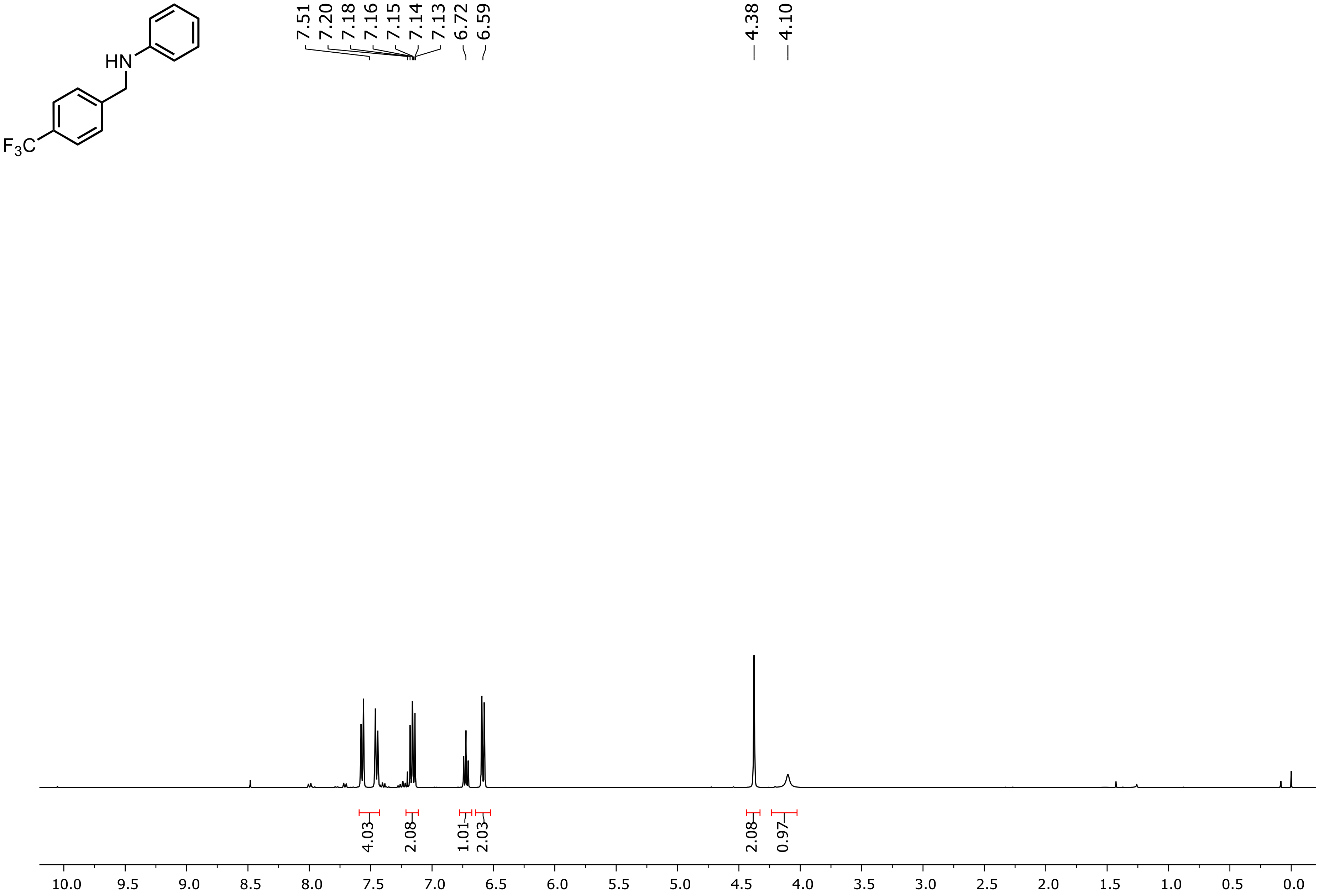

Figure S30 ^{13}**C{^{1}H} NMR** (101 MHz, $CDCl_3$, Me_4Si, 295K) spectrum of **7a**.

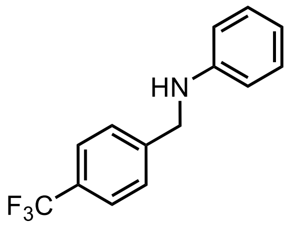

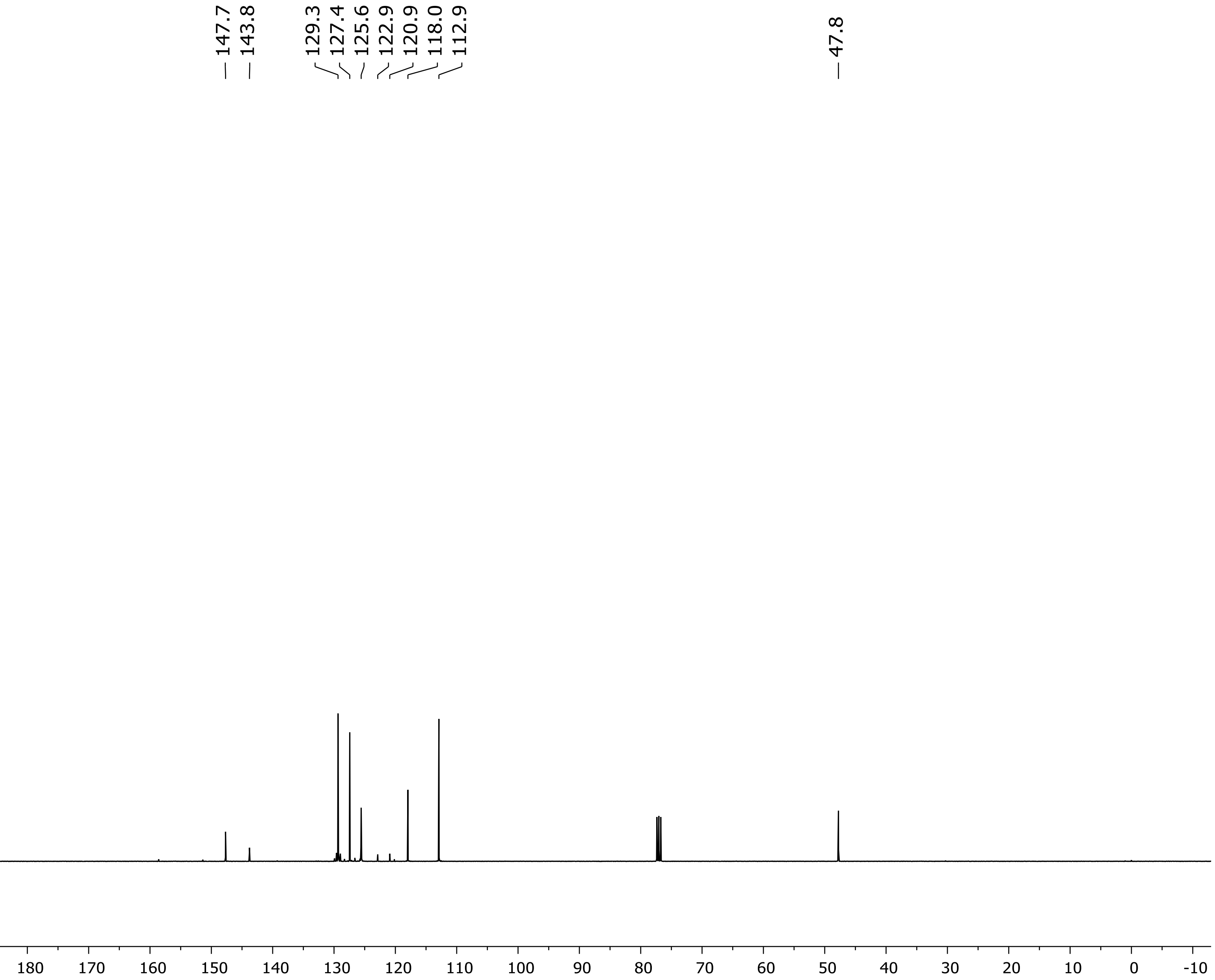

Figure S31 ^{1}H NMR (400 MHz, $CDCl_3$,Me_4Si, 295K) spectrum of **7b**.

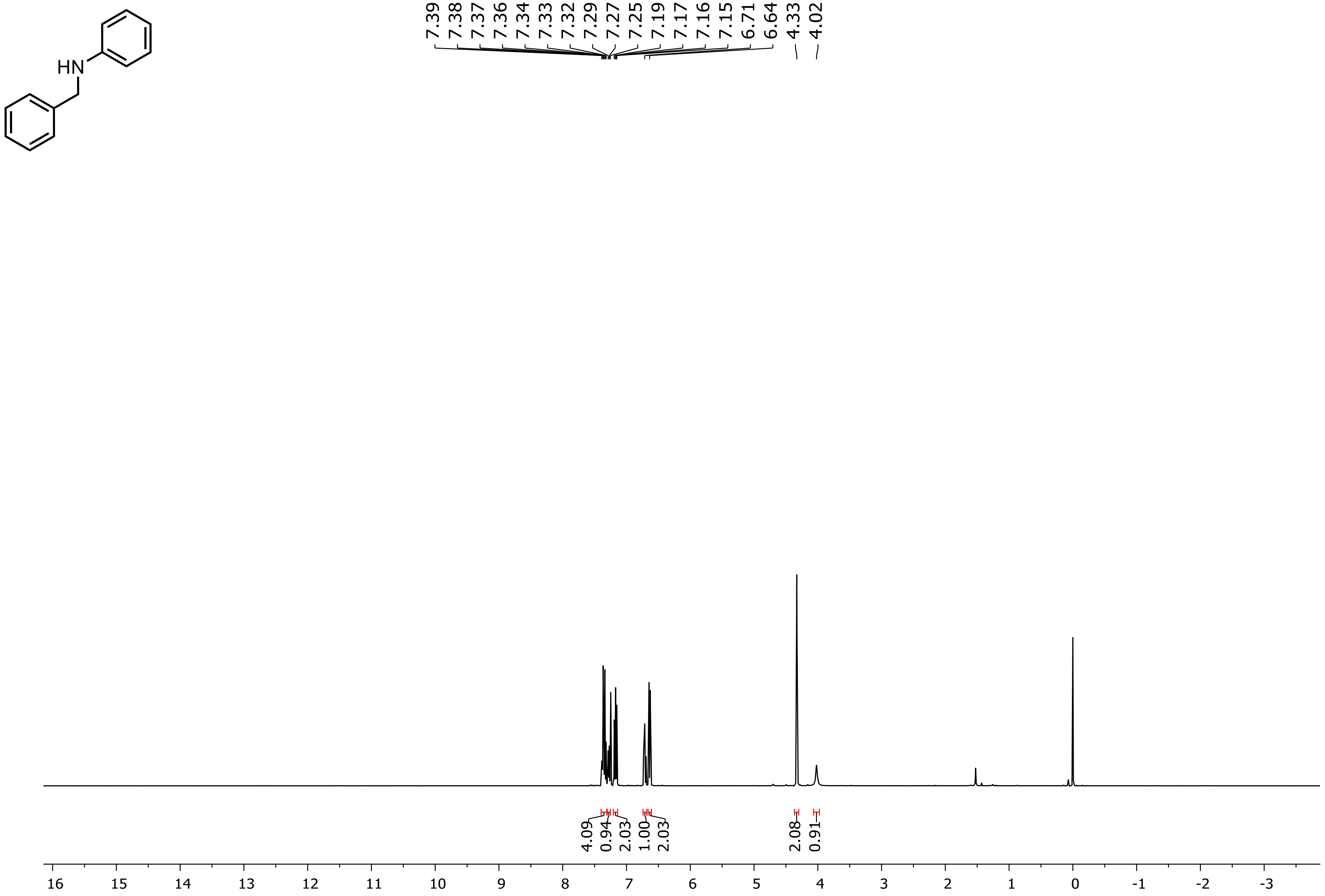

Figure S32 $^{13}C\{^1H\}$ **NMR** (101 MHz, $CDCl_3$, Me_4Si, 295K) spectrum of **7b**.

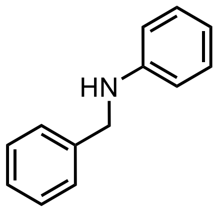

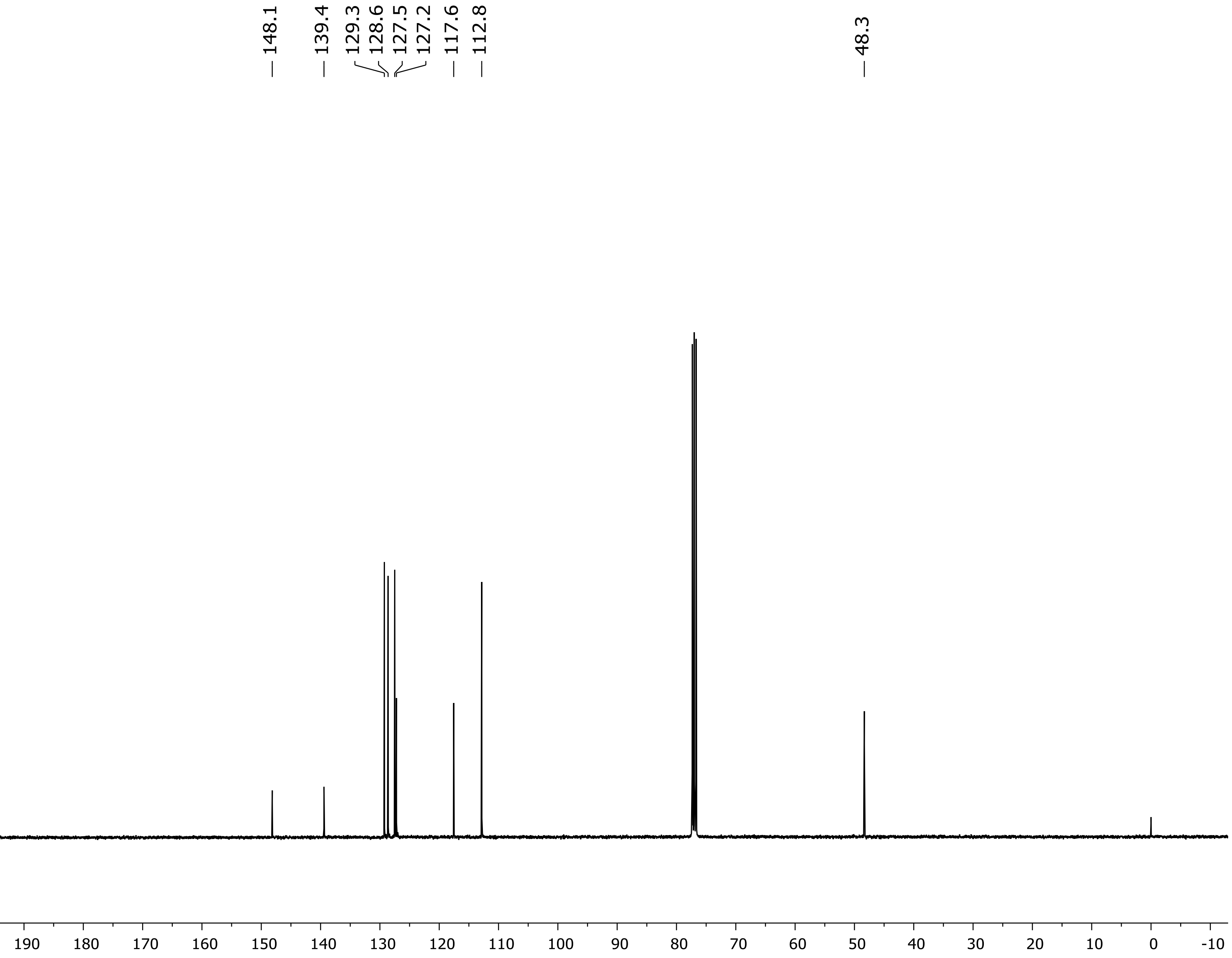

Figure S33 ^{1}**H NMR** (400 MHz, $CDCl_3$,Me_4Si, 295K) spectrum of **7c**.

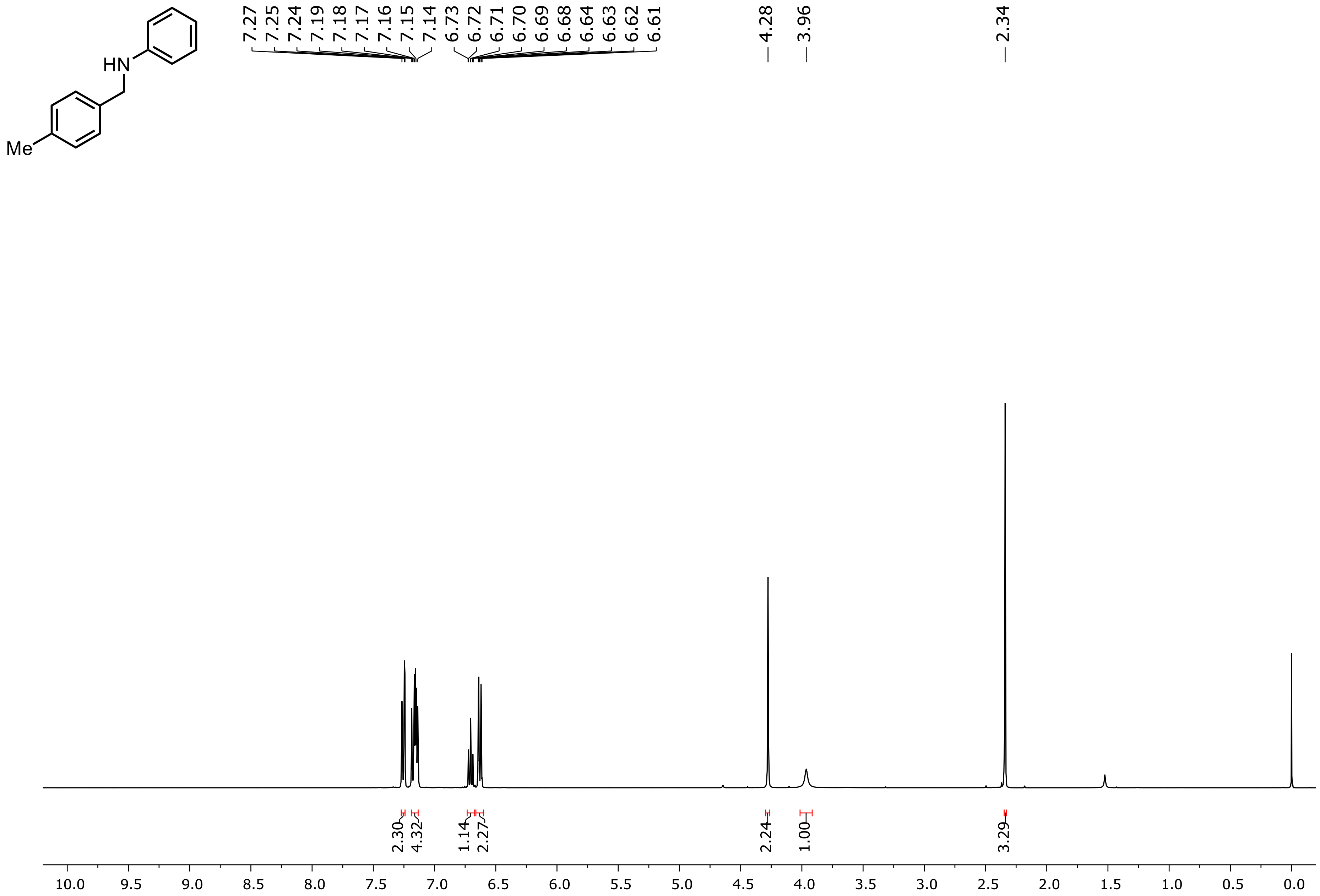

Figure S34 ^{13}C{^{1}H} NMR (101 MHz, $CDCl_3$, Me_4Si, 295K) spectrum of **7c**.

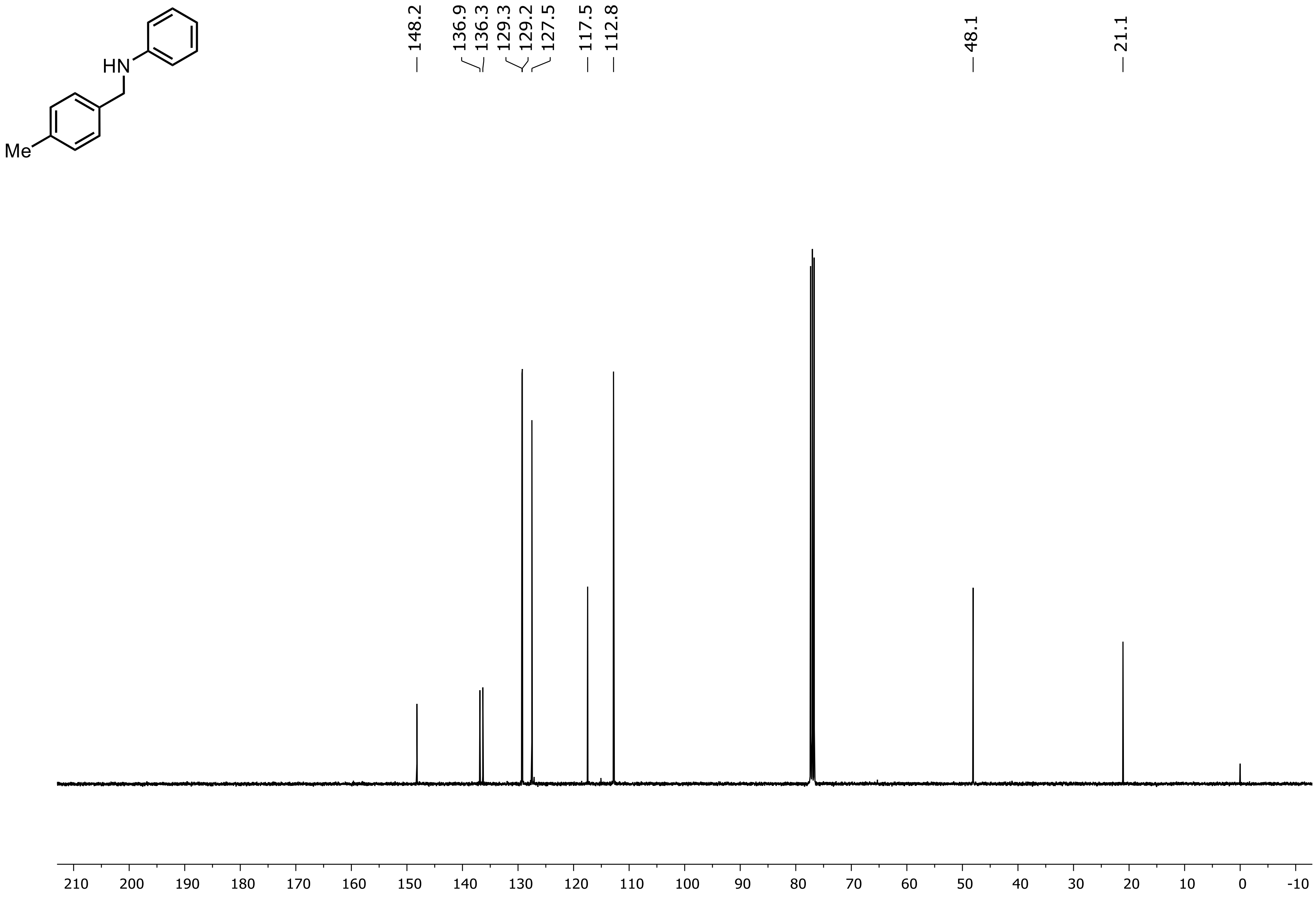

Figure S35 ^{1}**H NMR** (400 MHz, $CDCl_3$,Me_4Si, 295K) spectrum of **7d**.

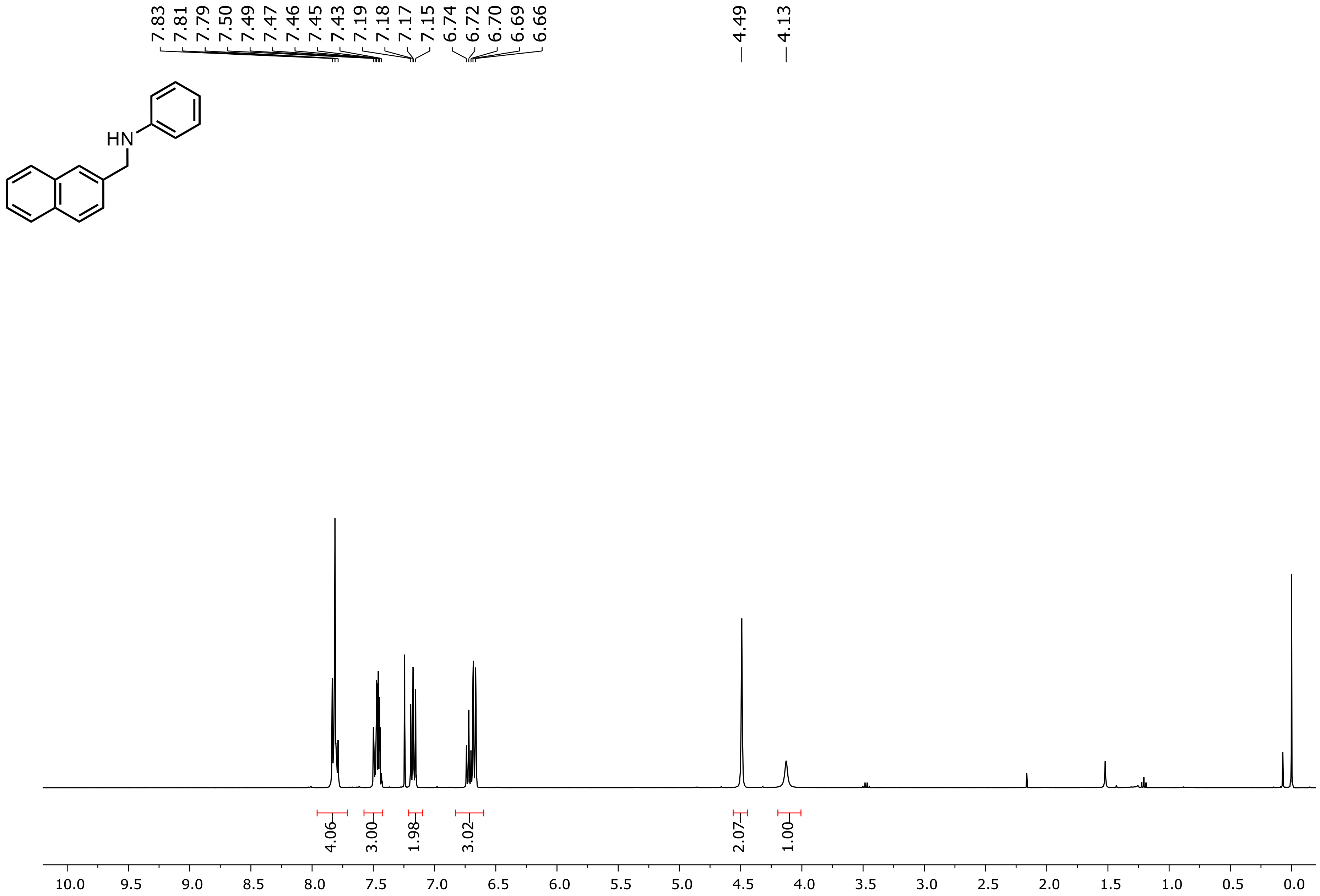

Figure S36 $^{13}C\{^{1}H\}$ NMR (101 MHz, $CDCl_3$, Me_4Si, 295K) spectrum of **7d**.

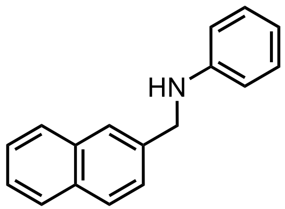

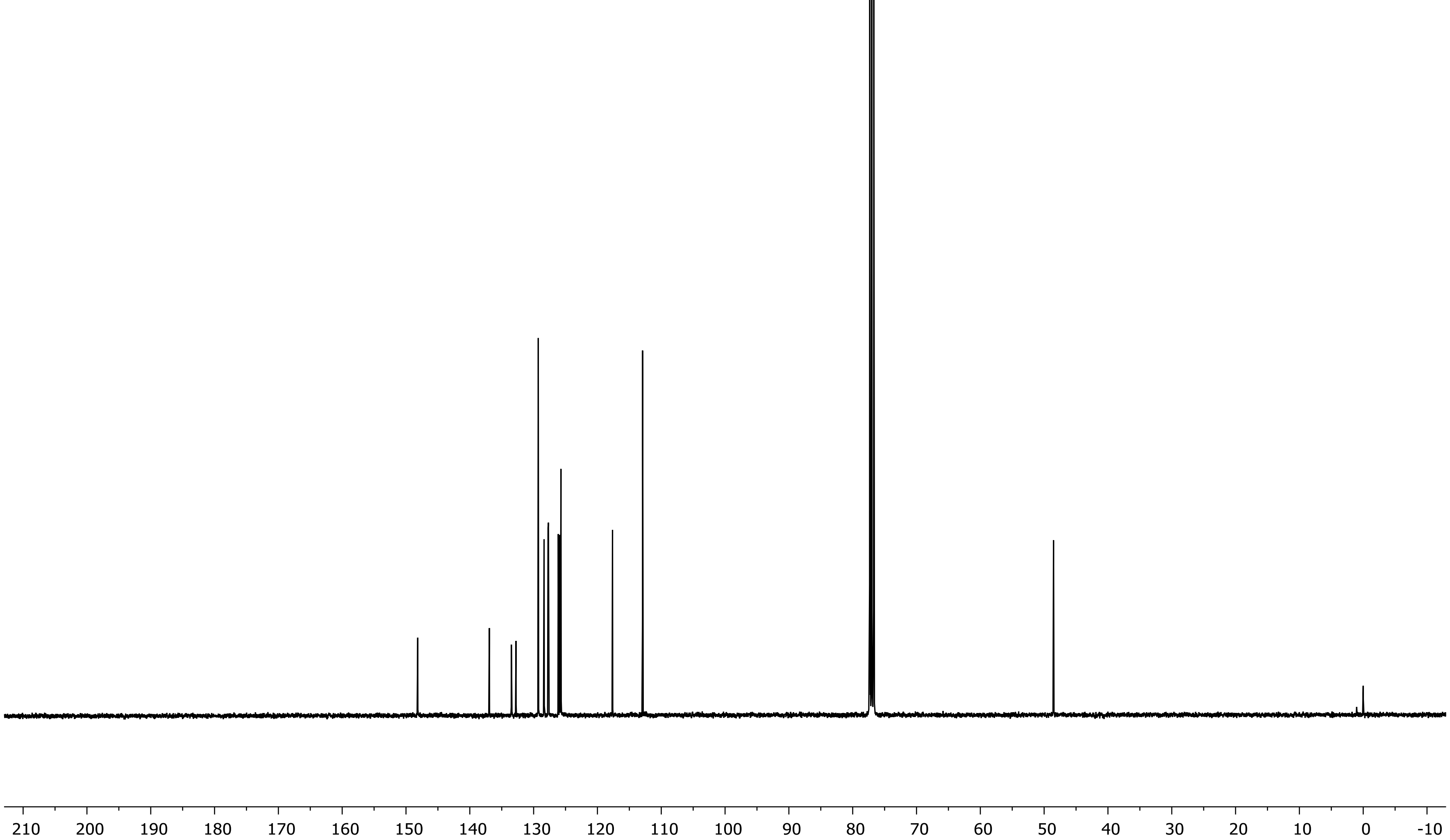

3 Crystallographic Data

All data were collected on a Bruker D8 Quest ECO diffractometer using graphite-monochromated Mo Kα radiation (λ = 0.71073 Å) and a Phvoton II-C14 CPAD detector. Crystals were mounted on Mitegen micromounts in NVH immersion oil, and all collections were carried out at 150 K using an Oxford cryostream. Data collections were carried out using φ and ω scans, with collections and data reductions carried out in the Bruker APEX-3 suite of programs.[10] Multi-scan absorption corrections were applied for all datasets using SADABS.[11] The data were solved with the intrinsic phasing routine in SHELXT,[12] and all data were refined on F2 with full-matrixleast-squares procedures in SHELXL,[13] operating within the OLEX-2 GUI.[14] All non-hydrogen atoms were refined with anisotropic displacement parameters. Carbon-bound hydrogen atoms were placed in riding positions and refined with isotropic displacement parameters equal to 1.2 or 1.5 times the isotropic equivalent of their carrier atom.

The model for compound **2** contains two lattice toluene molecules, one of which (C58-C64) overlaps a symmetry element and was modelled at 0.5 crystallographic occupancy with RIGU restraints to maintain sensible ADPs. The Fourier map for compound **4** contained regions of heavily disordered electron density, also overlapping symmetry elements, which could not be sensibly modelled as discrete atom positions. The electron density contribution from these regions was addressed with a solvent masking routine within Olex-2.[14] From the residual electron density we estimate the solvation of the bulk crystal at $0.25C_6H_{12}$ per formula unit (216 e^-/cell, *vs* 222 calculated)

3.1 X-ray refinement data

Table S1 Crystal data and structure refinement for compound **1** and **2**.

**Compound**	**1**	**2**
Empirical Formula	$C_{28}H_{26}BN$	$C_{50}H_{46}B_2N_2$
Formula Weight	387.31	834.71
Crystal System	Monoclinic	Triclinic
Space Group	$P2_1/c$	*P-1*
a/Å	8.6576(11)	10.84437(5)
b/Å	24.740(3)	14.0370(7)
c/Å	10.5153(13)	16.7577(9)
α/Å	90	87.7140(10)
β/Å	96.855(4)	78.7140(10)
γ/Å	90	70.8390(10)
V/Å^3	2236.1(5)	2362.7(2)
Z	4	2
T/K	150	150
D_C/g.cm^{-3}	1.150	1.173
Crystal size/mm	0.39x0.21x0.01	0.35x0.26x0.17
Reflections collected	33812	54302
Independent reflections	4093	10820
R_{int}	0.119	0.059
$R_1[F^2>2\ \sigma(F^2)]$	0.110	0.06
wR2 (all data)	0.241	0.159
GoF	1.06	1.021
r_{min}/r_{max}/eÅ^{-3}	-0.35/0.23	-0.31/0.64
CCDC code	2267646	2267647

Table S2 Crystal data and structure refinement for compound **3** and **4**.

**Compound**	**3**	**4**
Empirical Formula	C19 H15 Br2 N	$C_{32}H_{24}Br_4N_2$
Formula Weight	417.14	756.17
Crystal System	Monoclinic	Trigonal
Space Group	$P2_{1/c}$	*R-3*
a/Å	9.8335(4)	42.618(2)
b/Å	10.0697(4)	42.618(2)
c/Å	17.1387(7)	8.6216(7)
α/Å	90	90
β/Å	102.0840(10)	90
γ/Å	90	120
V/Å^3	1659.48(12)	13561.7(18)
Z	4	18
T/K	150	150
D_C/g.cm^{-3}	1.670	1.667
Crystal size/mm	0.2x0.09x0.07	0.12x0.07x0.07
Reflections collected	12839	70864
Independent reflections	3462	6163
R_{int}	0.0428	0.1591
$R_1[F^2>2\ \sigma(F^2)]$	0.0340	0.0586
wR2 (all data)	0.0552	0.1074
GoF	1.042	1.020
r_{min}/r_{max}/eÅ^{-3}	-0.555/0.0448	-0.550/0.49
CCDC code	2267648	2267649

Figure S37 Solid-state structure of compound **3**, thermal ellipsoids drawn at the 50% probability level. C: black, Br: red, N: blue. H-atoms omitted for clarity.

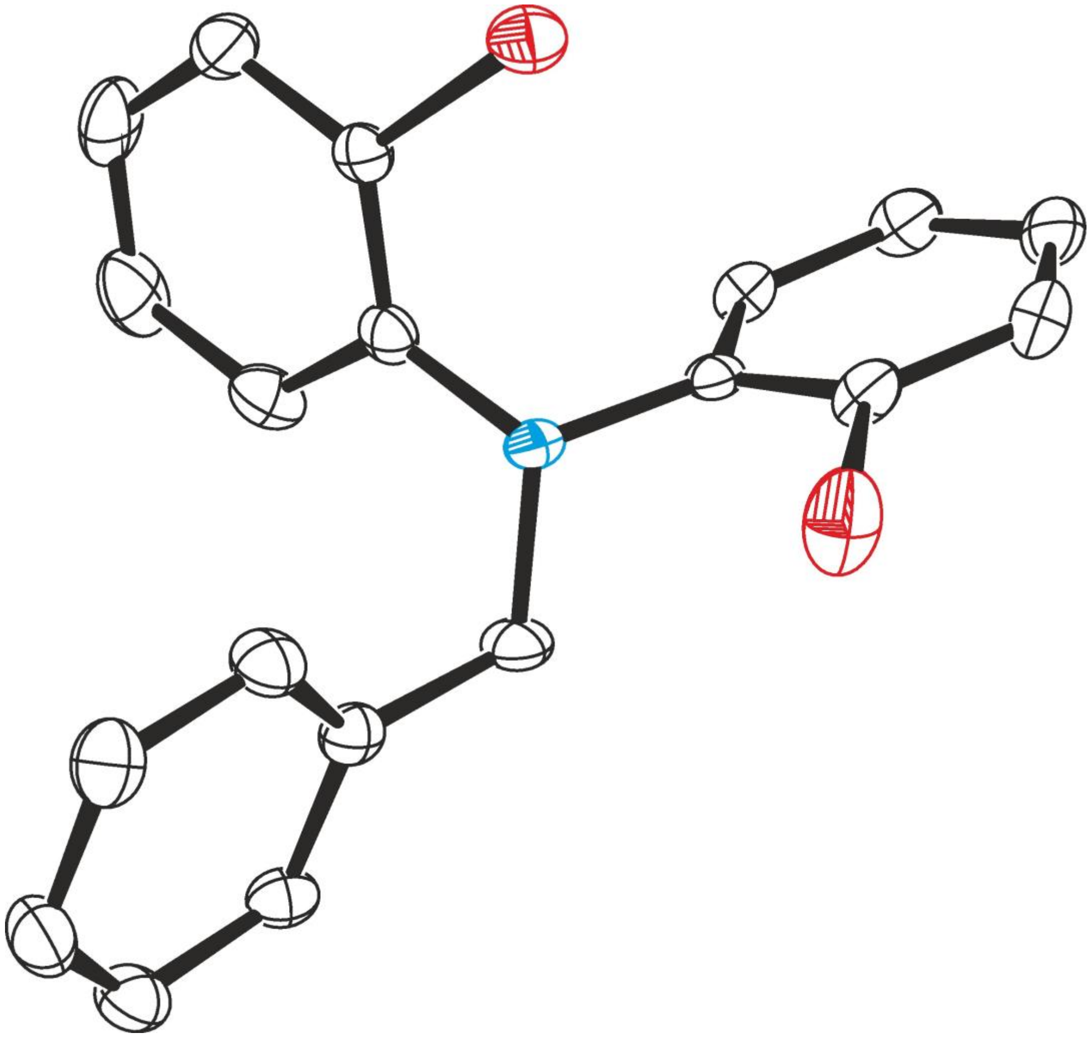

Figure S38 Solid-state structure of compound **4**, thermal ellipsoids drawn at the 50% probability level. C: black, Br: red, N: blue. H-atoms omitted for clarity.

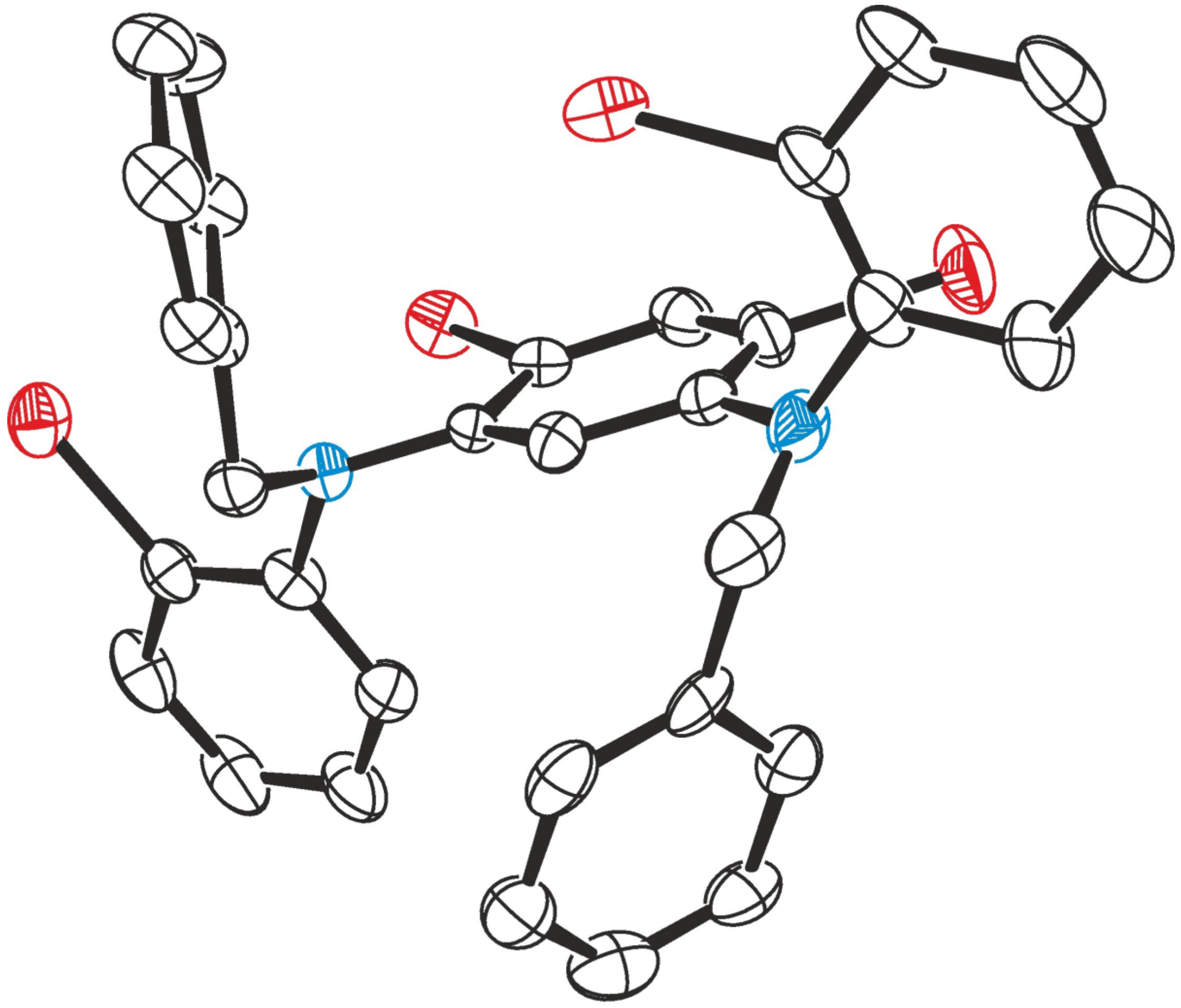

Figure S39 Key bond metrics of compound **1**. C: black, B: pink, N: blue.

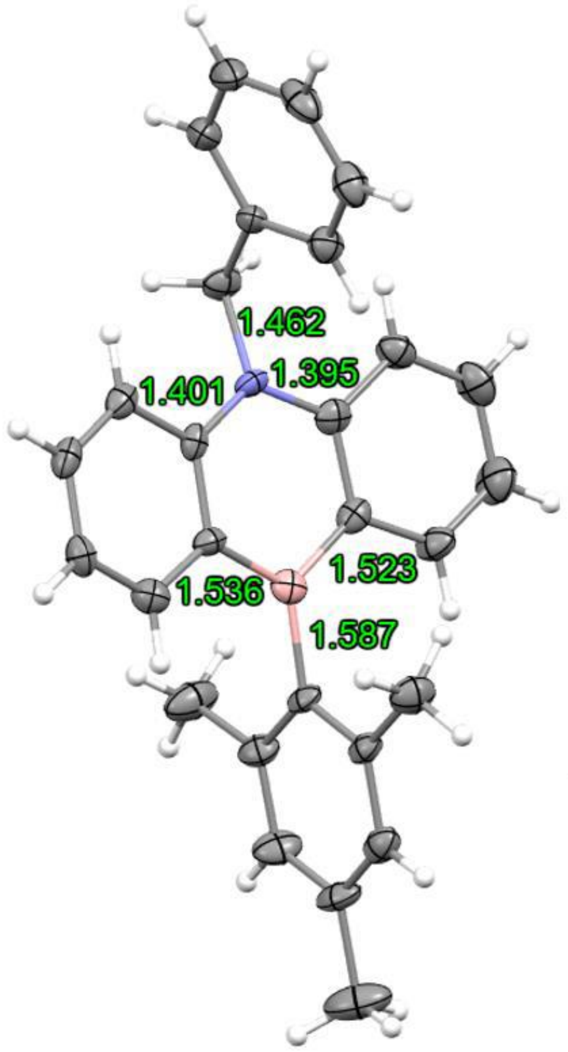

Figure S40 a) Bond metrics of compound **2**, b) Packing of **2** showing the CH interaction between the benzyl CH_2 and a neighbouring anthracene ring. C: black, B: pink, N: blue..

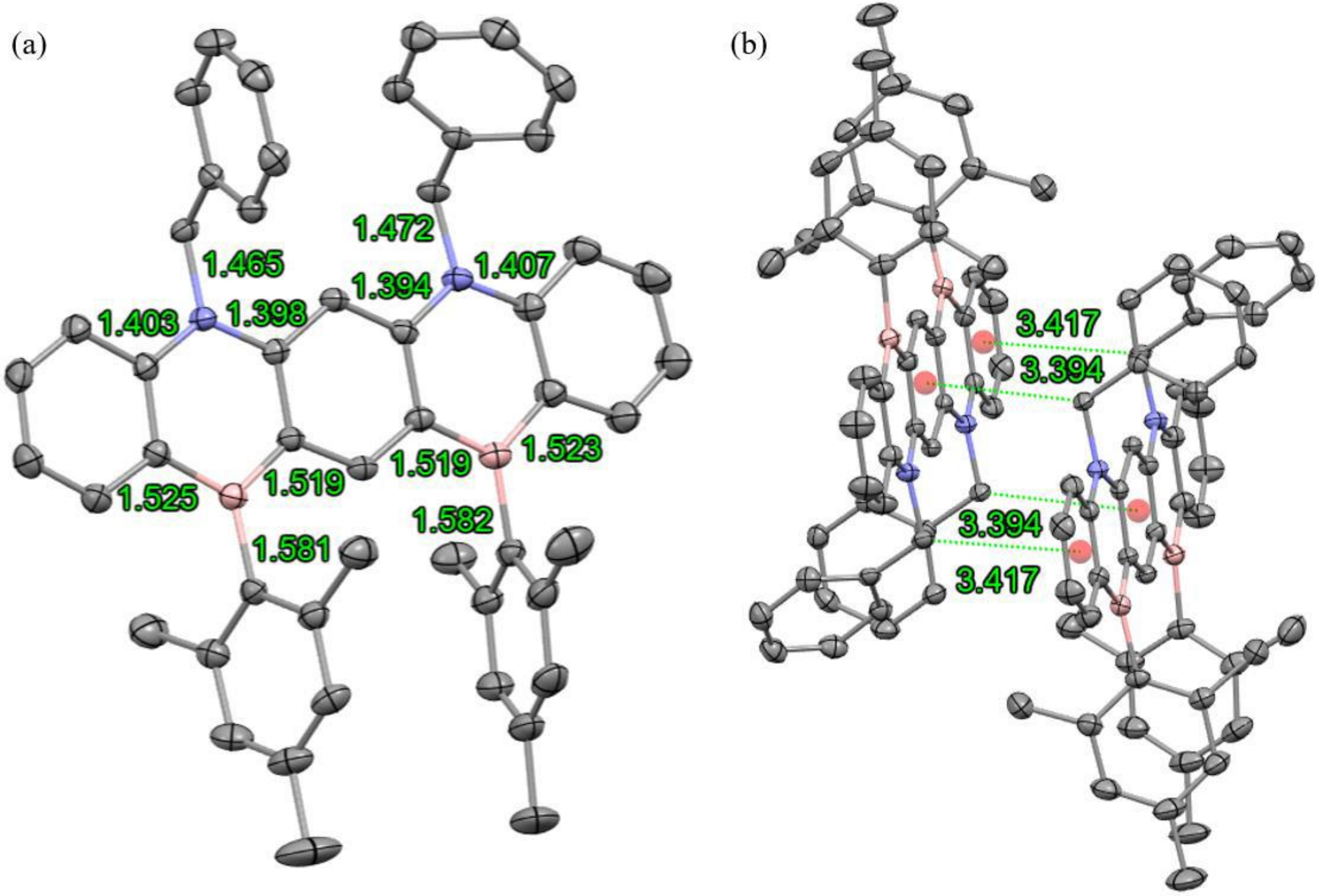

4 Cyclic Voltammetry

All electrochemical experiments were conducted on a Biologic SP-150e potentiostat. A three-electrode setup was used with a glassy carbon working electrode (3 mm diameter), a Pt counter electrode, and a silver wire pseudo reference in an Ar-purged cell. Ferrocene was added as an internal standard to correct the potential. The concentration of **1** was 10 mM, while **2** was less soluble, resulting in a lower concentration (approximately 1 mM) and consequently lower currents observed in CVs. The potential was scanned to oxidative potentials first for **1** which had no effect on the reduction peaks, but for **2** small changes were observed and therefore the potential was scanned cathodically starting from a potential more negative than the first observable oxidation peak. HOMO and LUMO values were determined from $E_{1/2}$ or Ep values from CVs.

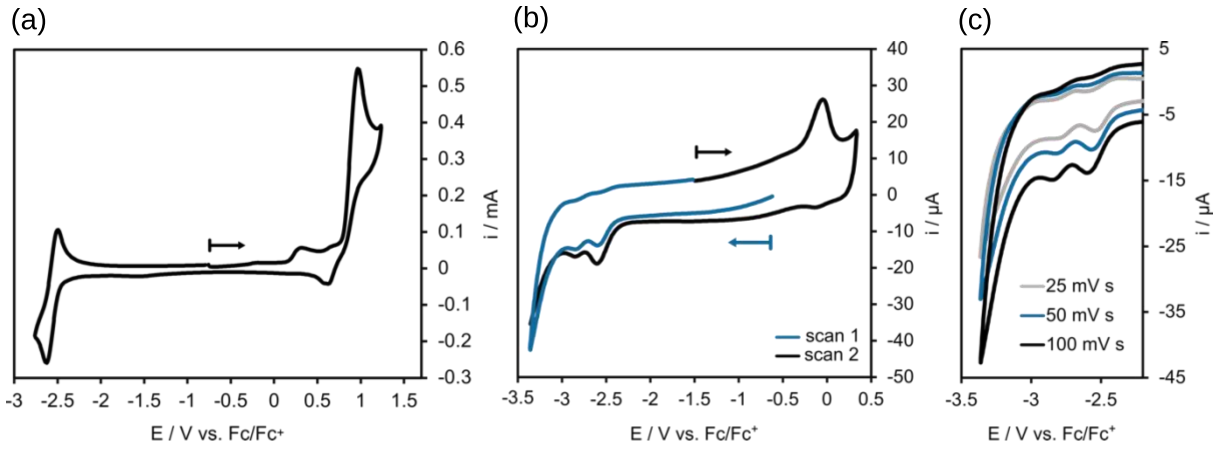

Figure S41 Cyclic voltammograms of (a) **1** and (b) **2** in dry acetonitrile containing $TBAPF_6$ (0.1 M) as a supporting electrolyte with a scan rate of 100 mV s^{-1}. Arrows represent the starting voltage and scan direction; (c) magnification of reduction of **2** with different scan rates displaying the two quasi-reversible redox features.

Table S3 Cyclic voltammetry data.

Compound	E_{red}/V vs. Fc/Fc$^+$	E_{ox}/V vs. Fc/Fc$^+$	HOMO/eV	LUMO/eV	E_g/eV
**1**	−2.57[a]	+0.33[b]	−5.13	−2.23	2.9
**2**	−2.61[b]; −2.85[b]	-0.03[b]	−4.77	−2.19	2.7

[a] *$E_{1/2}$ value used,* [b] *E_p values were uses as peaks were not fully reversible*

5 Computational Analysis

Computational analysis was carried out using the Orca package.[15–17] Geometries were optimised and orbitals determined using a threefold-corrected PBEh-3c/def2-mSVP composite method.[18–22]

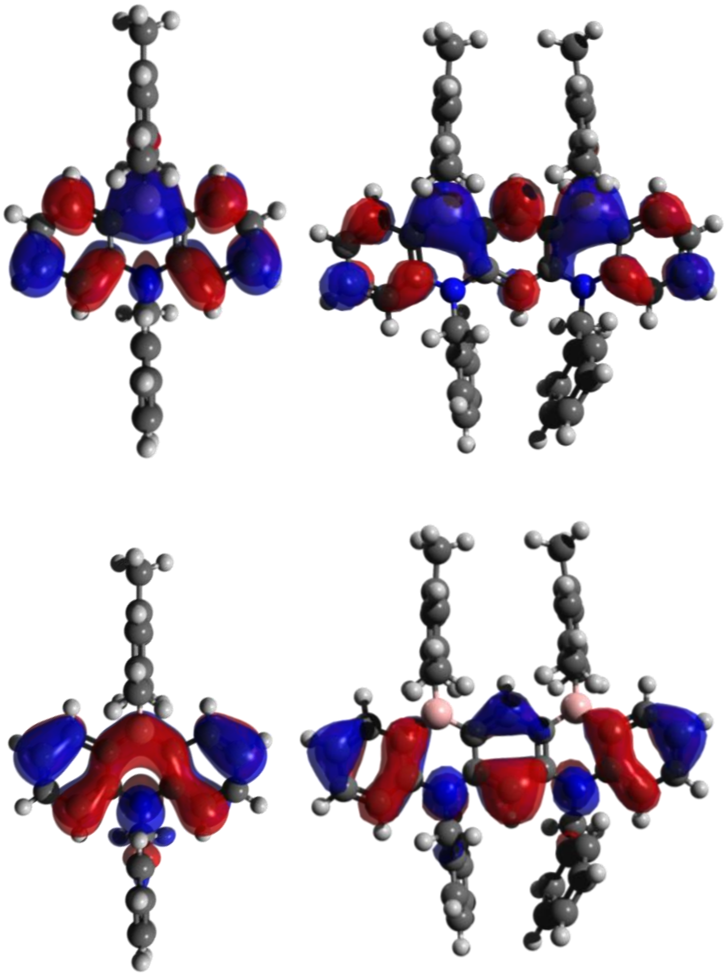

Figure S42 LUMO (top) and HOMO (bottom) of **1** (left) and **2** (right).

Table S4 Atomic coordinates of **1**.

Atom	x	y	z
C	1.253559	-1.03869	-1.07365
C	1.447384	0.336808	-0.95628
C	0.450376	1.092643	-0.38566
C	-0.74074	0.521905	0.090615
C	-0.9069	-0.87468	-0.00751
C	0.103835	-1.64267	-0.61434
B	-1.85662	1.374748	0.684402
C	-3.13501	0.616482	1.02564

C	-3.17635	-0.78441	0.875455
N	-2.05025	-1.49026	0.479647
C	-4.29137	1.279626	1.467417
C	-5.4608	0.608993	1.738765
C	-5.49374	-0.77229	1.552813
C	-4.38101	-1.46529	1.129148
C	-1.71423	2.923162	0.898661
C	-2.04411	-2.92899	0.647374
C	-2.54413	-3.72712	-0.53339
C	-2.05758	3.823697	-0.11446
C	-1.92328	5.192207	0.100559
C	-1.45618	5.696183	1.306091
C	-1.11606	4.792099	2.306111
C	-1.23911	3.42221	2.118822
C	-2.59712	-5.11458	-0.42812
C	-3.03852	-5.88897	-1.48758
C	-3.43515	-5.28336	-2.6722
C	-3.38769	-3.90336	-2.78263
C	-2.94462	-3.12756	-1.71948
C	-2.57574	3.314872	-1.43151
C	-0.85896	2.471372	3.21976
C	-1.33119	7.173961	1.539154
H	2.01035	-1.65209	-1.54584
H	2.356669	0.79581	-1.31963
H	0.56906	2.165999	-0.29412
H	-0.01069	-2.70507	-0.77468
H	-4.24151	2.355084	1.591266
H	-6.34133	1.138356	2.076415
H	-6.41084	-1.31925	1.730707
H	-4.47776	-2.52766	0.957365
H	-2.63096	-3.17332	1.534689
H	-1.02844	-3.23887	0.898456
H	-2.19104	5.881203	-0.69377
H	-0.74568	5.164927	3.255911
H	-2.28818	-5.59605	0.49334
H	-3.07278	-6.96608	-1.38976
H	-3.77991	-5.88511	-3.50255
H	-3.6972	-3.42219	-3.70105
H	-2.91386	-2.05022	-1.82079
H	-3.50275	2.751377	-1.30572
H	-1.86123	2.644485	-1.91384
H	-2.78129	4.129511	-2.12564
H	-0.54768	3.000983	4.119818
H	-0.03375	1.822312	2.918902
H	-1.69276	1.82081	3.491487
H	-1.50529	7.741409	0.625547

H	-0.33927	7.438139	1.908392
H	-2.05251	7.520511	2.28152

Table S5 Atomic coordinates of **2**

Atom	x	y	z
C	2.81007	-4.20673	-1.37465
C	3.993195	-4.41818	-0.67163
C	4.680373	-3.32316	-0.19677
C	4.233658	-2.01201	-0.41203
C	3.053811	-1.8152	-1.15862
C	2.338872	-2.93303	-1.61742
B	4.962007	-0.80291	0.174474
C	4.24847	0.524264	-0.02555
C	3.070932	0.59592	-0.80661
N	2.587807	-0.53393	-1.44172
C	4.714886	1.711275	0.527945
C	4.090048	2.944436	0.37849
C	2.880373	2.974235	-0.35491
C	2.394124	1.807571	-0.94766
C	6.320131	-0.87662	0.95727
C	1.622073	-0.36735	-2.51068
C	0.17095	-0.3465	-2.0926
C	6.336494	-1.05524	2.346401
C	7.542905	-1.02035	3.033755
C	8.748139	-0.80696	2.376126
C	8.722869	-0.64808	0.997708
C	7.529572	-0.67712	0.284396
C	-0.22029	-0.38957	-0.7615
C	-1.56609	-0.32335	-0.4232
C	-2.53291	-0.21779	-1.41011
C	-2.14809	-0.18359	-2.74392
C	-0.80609	-0.2457	-3.0793
C	5.047124	-1.26204	3.091427
C	7.537183	-0.46319	-1.2042
C	10.03392	-0.70919	3.144542
B	4.669442	4.221985	0.962069
C	3.885122	5.48982	0.622176
C	2.680912	5.391058	-0.10618
N	2.164244	4.151631	-0.47343
C	4.350301	6.761427	0.985355
C	3.677906	7.914466	0.646956
C	2.507801	7.802481	-0.09845
C	2.014036	6.571351	-0.47693
C	0.792863	4.056609	-0.94168
C	5.992696	4.192205	1.80565

C	5.944769	4.169156	3.20347
C	7.119915	4.035358	3.935585
C	8.35445	3.919713	3.312699
C	8.393907	3.964282	1.924572
C	7.236989	4.095358	1.168123
C	4.62305	4.26402	3.915359
C	7.326026	4.101668	-0.3337
C	9.613668	3.723837	4.106078
C	0.632111	3.958828	-2.43968
C	-0.46016	3.272089	-2.96001
C	-0.64211	3.167069	-4.33015
C	0.272139	3.743434	-5.19941
C	1.367634	4.422752	-4.68713
C	1.548397	4.529716	-3.3159
H	2.234594	-5.05156	-1.73091
H	4.353084	-5.42216	-0.4925
H	5.595242	-3.45917	0.368185
H	1.390814	-2.82677	-2.12568
H	5.628536	1.675	1.112489
H	1.488913	1.839732	-1.52774
H	1.856522	0.559996	-3.03939
H	1.787569	-1.15601	-3.24584
H	7.54274	-1.15105	4.111141
H	9.653843	-0.4856	0.464405
H	0.524043	-0.47523	0.019885
H	-1.85725	-0.35755	0.618444
H	-3.58045	-0.16824	-1.14407
H	-2.89567	-0.109	-3.52315
H	-0.511	-0.2049	-4.12173
H	4.371054	-0.41491	2.956735
H	5.214126	-1.3803	4.161696
H	4.518653	-2.15082	2.740448
H	8.551718	-0.42836	-1.60032
H	7.04831	0.477086	-1.47039
H	7.004827	-1.25767	-1.7311
H	10.09408	-1.46049	3.932554
H	10.12691	0.267844	3.624358
H	10.90121	-0.83697	2.497357
H	5.274614	6.81715	1.548567
H	4.054295	8.885817	0.937238
H	1.974105	8.693034	-0.40494
H	1.127193	6.552415	-1.09446
H	0.235042	4.908936	-0.55522
H	0.330305	3.186617	-0.46915
H	7.067001	4.010006	5.019048
H	9.350464	3.882353	1.418479

H	4.08099	5.170001	3.638816
H	4.749999	4.274396	4.997638
H	3.976137	3.418187	3.67082
H	7.019233	3.141318	-0.75527
H	8.344092	4.2929	-0.67263
H	6.683936	4.863983	-0.77832
H	9.440406	3.848434	5.174456
H	10.39104	4.430548	3.812304
H	10.01812	2.720735	3.95501
H	-1.16643	2.793731	-2.29019
H	-1.49514	2.624168	-4.71606
H	0.135839	3.6582	-6.26943
H	2.090347	4.86882	-5.35769
H	2.41789	5.049395	-2.93293

6 References

[1] C. Cremer, M. Goswami, C. K. Rank, B. de Bruin, F. W. Patureau, *Angew. Chem. Int. Ed.* **2021**, *60*, 6451.

[2] K. Thakur, D. Wang, S. V. Lindeman, R. Rathore, *Chem. - Eur. J.* **2018**, *24*, 13106.

[3] D. M. C. Ould, J. L. Carden, R. Page, R. L. Melen, *Inorg. Chem.* **2020**, *59*, 14891.

[4] A. M. Brouwer, *Pure Appl. Chem.* **2011**, *83*, 2213.

[5] D.-J. Kim, S.-H. Kim, T. Zyung, J.-J. Kim, I. Cho, S. K. Choi, *Macromolecules* **1996**, *29*, 3657.

[6] T. Agou, J. Kobayashi, T. Kawashima, *Org. Lett.* **2006**, *8*, 2241.

[7] S. R. Tamang, D. Bedi, S. Shafiei-Haghighi, C. R. Smith, C. Crawford, M. Findlater, *Org. Lett.* **2018**, *20*, 6695.

[8] E. P. Sánchez-Rodríguez, A. J. Fragoso-Medina, E. Ramírez-Meneses, M. Gouygou, M. C. Ortega-Alfaro, J. G. López-Cortés, *Catal. Commun.* **2018**, *115*, 49.

[9] D.-W. Tan, H.-X. Li, D. J. Young, J.-P. Lang, *Tetrahedron* **2016**, *72*, 4169.

[10] APEX-3, Bruker-AXS Inc., Madison, WI, **2016**.

[11] SADABS, Bruker-AXS Inc., Madison, WI, **2016**.

[12] G. M. Sheldrick, *Acta Crystallogr. Sect. Found. Adv.* **2015**, *71*, 3.

[13] G. M. Sheldrick, *Acta Crystallogr. Sect. C Struct. Chem.* **2015**, *71*, 3.

[14] O. V. Dolomanov, L. J. Bourhis, R. J. Gildea, J. A. K. Howard, H. Puschmann, *J. Appl. Crystallogr.* **2009**, *42*, 339.

[15] F. Neese, *WIREs Comput. Mol. Sci.* **2012**, *2*, 73.

[16] F. Neese, *WIREs Comput. Mol. Sci.* **2018**, *8*, e1327.

[17] F. Neese, F. Wennmohs, U. Becker, C. Riplinger, *J. Chem. Phys.* **2020**, *152*, 224108.

[18] H. Kruse, S. Grimme, *J. Chem. Phys.* **2012**, *136*, 154101.

[19] S. Grimme, S. Ehrlich, L. Goerigk, *J. Comput. Chem.* **2011**, *32*, 1456.

[20] S. Grimme, J. Antony, S. Ehrlich, H. Krieg, *J. Chem. Phys.* **2010**, *132*, 154104.

[21] S. Grimme, J. G. Brandenburg, C. Bannwarth, A. Hansen, *J. Chem. Phys.* **2015**, *143*, 054107.

[22] F. Weigend, *Phys. Chem. Chem. Phys.* **2006**, *8*, 1057.
